# Evaluating the clinical effectiveness and safety of various HER2-targeted regimens after prior taxane/trastuzumab in patients with previously treated, unresectable, or metastatic HER2-positive breast cancer: a systematic review and network meta-analysis

**DOI:** 10.1007/s10549-020-05577-7

**Published:** 2020-02-25

**Authors:** Noman Paracha, Adriana Reyes, Véronique Diéras, Ian Krop, Xavier Pivot, Ander Urruticoechea

**Affiliations:** 1 F. Hoffmann-La Roche AG, Grenzacherstrasse 124, 4070 Basel, Switzerland; 2 Centre Eugène Marquis, Rennes, France; 3 Dana-Farber Cancer Institute, Harvard Medical School, Boston, MA USA; 4 Paul Strauss Centre, Regional Institute of Cancer, Strasbourg, France; 5 Onkologikoa Foundation, San Sebastián, Spain

**Keywords:** Trastuzumab emtansine, Capecitabine, Lapatinib, Neratinib, Pertuzumab, Locally advanced

## Abstract

**Purpose:**

In the absence of head-to-head trial data, network meta-analysis (NMA) was used to compare trastuzumab emtansine (T-DM1) with other approved treatments for previously treated patients with unresectable or metastatic HER2-positive breast cancer (BC).

**Methods:**

Systematic reviews were conducted of published controlled trials of treatments for unresectable or metastatic HER2-positive BC with early relapse (≤ 6 months) following adjuvant therapy or progression after trastuzumab (Tras) + taxane published from January 1998 to January 2018. Random-effects NMA was conducted for overall survival (OS), progression-free survival (PFS), overall response rate (ORR), and safety endpoints.

**Results:**

The NMA included regimens from seven randomized controlled trials: T-DM1 and combinations of Tras, capecitabine (Cap), lapatinib (Lap), neratinib, or pertuzumab (Per; unapproved). OS results favored T-DM1 over approved comparators: hazard ratio (HR) (95% credible interval [95% CrI]) vs Cap 0.68 (0.39, 1.10), LapCap 0.76 (0.51, 1.07), TrasCap 0.78 (0.44, 1.19). PFS trends favored T-DM1 over all other treatments: HR (95% CrI) vs Cap 0.38 (0.19, 0.74), LapCap 0.65 (0.40, 1.10), TrasCap 0.62 (0.34, 1.18); ORR with T-DM1 was more favorable than with all approved treatments. In surface under cumulative ranking curve (SUCRA) analysis T-DM1 ranked highest for all efficacy outcomes. Discontinuation due to adverse events was less likely with T-DM1 than with all comparators except neratinib. In general, gastrointestinal side effects were less likely and elevated liver transaminases and thrombocytopenia more likely with T-DM1 than with comparators.

**Conclusions:**

The efficacy and tolerability profiles of T-DM1 are generally favorable compared with other treatments for unresectable or metastatic HER2-positive BC.

**Electronic supplementary material:**

The online version of this article (10.1007/s10549-020-05577-7) contains supplementary material, which is available to authorized users.

## Introduction

Human epidermal growth factor receptor 2 (HER2) is a receptor tyrosine-protein kinase, which is widely expressed and can promote tumorigenesis when expression is increased [1]; approximately 15% of breast cancer (BC) cases are HER2-positive, classified by HER2 protein overexpression or HER2 gene amplification [2]. HER2-positive BC has an aggressive clinical phenotype with, historically, a poor prognosis [3]. However, since its approval in 1998, the HER2-targeted humanized monoclonal antibody trastuzumab (Herceptin^®^; Roche) [4, 5] has been associated with significant and clinically relevant improvements in disease-free and overall survival (OS) [6].

Trastuzumab emtansine (T-DM1; Kadcyla^®^; Roche) is a first-in-class antibody–drug conjugate approved for the treatment of HER2-positive unresectable locally advanced BC (LABC) or metastatic BC (mBC) in patients previously treated with trastuzumab and a taxane, separately or in combination [7]. T-DM1 is a conjugate of DM1, a cytotoxic derivative of maytansine, and has the HER2-targeting and cytotoxicity-mediating properties of trastuzumab [8]. Conjugated DM1 is released to exert cytotoxic effects when the antibody–drug conjugate is internalized by the cell to which it binds [9].

Regulatory approval of T-DM1 for use in the mBC setting was based on improvements in progression-free survival (PFS) and in OS in the phase 3 EMILIA study; EMILIA was a multicenter, open-label randomized controlled trial (RCT) that evaluated the efficacy and safety of T-DM1 compared with capecitabine plus lapatinib in patients with HER2-positive LABC or mBC, who were previously treated with trastuzumab and a taxane [10]. T-DM1 received US Food and Drug Administration (FDA) approval in February 2013 [11] and European Medicines Agency (EMA) marketing authorization in November 2013 [12]. A recent descriptive analysis of EMILIA, which followed up patients who crossed over from the control group to T-DM1, corroborated the original findings of improved OS with T-DM1 relative to control [13]. Additionally, based on recent data from the KATHERINE trial [14], in May 2019, T-DM1 was approved by the FDA for use in the early BC setting for patients with residual invasive disease after neoadjuvant taxane and trastuzumab-based treatment [15].

Several other therapies are available, or have been studied in patients with previously treated, unresectable, HER2-positive LABC or mBC [16], including trastuzumab in combination with capecitabine [17] or vinorelbine [18], and the tyrosine kinase inhibitors lapatinib (Tyverb^®^; Novartis—in combination with capecitabine; LapCap) [19, 20] and neratinib (Nerlynx^®^; Puma Biotechnology, Inc.) [21, 22]. In the absence of direct head-to-head evidence from clinical trials, the relative efficacy of treatments can be assessed using network meta-analysis (NMA) to combine direct and indirect evidence from multiple independent trials [23]. Therefore, we conducted a systematic review (SR) and NMA to evaluate the clinical effectiveness and safety of T-DM1 versus other treatments for HER2-positive mBC. The monoclonal antibody pertuzumab (Perjeta^®^; Roche—treatment in combination with trastuzumab and chemotherapy) [24, 25] was included in the NMA for completeness despite not being approved in this patient group. The primary endpoint analysis of the failed PHEREXA trial showed pertuzumab to increase PFS, but was not statistically significant, when added to trastuzumab plus capecitabine [26].

## Methods

All SR and NMA methodology and reporting complied with Preferred Reporting Items for Systematic Reviews and Meta-analyses (PRISMA) guidelines.

### Systematic review

The SR included published data between 1 January 1998 and 3 January 2018 based on searches of MEDLINE^®^, EMBASE™, MEDLINE^®^ In-Process, the Cochrane Central Register of Controlled Trials (CENTRAL), Cochrane Methods studies, the Cochrane Database of Systematic Reviews (CDSR), and the Database of Abstracts of Reviews of Effects (DARE). Database searches were complemented by manual searches of conference abstracts from the American Society of Clinical Oncology (ASCO), the European Society for Medical Oncology (ESMO), the San Antonio Breast Cancer Symposium (SABCS), and the International Society for Pharmacoeconomics and Outcomes Research (ISPOR). Studies in the SR were identified based on search strings provided in Online Resource 1. The SR searched for studies from three separate periods (1 January 1998–2 July 2013, 1 October 2012–30 June 2016, and 1 January 2016–3 January 2018), with sufficient overlap in time between the search periods to allow for indexing delays of published studies. Citations were reviewed by two independent reviewers, and discrepancies adjudicated by a third independent reviewer according to eligibility criteria presented in Online Resource 2. Eligible studies were controlled trials of pharmacological treatments for HER2-positive LABC with early relapse (within 6 months) following adjuvant therapy, or for HER2-positive unresectable mBC in which patients had progressed after treatment with trastuzumab plus taxane. No pre-specified interventions or comparators were targeted, interventions were not required to be approved for use in this indication, and trials were included independent of randomization, phase, or blinding status. Critical appraisal of included trials was based on recommendations from the UK National Institute for Health and Care Excellence (NICE), Germany’s Institute for Quality and Efficiency in Health Care (IQWIG), the Canadian Agency for Drugs and Technologies in Health (CADTH), the French National Authority for Health (HAS; randomized trials), and the Downs and Black checklist (non-randomized trials) (Online Resource 3).

### Network meta-analysis

#### Treatment networks

Not all studies reported all selected endpoints. Separate network plots were developed for OS, PFS, overall response rate (ORR), and the following safety-related endpoints (all based on treatment-related adverse events [AEs] Common Terminology Criteria for Adverse Events [CTCAE] grade 3 [severe] and above [grade 4, life-threatening or disabling; grade 5, death-related]): number of patients with AEs; serious AEs; treatment discontinuation due to AEs; and selected individual AEs, namely anemia, diarrhea, fatigue, increased alanine aminotransferase (ALT), increased aspartate aminotransferase (AST), mucosal inflammation, nausea, neutropenia, palmar–plantar erythrodysesthesia (PPE), thrombocytopenia, and vomiting.

#### Statistical methodology

A Bayesian NMA of PFS, OS, ORR, and safety endpoints was conducted on a log-hazard (PFS and OS) or log odds scale (ORR, safety endpoints). A Bayesian inferential framework was used, because it captures and propagates uncertainty while allowing external information to be included [27]. The NMA was conducted using the GeMTC R package in the Roche Biometrics Experimental Environment (BEE; R version 3.4.4) [28, 29]; both fixed-effects and random-effects approaches were used [23]. Markov Chain Monte Carlo (MCMC) convergence was assessed by inspecting trace plots and Brooks–Gelman–Rubin statistics [30]. The detailed methodology is included in Online Resource 4 [31]. Data inputs used to calculate hazard ratios (HRs) for PFS and OS, odds ratios (ORs) for ORR and AEs, and their respective 95% credible intervals (CrIs) are included in Online Resource 5. For all endpoints, the primary analyses were based on data as reported in the relevant RCTs; for Roche-sponsored trials (EMILIA [10], GBG 26 [32], and PHEREXA [26]), unpublished data were included where relevant.

A sensitivity analysis used the treatment crossover-adjusted HR for OS, calculated using either the rank-preserving structural-failure time model (RPSFTM) [33–35] or a Cox regression model with treatment crossover as a time-dependent covariate [36]. Although there is no one ideal method for adjusting for crossover [35], RPSFTM is one of the preferred methods that preserves randomization and is least prone to selection bias [33–35]; whereas use of a Cox regression model with treatment crossover as a time-dependent covariate was found to be the most robust among several approaches reported in an analysis of a phase 3 trial of LapCap versus capecitabine, and was considered to provide a more balanced result [36]. Overall rankings for each treatment were determined by estimation of Surface Under the Cumulative Ranking Curve (SUCRA; range 0–100%) [37].

## Results

### Study selection and heterogeneity assessment

**Fig. 1:**
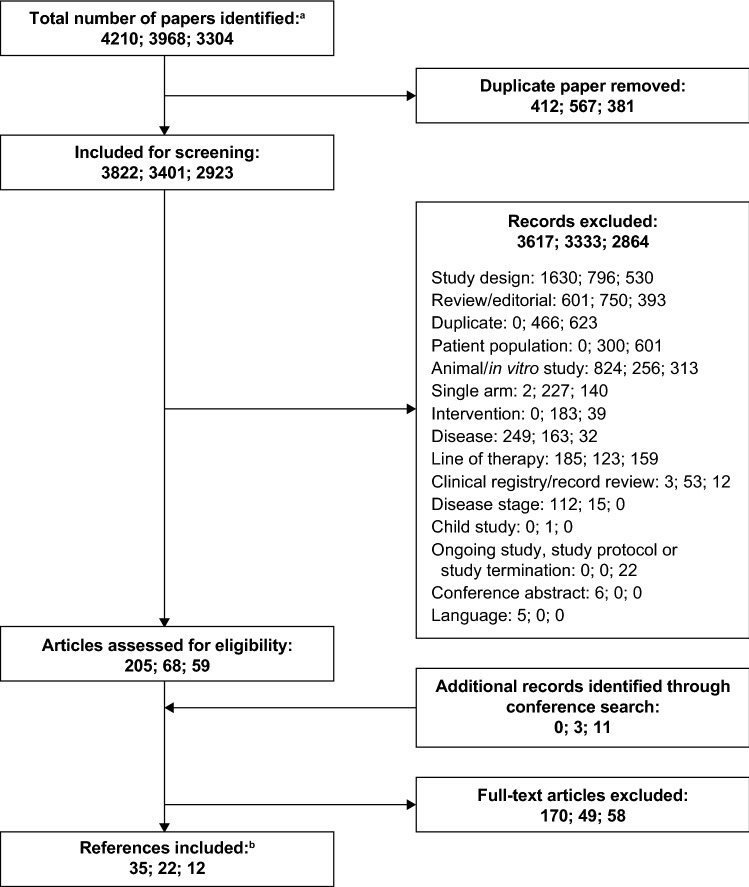
PRISMA diagram of included and excluded studies (^a^Respectively, the record numbers shown are those from the initial SR covering 1 January 1998 to 2 July 2013, from the first update of the SR, covering 1 October 2012 to 30 June 2016, and from the second update of the SR, covering 1 January 2016 to 3 January 2018. ^b^For each clinical study only the primary references, and not conference abstracts, are cited in the text). A PRISMA checklist is provided in Online Resource 11. *PRISMA* Preferred Reporting Items for Systematic Reviews and Meta-analyses, *SR* systematic review

The results of the three rounds of SR are summarized in Fig. 1. The initial SR covered searches from 1 January 1998 to 2 July 2013. After deduplication, 3822 records were identified for screening; from which five RCTs were identified for analysis: EMILIA [10]; GBG 26 [32]; EGF100151 [38]; Martin et al. [39]; and CEREBEL [17]. The first update of the SR encompassed searches from 1 October 2012 to 30 June 2016. After deduplication, 3401 records were identified for screening, from which updates to the original five RCTs and one new RCT were identified: PHEREXA [26]. The second update to the SR covered searches from 1 January 2016 to 3 January 2018. After deduplication, 2923 records were identified for screening, from which updates and one further RCT, ELTOP [40], were identified.

Overall, seven RCTs met the criteria for inclusion in the NMA: EMILIA [10]; GBG 26 [32]; EGF100151 [38]; a phase 2 trial of neratinib versus lapatinib plus capecitabine (Martin et al. 2013) [39]; PHEREXA [26]; ELTOP [40]; and a prior trastuzumab treatment subgroup in CEREBEL [17]—although CEREBEL did not meet the inclusion criteria, it included a subgroup defined as “patients who received prior trastuzumab either in adjuvant or metastatic setting” and was thus included in the NMA. The phase 3 TH3RESA study [41] that evaluated T-DM1 was excluded from the NMA because of limitations that its inclusion would impose i.e., only patients on certain treatment regimens in the comparator arm “Physician’s choice” were relevant to the NMA, and disaggregating data on particular treatments from this arm would have broken randomization. Thus, selecting a particular treatment from “Physician’s choice” would introduce bias and break the fundamental principle of NMA which relies on randomized evidence.

Eligibility criteria for inclusion in the NMA are summarized in Online Resource 2, and trial information and patient characteristics at baseline in the trials, which were included in the analysis, are summarized in Table 1. Although there were differences in population size and treatment line among studies, heterogeneity assessment indicated that all trials were comparable in terms of randomization, allocation concealment, demographic and baseline characteristics, outcome selection and reporting, patient withdrawal from the studies, and statistical analyses undertaken (Online Resource 3). In total, there were five phase 3 studies and two phase 2 studies. All were open-label, but EMILIA and EGF100151 used independent review committees to assess outcomes and, therefore, the outcome assessors were blinded to study treatment. Patients from all studies had been treated previously with trastuzumab; however, only results from a “prior trastuzumab treatment” subgroup were included from the CEREBEL study. Results of critical appraisal of trials are presented in Online Resource 3.

**Table 1:** Trial methodologies and baseline characteristics

	EMILIA [10] NCT00829166	GBG 26 [32] NCT00148876	EGF100151 [36, 38] NCT00078572	Martin et al., 2013 [39] NCT00777101	CEREBEL trial subgroup^a^ [17] NCT00820222	PHEREXA [26] NCT01026142	ELTOP [40]
Intervention	T-DM1 (*N* = 495)	Cap + trastuzumab (*N* = 78)	Cap + lap (*N* = 198)	Neratinib (*N* = 117)	Cap + lap (*N* = 271)	Pertuzumab + trastuzumab + cap (*N* = 228)	Cap + trastuzumab (*N* = 43)
Comparator	Cap + lap (*N* = 496)	Cap (*N* = 78)	Cap (*N* = 201)	Cap + lap (*N* = 116)	Cap + trastuzumab (*N* = 269)	Cap + trastuzumab (*N* = 224)	Cap + lap (*N* = 43)
Crossover permitted, *N* (%)	Yes (27%) [13]	No	Yes (18%)^j^ [36]	No	No	No	No
Present line of treatment, *N* (%)							
1L	0	0	NR	0	238 (44)	0	5 (6)
1L-R	118 (12)	0	NR	0	NR	0	NR
2L	361 (36)	156 (100)	393 (98)	32 (14)	302 (56)^b^	449 (100)^c^	61 (71)
3L or later	512 (52)	NR	NR	200 (86)^b^	NR	0	20 (23)
Age, median years (range)	I: 53 (25–84) C: 53 (24–83)	I: 53 (28–78) C: 59 (33–82)	I: 54 (26–80) C: 51 (28–83)	I: 52 (28–79) C: 56 (30–79)	I: 53 (27–83) C: 56 (31–79)	I: 54 (NR) C: 55 (NR)	I: 57 (34–81) C: 59 (37–78)
ECOG performance status = 1, *N* (%)	I: 194 (39)^d^ C: 176 (35)^d^	NR	I: 76 (38) C: 83 (41)	I: 43 (37) C: 39 (34)	I: 260 (96)^e^ C: 261 (98)^e^	I: 68 (30) C: 73 (33)	I: 18 (42) C: 12 (28)
ER+ and/or PR+ tumors, *N* (%)	I: 282 (57)^f^ C: 263 (53)^f^	I: 41 (56)^g^ C: 43 (62)^g^	I: 96 (48) C: 93 (46)	I: 52 (44) ER+; 31 (27) PR+ C: 46 (40) ER+; 32 (28) PR+	I: 133 (49) ER+; 98 (36) PR+ C: 122 (45) ER+; 80 (30) PR+	I: 126 (55) C: 123 (55)	I: 27 (63) C: 27 (63)
Time since initial diagnosis, median years (range)	C: 3.1 (0.1–29.8) I: 3.3 (0.2–31.6)	NR	I: 3.8 (0–21)^h^ C: 4.1 (0–19)	NR	I: 2.6 (0–18) C: 3.0 (0–25)	NR	NR
Time since first metastases, median years (range)	C: 1.5 (0.04–15.1) I: 1.3 (0.03–24.3)	NR	I: 1.70 (0–9) C: 1.60 (0–8)	NR	NR	NR	NR
Advanced or metastatic sites in the brain, *N* (%)	C: 45 (9) I: 50 (10)	I: 1 (1) C: 2 (3)	NR	NR	I: 0 C: 0	I: 25 (11) C: 28 (13)	I: 6 (14) C: 7 (16)
Visceral disease, *N* (%)	I: 334 (67) C: 335 (68)	NR	I: 153 (77) C: 158 (79)	NR	I: 173 (64) C: 164 (61)	I: 148 (65)^i^ C: 146 (65)^i^	NR

*1L* first line, *1L-R* first-line relapse, *2L* second line, *3L* third line, *C* comparator, *Cap* capecitabine, *CNS* central nervous system, *ECOG* Eastern Cooperative Oncology Group, *ER* estrogen receptor, *GBG* German Breast Group, *I* intervention, *Lap* lapatinib, *NR* not reported, *PR* progesterone receptor, *T-DM1* trastuzumab emtansine

^a^The information shown is for the total intent-to-treat population for CEREBEL; however, analysis of progression-free and overall survival is based on only the subgroup of patients who received prior trastuzumab in the adjuvant or metastatic setting (*N* = 167 for cap + lap and *N* = 159 for cap + trastuzumab)

^b^Patients with two or three or more prior anti-cancer regimens

^c^100% of patients received trastuzumab in the 1L metastatic breast cancer setting; information on prior trastuzumab setting missing in three patients

^d^ECOG performance status not available for eight and two patients in cap + lap and T-DM1 groups, respectively

^e^ECOG performance status of 0 or 1

^f^Hormone receptor status unknown for nine and 11 patients in cap + lap and T-DM1 groups, respectively

^g^Hormone receptor status was not reported for five and seven patients in the cap + trastuzumab and cap groups, respectively

^h^Value based on *N* = 207; 198 patients were randomized to cap + lap and nine additional patients were assigned the treatment later on during the trial

^i^Percentage based on a total *N* of 223 and 226 in the cap + trastuzumab and pertuzumab + trastuzumab + cap groups, respectively

^j^Percentage based on a total *N* of 201 and a total of 36 patients that crossed over to the combination arm

### Treatment networks

**Fig. 2:**
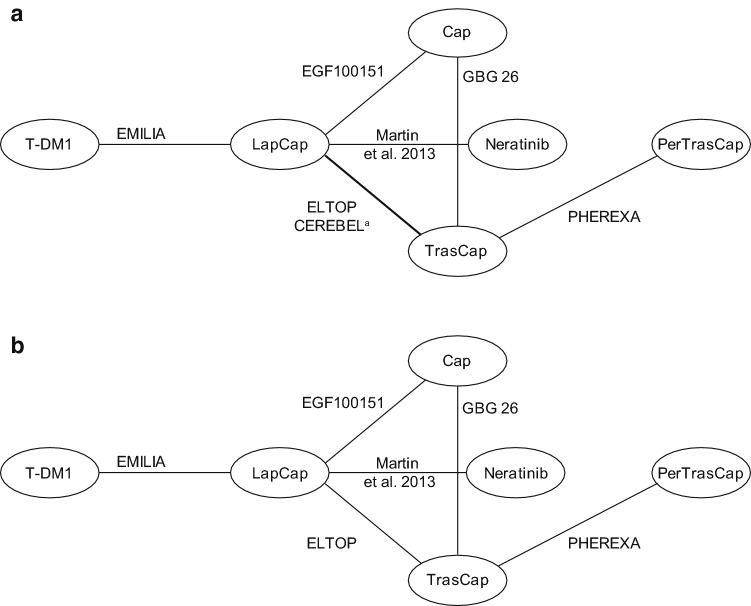
Treatment network plots for **a** OS, adjusted OS, PFS, and **b** ORR. *Cap* capecitabine, *Lap* lapatinib, *ORR* overall response rate, *OS* overall survival, *Per* pertuzumab, *PFS* progression-free survival, *T-DM1* trastuzumab emtansine, *Tras* trastuzumab

Seven studies reported data for OS, for OS adjusted for treatment crossover, and for PFS (Fig. 2a); six studies for ORR as data were not reported in CEREBEL (Fig. 2b). Various treatment network plots were generated for the safety endpoints (Online Resource 6). Six studies were linked to network plots of treatment discontinuation (due to AEs), diarrhea, neutropenia, and ALT. Five studies were linked to plots for fatigue, nausea, and vomiting, four studies were included for AEs (grade 3 and above) and AST, and two studies were linked for serious AEs (Online Resource 6).

### Model selection

The Bayesian random-effects model was the base-case analysis, and was preferred over the fixed-effects model for all endpoints to account for heterogeneity among the included studies. Between-study variance cannot be estimated owing to the small number of available studies and assuming homogeneity was considered to be implausible. Hence, informative priors based on the best empirical evidence were used instead [42]. Convergence statistics for the random-effects model are shown in Online Resource 8, and convergence plots are presented in Online Resource 10. For completeness, results obtained with the fixed-effects model are shown in Online Resource 9.

### Overall survival

**Fig. 3:**
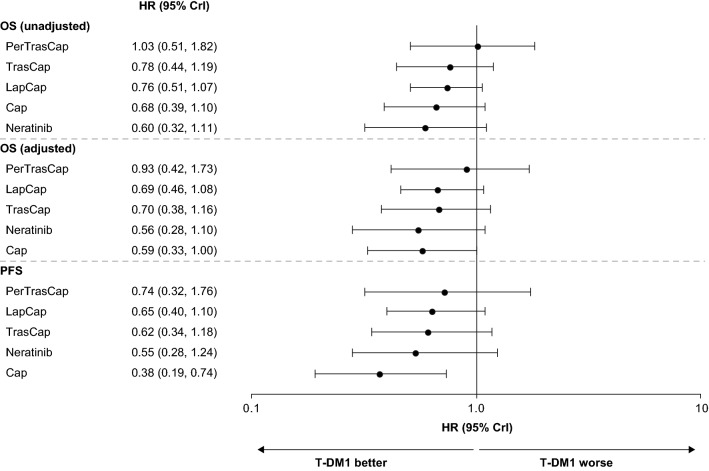
Comparative effectiveness of T-DM1 on OS, adjusted OS, and PFS. Comparators are shown in order of SUCRA ranking, with treatments ranking highest after T-DM1 at the top of the plot. *Cap* capecitabine, *CrI* credible interval, *HR* hazard ratio, *Lap* lapatinib, *OS* overall survival, *Per* pertuzumab, *PFS* progression-free survival, *SUCRA* surface under cumulative ranking curve, *T-DM1* trastuzumab emtansine, *Tras* trastuzumab

The HR data for cross-comparison of treatments are summarized for OS (the primary analysis) and for OS adjusted for treatment crossover (sensitivity analysis) in Table 2 and Fig. 3.

**Table 2:** Cross tabulation of treatment HR (95% CrI) for OS, OSX, and PFS

Drug	T-DM1	Neratinib	Cap	LapCap	TrasCap	PerTrasCap
T-DM1		OS: 0.60 (0.32, 1.14)	OS: 0.68 (0.39, 1.10)	OS: 0.76 (0.51, 1.07)	OS: 0.78 (0.44, 1.19)	OS: 1.03 (0.51, 1.82)
		OSX: 0.56 (0.28, 1.10)	OSX: 0.59 (0.33, 1.00)	OSX: 0.69 (0.46, 1.08)	OSX: 0.70 (0.38, 1.16)	OSX: 0.93 (0.42, 1.73)
		PFS: 0.55 (0.28, 1.24)	PFS: 0.38 (0.19, 0.74)	PFS: 0.65 (0.40, 1.10)	PFS: 0.62 (0.34, 1.18)	PFS: 0.74 (0.32, 1.76)
Neratinib	OS: 1.65 (0.88, 3.11)		OS: 1.13 (0.58, 2.09)	OS: 1.25 (0.76, 2.13)	OS: 1.30 (0.67, 2.35)	OS: 1.70 (0.79, 3.54)
	OSX: 1.80 (0.91, 3.57)		OSX: 1.05 (0.52, 2.01)	OSX: 1.25 (0.72, 2.10)	OSX: 1.26 (0.65, 2.24)	OSX: 1.66 (0.76, 3.29)
	PFS: 1.82 (0.81, 3.58)		PFS: 0.69 (0.33, 1.34)	PFS: 1.18 (0.65, 2.01)	PFS: 1.12 (0.55, 2.10)	PFS: 1.33 (0.56, 2.92)
Cap	OS: 1.47 (0.91, 2.54)	OS: 0.88 (0.48, 1.72)		OS: 1.11 (0.79, 1.63)	OS: 1.15 (0.76, 1.61)	OS: 1.51 (0.82, 2.59)
	OSX: 1.70 (1.00, 3.04)	OSX: 0.95 (0.50, 1.93)		OSX: 1.18 (0.85, 1.69)	OSX: 1.19 (0.79, 1.68)	OSX: 1.57 (0.84, 2.66)
	PFS: 2.62 (1.35, 5.19)	PFS: 1.45 (0.75, 2.99)		PFS: 1.71 (1.11, 2.72)	PFS: 1.62 (1.02, 2.56)	PFS: 1.94 (0.96, 3.81)
LapCap	OS: 1.32 (0.93, 1.98)	OS: 0.80 (0.47, 1.32)	OS: 0.90 (0.61, 1.26)		OS: 1.04 (0.73, 1.37)	OS: 1.36 (0.77, 2.19)
	OSX: 1.45 (0.93, 2.19)	OSX: 0.80 (0.48, 1.39)	OSX: 0.85 (0.59, 1.18)		OSX: 1.01 (0.68, 1.35)	OSX: 1.34 (0.74, 2.18)
	PFS: 1.54 (0.91, 2.52)	PFS: 0.85 (0.50, 1.54)	PFS: 0.59 (0.37, 0.90)		PFS: 0.95 (0.66, 1.30)	PFS: 1.13 (0.57, 2.17)
TrasCap	OS: 1.28 (0.84, 2.27)	OS: 0.77 (0.42, 1.49)	OS: 0.87 (0.62, 1.32)	OS: 0.96 (0.73, 1.37)		OS: 1.31 (0.85, 2.01)
	OSX: 1.42 (0.86, 2.60)	OSX: 0.80 (0.45, 1.55)	OSX: 0.84 (0.59, 1.27)	OSX: 0.99 (0.74, 1.47)		OSX: 1.32 (0.85, 2.02)
	PFS: 1.62 (0.85, 2.97)	PFS: 0.89 (0.48, 1.83)	PFS: 0.62 (0.39, 0.98)	PFS: 1.05 (0.77, 1.53)		PFS: 1.20 (0.71, 1.94)
PerTrasCap	OS: 0.97 (0.55, 1.96)	OS: 0.59 (0.28, 1.26)	OS: 0.66 (0.39, 1.22)	OS: 0.74 (0.46, 1.30)	OS: 0.76 (0.50, 1.17)	
	OSX: 1.07 (0.58, 2.37)	OSX: 0.60 (0.30, 1.32)	OSX: 0.64 (0.38, 1.19)	OSX: 0.75 (0.46, 1.36)	OSX: 0.76 (0.50, 1.18)	
	PFS: 1.34 (0.57, 3.09)	PFS: 0.75 (0.34, 1.78)	PFS: 0.52 (0.26, 1.04)	PFS: 0.88 (0.46, 1.77)	PFS: 0.83 (0.52, 1.41)	

HR < 1 indicates a better outcome with the drug in column 1 than with the comparator drug (columns 2–7)

*Cap* capecitabine, *CrI* credible interval, *HR* hazard ratio, *Lap* lapatinib, *OS* overall survival, *OSX* OS adjusted for crossover, *Per* pertuzumab, *PFS* progression-free survival, *T-DM1* trastuzumab emtansine, *Tras* trastuzumab

#### Primary analysis

T-DM1 was associated with a trend towards greater OS benefit than all other approved treatments, although the wide CrIs reflect uncertainty around the comparisons. Greater OS benefit with T-DM1 was also demonstrated by the SUCRA ranking (first), compared with other approved treatments: (1) T-DM1, (2) pertuzumab plus trastuzumab plus capecitabine (unapproved combination), (3) trastuzumab plus capecitabine, (4) lapatinib plus capecitabine, (5) capecitabine, and (6) neratinib.

#### Sensitivity analysis

In the sensitivity analysis, adjusted estimates of OS were available for both EMILIA and EGF100151 (Online Resource 5) [10, 36]. For EMILIA, the treatment crossover-adjusted HR for OS was 0.69 (95% confidence interval [CI] 0.59, 0.82) using RPSFTM. In EGF100151, the adjusted HR for OS in which treatment crossover was used as a time-dependent covariate was 0.80 (95% CI 0.64, 0.99). Intention-to-treat estimates of OS were used for the other five studies, as in the primary analysis [17, 32, 38–40]. Sensitivity analysis results were generally similar to the base-case analysis, with a numerically greater OS benefit for T-DM1 than for the other treatments (Table 2 and Fig. 3).

### Progression-free survival

Cross-comparison, between-treatment HRs for PFS are also summarized in Table 2 and Fig. 3. The analysis indicated that the likelihood of PFS benefit was greater with T-DM1 than with any of the other comparator treatments. The SUCRA ranking was also greater for T-DM1 (first) than for the other approved treatments: (1) T-DM1, (2) pertuzumab plus trastuzumab plus capecitabine, (3) lapatinib plus capecitabine, (4) trastuzumab plus capecitabine, (5) neratinib, and (6) capecitabine.

### Overall response rates

**Fig. 4:**
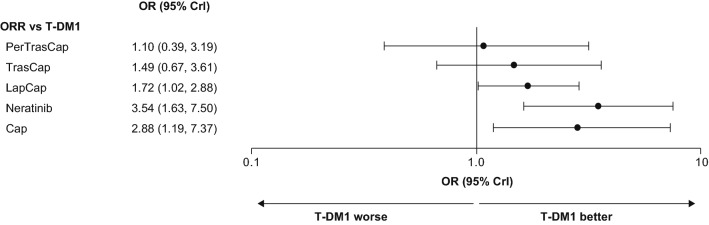
Comparative effectiveness of T-DM1 on ORR. Comparators are shown in order of SUCRA ranking, with treatments ranking highest after T-DM1 at the top of the plot. *Cap* capecitabine, *CrI* credible interval, *Lap* lapatinib, *OR* odds ratio, *ORR* overall response rate, *Per* pertuzumab, *SUCRA* surface under cumulative ranking curve, *T-DM1* trastuzumab emtansine, *Tras* trastuzumab

Comparisons of ORR with T-DM1 and with other treatments showed that T-DM1 was associated with a more favorable ORR than all comparator treatments, and was more efficacious than capecitabine, lapatinib plus capecitabine, and neratinib (Fig. 4). Consistent with this finding, the SUCRA ranking was greatest for T-DM1 compared with the other approved treatments: (1) T-DM1, (2) pertuzumab plus trastuzumab plus capecitabine, (3) trastuzumab plus capecitabine, (4) lapatinib plus capecitabine, (5) neratinib, and (6) capecitabine.

### Adverse events (grade 3 and above)

**Fig. 5:**
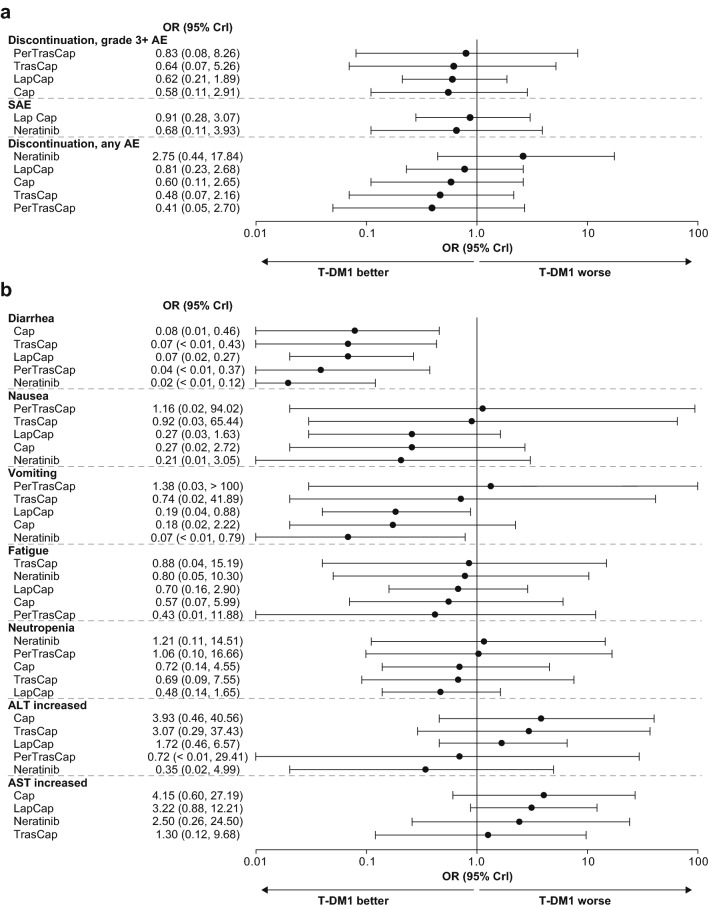
Comparative effectiveness of T-DM1 on AEs: **a** SAEs and AEs leading to discontinuation; **b** AEs by preferred term. Comparators are shown in order of SUCRA ranking, with treatments ranking highest at the top of the plot. T-DM1 ranks: discontinuation (grade 3+), SAEs, diarrhea, nausea, fatigue, first; vomiting, second; neutropenia, third; increased ALT, fourth; increased AST, fifth. *AE* adverse event, *ALT* alanine aminotransferase, *AST* aspartate aminotransferase, *Cap* capecitabine, *CrI* credible interval, *Lap* lapatinib, *OR* odds ratio, *Per* pertuzumab, *SAE* serious adverse event, *SUCRA* surface under cumulative ranking curve, *T-DM1* trastuzumab emtansine, *Tras* trastuzumab

The ORs for the likelihood of various AEs occurring with T-DM1 compared with the different comparator treatments are summarized in Fig. 5. Treatment discontinuation due to an AE of grade 3 and above was less likely with T-DM1 than with other treatments that could be compared (there was no link between neratinib and T-DM1 in the network, and these therapies could not be compared), and discontinuation due to any AE was less likely with T-DM1 than with all other treatments except for neratinib. The SUCRA rankings for discontinuation due to an AE of grade 3 and above for approved treatments were: (1) T-DM1, (2) pertuzumab plus trastuzumab plus capecitabine, (3) trastuzumab plus capecitabine, (4) lapatinib plus capecitabine, and (5) capecitabine. The likelihood of serious AEs was lower with T-DM1 than with neratinib, or lapatinib plus capecitabine; no comparison with other treatments was possible (Fig. 5a).

The ORs indicated a substantially lower risk of diarrhea associated with T-DM1 than with other treatments, and this difference was reflected by the SUCRA rankings: (1) T-DM1, (2) capecitabine, (3) trastuzumab plus capecitabine, (4) lapatinib plus capecitabine, (5) pertuzumab plus trastuzumab plus capecitabine, and (6) neratinib. Most ORs for fatigue, nausea, vomiting, and neutropenia favored T-DM1 over other treatments; however, there was higher uncertainty for lower risk of vomiting with T-DM1 than with neratinib, or lapatinib plus capecitabine (Fig. 5b).

ORs indicated that increased AST was more likely with T-DM1 than with other treatments, and was least likely with capecitabine. The SUCRA rankings were: (1) capecitabine, (2) lapatinib plus capecitabine, (3) neratinib, (4) trastuzumab plus capecitabine, and (5) T-DM1. The risk of increased ALT with T-DM1 was higher than with trastuzumab plus capecitabine, lapatinib plus capecitabine, or capecitabine.

Effect size could not be quantified for mucosal inflammation. T-DM1 was consistently better than the comparators, but no mucosal inflammation events were reported in one of the cohorts in study EGF100151; removal of this study disconnected the network, preventing further analysis. Similarly, the absence of events in one arm of study GBG 26 prevented further analysis of thrombocytopenia; T-DM1 was consistently worse than the comparators but this could not be quantified, even when GBG 26 was removed from the network. T-DM1 was also consistently worse than comparators in terms of anemia events. The absence of such AEs in one arm of GBG 26 afforded the possibility to re-analyze with that study excluded; OR estimates for T-DM1 are shown in Online Resource 7. Finally, no effect size could be estimated for PPE, owing to PPE being a rare event in EMILIA, thereby preventing estimation of an OR.

## Discussion

This SR and NMA of RCTs undertaken in previously treated patients with unresectable, HER2-positive LABC or mBC represents the first synthesis of clinical effectiveness and safety of studies in mBC. The results demonstrated that safety and efficacy outcomes with T-DM1 were, in general, more favorable than those with other treatments that are currently available/approved in this indication (pertuzumab plus trastuzumab plus capecitabine is not approved). This is particularly encouraging because T-DM1 is the first-in-class antibody–drug conjugate therapy approved in this indication.

In terms of efficacy, T-DM1 was associated with greater OS benefit than all approved comparators, both based on data at the end of the randomized phase of the EMILIA trial and after adjustment for pre-specified treatment crossover. T-DM1 was also associated with better PFS and ORR than other treatments.

The random-effects model results had wide CrIs, reflecting uncertainty around the comparisons. Fixed-effects results (Online Resource 9) which assume no between-study heterogeneity had similar hazard ratios but narrower CrIs.

In terms of safety outcomes, there is high uncertainty due to the small number of studies included, and the fact that not all endpoints were available for all comparator treatments. Discontinuation due to an AE or to an AE of at least grade 3 severity was less likely with T-DM1 than with other treatments, with the exception of neratinib, and T-DM1 generally had a more tolerable safety profile in terms of gastroenterological side effects than the other treatments, being substantially less likely to be associated with diarrhea. Similar point estimates for nausea and vomiting were seen with T-DM1 and trastuzumab plus capecitabine. Fatigue was generally less likely with T-DM1 than with comparators. Hematological effects were more difficult to quantify in this analysis. The likelihood of neutropenia with T-DM1 was slightly higher than with neratinib, and lower than with other approved treatments. When comparisons could be made, T-DM1 was more likely to be associated with anemia and thrombocytopenia than were other treatments. Finally, T-DM1 was among the treatments most likely to be associated with hepatic side effects.

Cardiac safety is an important consideration for patients treated with HER2 inhibitors, including T-DM1, who are at increased risk of developing left ventricular dysfunction. A decrease of left ventricular ejection fraction has been observed in patients treated with T-DM1. For example, in EMILIA, left ventricular dysfunction occurred in 1.8% of patients in the T-DM1-treated group and 3.3% of patients in the lapitinib plus capecitabine group.

Generally, the risk of bias in different aspects of the RCTs included in the NMA was low. Response to treatment in GBG 26 (trastuzumab plus capecitabine versus capecitabine) [32] was at risk of being assessed by unblinded investigators due to a lack of central assessment; and CEREBEL (lapitinib plus capecitabine versus trastuzumab plus capecitabine) [17] was underpowered because it stopped before recruitment was complete, following interim analysis and a recommendation from its data monitoring committee. In several studies, risk of bias was unclear due to a lack of published information. This was typically related to details of randomization in open-label studies and to the methods of allocation concealment used, but little information about patients’ baseline similarity was available in one study [39]. Differences in the follow-up times of the studies included in the NMA did not affect PFS and OS, which were analyzed using HRs that inherently assume proportional hazards. However, results for ORR and safety endpoints may have been affected. EMILIA was the largest study and had the longest follow-up, a fact that was reflected in the increased numbers of events observed in this study. Nonetheless, the efficacy and safety of T-DM1 were generally more favorable compared with the other treatments analyzed. It was not possible to analyze the rates of safety events to account for differences in the follow-up times, because not all studies reported the appropriate data to conduct such an analysis.

A key benefit of the current analysis is its reproducibility, which enables updates to be performed when additional evidence becomes available. A significant limitation of the NMA presented here is that a formal analysis of the likelihood of thrombocytopenia with T-DM1 versus other treatments could not be performed (i.e., effect size was not quantifiable due to thrombocytopenia being a rare event in most comparators). Thrombocytopenia was the most frequently reported grade 3 or above AE in patients treated with T-DM1 in the EMILIA trial, affecting 14% of patients, compared with < 1% of patients in the lapatinib plus capecitabine group [13].

## Conclusion

T-DM1 was associated with a greater OS, PFS, and ORR benefit than lapatinib plus capecitabine, trastuzumab plus capecitabine, capecitabine monotherapy, and neratinib monotherapy in patients with previously treated HER2-positive LABC or mBC. The improvements in OS with T-DM1 were seen in the analyses of both ITT (unadjusted) and after adjustment for treatment crossover. In the safety analyses, T-DM1 was associated with a greater benefit than the other treatments for the majority of endpoints with evaluable results with the exception of thrombocytopenia and hepatic AEs.

## Electronic supplementary material

Below is the link to the electronic supplementary material.

Supplementary file1 (PDF 644 kb)

Supplementary file2 (PDF 369 kb)

Supplementary file3 (PDF 359 kb)

Supplementary file4 (PDF 343 kb)

Supplementary file5 (PDF 389 kb)

Supplementary file6 (PDF 268 kb)

Supplementary file7 (PDF 260 kb)

Supplementary file8 (PDF 370 kb)

Supplementary file9 (PDF 462 kb)

Supplementary file10 (PDF 27936 kb)

Supplementary file11 (PDF 142 kb)
