# Supplementary material for: Evaluating the clinical effectiveness and safety of various HER2-targeted regimens after prior taxane/trastuzumab in patients with previously treated, unresectable, or metastatic HER2-positive breast cancer: a systematic review and network meta-analysis
Source: Breast Cancer Res Treat. 2020 Feb 25;180(3):597–609. doi: 10.1007/s10549-020-05577-7 (PMC7103014; doi:10.1007/s10549-020-05577-7)
Supplement: Supplementary file 1 — Supplementary file1 (PDF 644 kb) [file 10549_2020_5577_MOESM1_ESM.pdf]

SUPPLEMENTARY APPENDICES

Evaluating the clinical effectiveness and safety of various HER2-targeted regimens after prior taxane/trastuzumab in patients with previously treated, unresectable, or metastatic HER2-positive breast cancer: a systematic review and network meta-analysis

Authors:

Noman Paracha, Adriana Reyes, Véronique Diéras, Ian Krop, Xavier Pivot, Ander Urruticoechea

Corresponding author:

Noman Paracha

F. Hoffmann-La Roche AG
Grenzacherstrasse 124
4070 Basel
Switzerland

Tel: +41 61 688 2661

Email: noman.paracha@roche.com

Online Resource 1: Appendix 1. Search strings

A. Initial systematic review searches (1 January 1998–2 July 2013)

1. Embase.com search strategy for Embase® and MEDLINE® (searched on 2 July 2013)

#	Search term	Results
1.	'clinical trial'/exp	957 718
2.	'randomization'/de	61 391
3.	'controlled study'/de	4 065 662
4.	'comparative study'/de	4 065 662
5.	'single blind procedure'/de	17 053
6.	'double blind procedure'/de	17 053
7.	'crossover procedure'/de	36 469
8.	'placebo'/de	234 281
9.	'clinical trial' OR 'clinical trials'	1 185 783
10.	'controlled clinical trial' OR 'controlled clinical trials'	495 189
11.	'randomised controlled trial' OR 'randomized controlled trial' OR 'randomised controlled trials' OR 'randomized controlled trials'	401 142
12.	'randomisation' OR 'randomization' OR random*	944 333
13.	Rct	14 060
14.	'random allocation'	1380
15.	'randomly allocated'	19 152
16.	'allocated randomly'	1894
17.	allocated NEAR/2 random OR assign* NEAR/2 random*	79 938
18.	(single OR double OR triple OR treble) NEAR/1 (blind* OR mask*)	201 187
19.	placebo*	314 029
20.	'prospective study'/de	228 455
21.	#1 OR #2 OR #3 OR #4 OR #5 OR #6 OR #7 OR #8 OR #9 OR #10 OR #11 OR #12 OR #13 OR #14 OR #15 OR #16 OR #17 OR #18 OR #19 OR #20	5 291 436
22.	'case study'/de	22 040
23.	'case report'	1974 751
24.	'abstract report'/de	89 579
25.	'letter'/de	794 885
26.	#22 OR #23 OR #24 OR #25	2 712 617
27.	#21 NOT #26	5 165 646

#	Search term	Results
28.	nrct OR ‘n rct’ OR n?rct	76
29.	'controlled clinical trial'/exp	449 047
30.	‘Prospective study’/exp	228 455
31.	‘Major clinical study’/exp	2 092 727
32.	‘Intervention study’/exp	16 566
33.	(clinical NEXT/1 trial*):ti,ab	260 600
34.	#27 OR #28 OR #29 OR #30 OR #31 OR #32 OR #33	6 348 471
35.	'chemotherapy'/exp OR chemotherap* OR (chemo* OR biologic* OR hormon* OR endocrin* OR target*) NEAR/2 (therap* OR treat*)	1 933 734
36.	'antineoplastic agent'/syn OR ‘antineoplastic drug’ OR ‘anticancer drug’ OR ‘tumor inhibitor’ OR ‘anticarcinogenic agents’ OR ‘anticarcinogenic agents’	1 388 659
37.	'biological therapy'/syn OR ‘biologic therapy’ OR ‘tissue therapy’	1 053 654
38.	'systemic therapy'/syn	19 709
39.	'molecularly targeted therapy'/syn	7634
40.	'hormonal therapy'/syn OR ‘endocrine therapy’ OR ‘endocrine treatment’	180 112
41.	'angiogenesis inhibitor'/syn	70 860
42.	‘aromatase inhibitor’/syn	19 800
43.	((her2 OR 'her 2' OR 'her 2' OR angiogen*) NEAR/4 inhibit*) OR antiangiogen* OR 'anti angiogen' OR ('her 2' OR her2 OR erbb2 OR 'erbb 2') NEXT/2 antibody	87 113
44.	androgen* OR 'anti estrogen' OR 'anti oestrogen'	182 702
45.	'anthracycline'/syn OR anthracycline*	170 906
46.	'gemcitabine'/syn OR gemcite OR gemzar OR ‘ly 188011’ OR ly188011	30 103
47.	'sunitinib'/syn OR ‘pha 2909040ad’ OR pha2909040ad OR ‘su 010398’ OR ‘su 011248’ OR ‘su 10398’ OR ‘su 11248’ OR su010398 OR su011248 OR su10398 OR su11248 OR sutent	11 257
48.	'bevacizumab'/syn OR avastin OR ‘nsc 704865’ OR nsc704865	27 303
49.	'trastuzumab'/syn OR herceptin	20 458
50.	'lapatinib'/syn OR ‘gw 2016’ OR ‘gw 572016’ OR ‘gw 572016f’ OR gw2016 OR gw572016 OR gw572016f OR tykerb OR tyverb	6003
51.	'pertuzumab'/syn OR 2C4 OR ‘monoclonal antibody 2C4’ OR omnitarg OR ‘r 1273’ OR r1273 OR ‘rhumab 2C4’	8063
52.	'neratinib'/syn OR hki 272 OR hki272 OR ‘way 177820’ OR way177820	379
53.	'ertumaxomab'/syn OR rexomun	85
54.	'dasatinib'/syn OR ‘bms 354825’ OR bms354825 OR sprycel	5887

#	Search term	Results
55.	'cyclophosphamide'/syn OR alkyroxan OR ‘b 518’ OR b518 OR carloxan OR ciclofosfamida OR ciclolen OR cicloxal OR clafen OR cyclo-cell OR cycloblastin OR cyclofos amide OR cyclofosfamid OR cyclophar OR cyclophosphamid OR cyclophosphan OR cyclostin OR cycloxan OR cyphos OR cytophosphan OR cytoxan OR ‘endocyclo phosphate’ OR endoxan OR endoxon-asta OR enduxan OR genoxal OR ledoxan OR ledoxina OR mitoxan OR neosan OR neosar OR noristan OR ‘nsc 26271’ OR ‘nsc 2671’ OR procytox OR procytoxide OR semdoxan OR sendoxan OR syklofosfamid	23 447
56.	'methotrexate'/syn OR ‘methopterine’ OR abitrexate OR amethopterin OR ametopterine OR antifolan OR biotrexate OR canceren OR ‘cl 14377’ OR cl14377 OR emtexate OR emthexat OR emtrexate OR enthexate OR farmitrexat OR farmitrexate OR farmotrex OR folex OR ifamet OR lantarel OR ledertrexate OR maxtrex OR metex OR methoblastin OR methohexate OR methotrate OR methotrexat OR methotrexato OR methoxtrexate OR methrotrexate OR methylaminopterin OR methylaminopterine OR meticil OR metothrexate OR metotrexat OR metotrexate OR metotrexin OR metrex OR mexate OR mexate-aq OR ‘mpi 5004’ OR mpi5004 OR MTX OR neotrexate OR novatrex OR nsc 740 OR nsc740 OR reumatrex OR rheumatrex OR rheumatrex dose pack OR texate OR texorate OR trexall OR xaken OR zexate OR MTX	12 389
57.	'fluorouracil'/syn OR ‘5 fluoruracil’ OR 5 fu OR accusite OR actino-hermal OR adrucil OR carac OR effluderm OR efudex OR efudix OR efurix OR f6627 OR fivoflu OR ‘fluoro uracil’ OR fluoroblastin OR fluoroplex OR ‘fluorouracil 5’ OR fluoruracil OR fluouracil OR fluoxan OR fluracedyl OR fluracil OR fluracilium OR fluril OR ‘fluro uracil’ OR fluroblastin OR ifacil OR ‘nsc 18913’ OR ‘nsc 19893’ OR nsc18913 OR nsc19893 OR oncofu OR ‘ro 2-9757’ OR ‘ro 2 9757’ OR ‘ro2-9757’ OR ‘ro2 9757’ OR uflahex OR utoral OR verrumal	91344
58.	‘cisplatin'/syn OR abiplatin OR biocisplatinum OR biocysplatinum OR blastolem OR briplatin OR platinum OR ‘cddp ti’ OR cis-platinum OR ‘cis ddp’ OR cis diamine dichloroplatinum OR cis diaminechloroplatinum OR ‘cis platinous diamino dichloride’ OR ‘cis platinum’ OR cytoplatin OR cytosplat OR docistin OR elvecis OR kemoplat OR lederplatin OR mpi 5010 OR mpi5010 OR neoplatin OR niyaplat OR nk 801 OR noveldexis OR nsc 119875 OR platamine OR platamine rtu OR platiblastin OR platidiam OR platimine OR platinex OR platinil OR platinol OR platinol-aq OR platinol aq OR platinoxan OR platiran OR platistil OR platistin OR platosin OR randa OR romcis OR sicatem OR ‘spi 077’ OR tecnoplatin	970

#	Search term	Results
59.	'carboplatin'/syn OR blastocarb OR boplatex OR carboplat OR carbosin OR carbotec OR carplan OR CBDCA OR erbakar OR ercar OR ifacap OR jm-8 OR ‘jm 8’ OR kemocarb OR ‘nsc 241240’ OR oncocarbin OR paraplatin OR paraplatin OR paraplatine	41 860
60.	'platinum'/syn	42 773
61.	'oxaliplatin'/syn OR crisapla OR dacotin OR dacplat OR eloxatin OR eloxatine OR heloxatin OR oplat OR oxalip OR oxaltic OR transplastin OR xaliplat	18 713
62.	'capecitabine'/syn OR apecitab OR ‘ro 09-1978’ OR ‘ro 09 1978’ OR ‘ro 091978’ OR ro09-1978 OR ‘ro09 1978’ OR ro091978 OR xeloda	14 965
63.	'vinorelbine'/syn OR navelbin OR vinbine OR vinelbine OR navelbine OR vinorelbine	12 531
64.	'epirubicin'/syn OR epiadriamycin OR epidoxorubicin OR binarin OR ellence OR epi-cell OR epiadriamycin OR epidoxo OR epidx OR epifil OR epilem OR ‘farmorrubicina rtu’ OR farmorubicin OR ‘imi 28’ OR ‘nsc 256942’ OR pharmorubicin OR pidorubicin	20 728
65.	'doxorubicin'/syn OR ‘14 hydroxydaunomycin’ OR ‘14 hydroxydaunorubicin’ OR a.d.mycin OR adriablastin OR adriacin OR adriamicin OR adriamycin OR adriblastin OR adrim OR adrubicin OR amminac OR caelix OR caelyx OR caelyx/doxil OR carcinocin OR dexorubicin OR ‘dox sl’ OR doxil OR doxolem OR ‘doxor lyo’ OR doxorubin OR evacet OR farmiblastina OR ‘fi 106’ OR fi106 OR ifadox OR lipodox OR myocet OR ‘nsc 123127’ OR nsc123127 OR rastocin OR resmycin OR ‘rp 25253’ OR rp25253 OR rubex OR rubidox OR sarcodoxome OR ‘tlc d 99’	134 303
66.	'paclitaxel'/syn OR ‘abi 007’ OR abi007 OR abraxane OR anzatax OR asotax OR biotax OR ‘bms 181339’ OR bms181339 OR bristaxol OR britaxol OR coroxane OR formoxol OR genexol OR hunxol OR ifaxol OR intaxel OR medixel OR mitotax OR ‘nsc 125973’ OR nsc125973 OR onxol OR pacitaxel OR pacxel OR padexol OR parexel OR paxceed OR paxene OR paxus OR praxel OR taxocris OR taxol OR ‘taxus (drug)’ OR taycovit OR yewtaxan	64 091
67.	'docetaxel'/syn OR daxotel OR dexotel OR docefrez OR ‘lit 976’ OR lit976 OR ‘nsc 628503’ OR nsc628503 OR oncodocel OR ‘rp 56976’ OR rp56976 OR taxoter OR texot	31 258
68.	'ixabepilone'/syn OR ‘azaepothilone B’ OR ‘bms 247550’ OR ‘bms 247550-1’ OR ‘bms 247550 1’ OR bms247550 OR bms247550-1 OR ‘bms247550 1’ OR ixempra OR ‘nsc 710428’ OR nsc710428	1280

#	Search term	Results
69.	'abraxane'/syn OR ‘abi 007’ OR abi007 OR abraxane OR anzatax OR asotax OR biotax OR ‘bms 181339’ OR bms181339 OR bristaxol OR britaxol OR coroxane OR formoxol OR genexol OR hunxol OR ifaxol OR intaxel OR medixel OR mitotax OR ‘nsc 125973’ OR nsc125973 OR onxol OR pacitaxel OR pacxel OR padexol OR parexel OR paxceed OR paxene OR paxus OR praxel OR taxocris OR taxol OR ‘taxus (drug)’ OR taycovit OR yewtaxan	64 091
70.	'tamoxifen'/syn OR kessar OR ‘nsc 180973’ OR tamoplac OR tamoxasta	47 189
71.	'toremifene'/syn OR estrimex OR fareston OR ‘fc 1157 a’ OR ‘fc 1157a’ OR fc1157a	5760
72.	'fulvestrant'/syn OR faslodex OR ‘ici 182 780’ OR ‘ici 182, 780’ OR ‘ici 182780’ OR ici182780 OR ‘zd 182780’ OR ‘zd 9238’ OR zd182780 OR zd9238 OR ‘zm 182780’ OR zm182780	1769
73.	'anastrazole'/syn OR arimidex OR ‘ici d1033’ OR icid1033 OR trozolet OR ‘zd 1033’ OR zd1033	6308
74.	'goserelin'/syn OR ‘buserelin carbazamide’ OR ‘ici 118 630’ OR ‘ici 118630’ OR ici118630 OR prozoladex OR zoladex	5560
75.	'letrozole'/syn OR ‘cgs 20267’ OR cgs20267 OR femar OR femara	6552
76.	'exemestane'/syn OR aromasin OR aromasine OR ‘fce 24304’ OR fce24304 OR nikidess OR ‘pnu 155971’ OR pnu155971	3583
77.	'medroxyprogesterone acetate'/syn OR acetoxymethylprogesterone OR amen OR aragest OR clinofem OR clinovir OR curretab OR cycrin OR depo-prodasone OR depo-provera OR depo-subqprovera OR ‘depo prodasone’ OR ‘depo provera’ OR ‘depo subQ provera’ OR depoclinovir OR depomedroxyprogesterone OR depoprodasone OR depopromone OR depoprovera OR estrofarlutal OR farkital OR farlutal OR gestapolar OR gestapuran OR gestapuron OR ‘hysron h’ OR lutopolar OR ‘lutoral farmit’ OR manodepa OR ‘medioxyprogesterone acetate’ OR ‘medroxy progesterone acetate’ OR medroxyprogesteronacetate OR ‘medroxyprogesterone 17-acetate’ OR ‘medroxyprogesterone 17 acetate’ OR meges OR megestron OR meprate OR methylacetoxyprogesterone OR methylpregnone OR ‘mpa gyn’ 5 OR ‘nsc 26 386’ OR ‘nsc 26386’ OR nsc26386 OR oragest OR perkitex OR perlutex OR prodafem OR prodasone OR progen OR progevera OR prothyra OR provera OR ralovera OR repromap OR veramix OR veraplex	13 537
78.	'megestrol'/syn OR megestrole	5622
79.	'navelbine'/syn OR navelbin OR vinbine OR vinelbine OR navelbine OR vinorelbine	12 531

#	Search term	Results
80.	‘leuprorelin’/syn OR ‘a 43818’ OR a43818 OR ‘abbott 43818’ OR carcinil OR ‘depo lupron’ OR eligard OR enanton OR ginecrin OR leuplin OR leuprogel OR leuprolid OR leupron OR ‘lorelin depot’ OR lucrin OR lupride OR luprolex OR lupron OR ‘procren depot’ OR procrin OR prostap OR reliser OR ‘tap 144’ OR tap144 OR tapros OR trenantone OR viadur	8419
81.	'taxane derivatives' OR taxan*	14 673
82.	'trastuzumab emtansine'/syn OR tdm1 OR 'tdm 1' OR tdm?1 OR 't dm 1' OR 'trastuzumab emtansine'	408
83.	#35 OR #36 OR #37 OR #38 OR #39 OR #40 OR #41 OR #42 OR #43 OR #44 OR #45 OR #46 OR #47 OR #48 OR #49 OR #50 OR #51 OR #52 OR #53 OR #54 OR #55 OR #56 OR #57 OR #58 OR #59 OR #60 OR #61 OR #62 OR #63 OR #64 OR #65 OR #66 OR #67 OR #68 OR #69 OR #70 OR #71 OR #72 OR #73 OR #74 OR #75 OR #76 OR #77 OR #78 OR #79 OR #80 OR #81 OR #82	2 959 746
84.	'breast tumor'/exp OR 'breast tumour' OR 'breast tumor'	336 971
85.	'breast'/exp OR 'breast'	509 279
86.	'breast neoplasms'/exp OR 'breast neoplasm' OR breast NEAR/5 carcinoma OR breast NEAR/5 cancer OR breast NEAR/5 malignan*	370 273
87.	#84 OR #85 OR #86	509 279
88.	advanced OR metastat* OR refract* OR recurren* OR salva* OR 'late' NEXT/2 'stage' OR resistan* OR 'stage iii' OR (stage AND iii*) OR 'stage iv' OR 'stage 3' OR 'stage 4' OR 'breast metastasis'/exp OR 'metastasis'/exp OR 'recurrent disease'/exp	2 643 597
89.	#87 AND #88	153 358
90.	'epidermal growth factor receptor 2'/syn OR her2 OR 'her 2' OR 'her-2'	34 982
91.	second* OR progress* OR relapse* OR recurren* OR fail* OR resistance OR pretreated OR 'pre treated' OR 'pre-treated' OR refract* OR previous* OR salvage OR prior OR 'second-line' OR 'second line'	6 140 182
92.	#34 AND #83 AND #89 AND #90 AND #91	5759
**93.**	**#92 AND ([article]/lim OR [article in press]/lim OR [erratum]/lim) AND [20-12-2013]/sd**	306

2. Cochrane search strategy (searched on 2 July 2013)

#	Search term	Results
1.	MeSH descriptor Breast Neoplasms explode all trees	7856
2.	MeSH descriptor Breast explode all trees	569

#	Search term	Results
3.	breast	19 877
4.	(#1 OR #2 OR #3)	19 886
5.	MeSH descriptor Neoplasm Metastasis explode all trees	3508
6.	(advanced OR metastat* OR refract* OR recurren* OR salva* OR (late adj stage) OR resistan* OR "stage III" OR (stage AND III*) OR "stage IV" OR "stage 3" OR "stage 4" OR "stage IIIC" OR "stage IIIB" OR unresect*)	95 429
7.	MeSH descriptor Neoplasm Recurrence, Local explode all trees	3110
8.	(#5 OR #6 OR #7)	96 384
9.	MeSH descriptor Receptor, Epidermal Growth Factor explode all trees	328
10.	MeSH descriptor Receptor, erbB-2 explode all trees	347
11.	HER2 OR HER-2 OR "HER 2" OR "HER positive" OR HER-positive OR HER-overexpressing OR "HER overexpressing" OR ErbB2 OR Erb-B2 OR "Human epidermal growth factor receptor 2" OR "cerbB 2" OR Her2neu	708
12.	(#9 OR #10 OR #11)	1029
13.	Second-line OR "Second line" OR Secondary OR Progress* OR Relapse* OR Recurren* OR Fail* OR Resist* OR Pretreated OR "pre treated" OR "pre-treated" OR Refract* OR Salvage OR Prior OR Previous*	201 911
14.	MeSH descriptor Antineoplastic Protocols explode all trees	10 195
15.	MeSH descriptor Drug Therapy explode all trees	109 541
16.	(Biologic* OR chemo* OR systemic OR target*) NEAR/2 (therap* OR treat*)	14 425
17.	"antineoplastic agent" OR antineoplastic OR antitumor OR anticancer OR "tumor inhibitor" OR "anti tumor" OR "anti tumour" OR antitumour	19 604
18.	"angiogenesis inhibitor" OR "neovascularization inhibitor" OR "monoclonal antibody"	1638
19.	"her2 inhibitor" OR "her 2 inhibitor" OR "angiogen inhibitor" OR "her2 antibody" OR "her 2 antibody" OR "ErbB2 inhibitor" OR "ErbB2 antibody"	9
20.	MeSH descriptor Anthracyclines explode all trees	3840
21.	anthracyclin	31
22.	MeSH descriptor Cyclophosphamide explode all trees	3771
23.	MeSH descriptor Methotrexate explode all trees	2673
24.	MeSH descriptor Fluorouracil explode all trees	3674
25.	MeSH descriptor Cisplatin explode all trees	3131
26.	MeSH descriptor Carboplatin explode all trees	917
27.	MeSH descriptor Platinum explode all trees	90

#	Search term	Results
28.	MeSH descriptor Epirubicin explode all trees	745
29.	MeSH descriptor Doxorubicin explode all trees	3238
30.	MeSH descriptor Paclitaxel explode all trees	1308
31.	MeSH descriptor Tamoxifen explode all trees	1777
32.	MeSH descriptor Toremifene explode all trees	63
33.	MeSH descriptor Goserelin explode all trees	355
34.	MeSH descriptor Medroxyprogesterone Acetate explode all trees	798
35.	MeSH descriptor Megestrol explode all trees	237
36.	MeSH descriptor Leuprolide explode all trees	448
37.	gemcitabine OR gemcite OR gemzar OR "ly 188011" OR ly188011	1379
38.	sunitinib OR "pha 2909040ad" OR pha2909040ad OR "su 010398" OR "su 011248" OR "su 10398" OR "su 11248" OR su010398 OR su011248 OR su10398 OR su11248 OR sutent	140
39.	bevacizumab OR avastin OR "nsc 704865" OR nsc704865	764
40.	trastuzumab OR herceptin OR "aromatase inhibitor" OR aomat* near/2 (inhibit*)	733
41.	lapatinib OR "gw 2016" OR "gw 572016" OR "gw 572016f" OR gw2016 OR gw572016 OR gw572016f OR tykerb OR tyverb	128
42.	pertuzumab OR 2C4 OR "monoclonal antibody 2C4" OR omnitarg OR "r 1273" OR r1273 OR "rhumab 2C4"	55
43.	neratinib OR hki 272 OR hki272 OR "way 177820" OR way177820	4
44.	ertumaxomab OR rexomun	0
45.	dasatinib OR "bms 354825" OR bms354825 OR sprycel	81
46.	cyclophosphamide OR alkyroxan OR "b 518" OR b518 OR carloxan OR ciclofosfamida OR ciclolen OR cicloxal OR clafen OR cyclo-cell OR cycloblastin OR cyclofos amide OR cyclofosfamid OR cyclophar OR cyclophosphamid OR cyclophosphan OR cyclostin OR cycloxan OR cyphos OR cytophosphan OR cytoxan OR "endocyclo phosphate" OR endoxan OR endoxon-asta OR enduxan OR genoxal OR ledoxan OR ledoxina OR mitoxan OR neosan OR neosar OR noristan OR "nsc 26271" OR "nsc 2671" OR procytox OR procytoxide OR semdoxan OR sendoxan OR syklofosfamid	6849

#	Search term	Results
47.	methotrexate OR "methopterine" OR abitrexate OR amethopterin OR ametopterine OR antifolan OR biotrexate OR canceren OR "cl 14377" OR cl14377 OR emtexate OR emthexat OR emtrexate OR enthexate OR farmitrexat OR farmitrexate OR farmotrex OR folex OR ifamet OR lantarel OR ledertrexate OR maxtrex OR metex OR methoblastin OR methohexate OR methotrate OR methotrexat OR methotrexato OR methoxtrexate OR methrotrexate OR methylaminopterin OR methylaminopterine OR meticil OR metothrexate OR metotrexat OR metotrexate OR metotrexin OR metrex OR mexate OR mexate-aq OR "mpi 5004" OR mpi5004 OR MTX OR neotrexate OR novatrex OR nsc 740 OR nsc740 OR reumatrex OR rheumatrex OR rheumatrex dose pack OR texate OR texorate OR trexall OR xaken OR zexate OR MTX	5058
48.	fluorouracil OR "5 fluoruracil" OR 5 fu OR accusite OR actino-hermal OR adrucil OR carac OR effluderm OR efudex OR efudix OR efurix OR f6627 OR fivoflu OR "fluoro uracil" OR fluoroblastin OR fluoroplex OR "fluorouracil 5" OR fluoruracil OR fluouracil OR fluoxan OR fluracedyl OR fluracil OR fluracilium OR fluril OR "fluro uracil" OR fluroblastin OR ifacil OR "nsc 18913" OR "nsc 19893" OR nsc18913 OR nsc19893 OR oncofu OR "ro 2-9757" OR "ro 2 9757" OR "ro2-9757" OR "ro2 9757" OR uflahex OR utoral OR verrumal	7682
49.	cisplatin OR abiplatin OR biocisplatinum OR biocysplatinum OR blastolem OR briplatin OR platinum OR "cddp ti" OR cis-platinum OR "cis ddp" OR cis diamine dichloroplatinum OR cis diaminechloroplatinum OR "cis platinous diamino dichloride" OR "cis platinum" OR cytoplatin OR cytosplat OR docistin OR elvecis OR kemoplat OR lederplatin OR mpi 5010 OR mpi5010 OR neoplatin OR niyaplat OR nk 801 OR noveldexis OR nsc 119875 OR platamine OR platamine rtu OR platiblastin OR platidiam OR platimine OR platinex OR platinil OR platinol OR platinol-aq OR platinol aq OR platinoxan OR platiran OR platistil OR platistin OR platosin OR randa OR romcis OR sicatem OR "spi 077" OR tecnoplatin	7449
50.	carboplatin OR blastocarb OR boplatex OR carboplat OR carbosin OR carbotec OR carplan OR CBDCA OR erbakar OR ercar OR ifacap OR jm-8 OR "jm 8" OR kemocarb OR "nsc 241240" OR oncocarbin OR paraplatin OR paraplatin OR paraplatine	2399
51.	platinum	1620
52.	oxaliplatin OR crisapla OR dacotin OR dacplat OR eloxatin OR eloxatine OR heloxatin OR oplat OR oxalip OR oxaltic OR transplastin OR xaliplat	706
53.	capecitabine OR apecitab OR "ro 09-1978" OR "ro 09 1978" OR "ro 091978" OR ro09-1978 OR "ro09 1978" OR ro091978 OR xeloda	613

#	Search term	Results
54.	vinorelbine OR navelbin OR vinbine OR vinelbine OR navelbine OR vinorelbine	769
55.	epirubicin OR epiadriamycin OR epidoxorubicin OR binarin OR ellence OR epi-cell OR epiadriamycin OR epidoxo OR epidx OR epifil OR epilem OR "farmorrubicina rtu" OR farmorubicin OR "imi 28" OR "nsc 256942" OR pharmorubicin OR pidorubicin	1743
56.	doxorubicin OR "14 hydroxydaunomycin" OR "14 hydroxydaunorubicin" OR a.d.mycin OR adriablastin OR adriacin OR adriamicin OR adriamycin OR adriblastin OR adrim OR adrubicin OR amminac OR caelix OR caelyx OR doxil OR carcinocin OR dexorubicin OR "dox sl" OR doxil OR doxolem OR "doxor lyo" OR doxorubin OR evacet OR farmiblastina OR "fi 106" OR fi106 OR ifadox OR lipodox OR myocet OR "nsc 123127" OR nsc123127 OR rastocin OR resmycin OR "rp 25253" OR rp25253 OR rubex OR rubidox OR sarcodoxome OR "tlc d 99"	5150
57.	paclitaxel OR "abi 007" OR abi007 OR abraxane OR anzatax OR asotax OR biotax OR "bms 181339" OR bms181339 OR bristaxol OR britaxol OR coroxane OR formoxol OR genexol OR hunxol OR ifaxol OR intaxel OR medixel OR mitotax OR "nsc 125973" OR nsc125973 OR onxol OR pacitaxel OR pacxel OR padexol OR parexel OR paxceed OR paxene OR paxus OR praxel OR taxocris OR taxol OR "taxus (drug)" OR taycovit OR yewtaxan	2809
58.	docetaxel OR daxotel OR dexotel OR docefrez OR "lit 976" OR lit976 OR "nsc 628503" OR nsc628503 OR oncodocel OR "rp 56976" OR rp56976 OR taxoter OR texot	1554
59.	ixabepilone OR "azaepothilone B" OR "bms 247550" OR "bms 247550-1" OR "bms 247550 1" OR bms247550 OR bms247550-1 OR "bms247550 1" OR ixempra OR "nsc 710428" OR nsc710428	31
60.	abraxane OR "abi 007" OR abi007 OR abraxane OR anzatax OR asotax OR biotax OR "bms 181339" OR bms181339 OR bristaxol OR britaxol OR coroxane OR formoxol OR genexol OR hunxol OR ifaxol OR intaxel OR medixel OR mitotax OR "nsc 125973" OR nsc125973 OR onxol OR pacitaxel OR pacxel OR padexol OR parexel OR paxceed OR paxene OR paxus OR praxel OR taxocris OR taxol OR "taxus (drug)" OR taycovit OR yewtaxan	382
61.	tamoxifen OR kessar OR "nsc 180973" OR tamoplac OR tamoxasta	3156
62.	toremifene OR estrimex OR fareston OR "fc 1157 a" OR "fc 1157a" OR fc1157a	117

#	Search term	Results
63.	fulvestrant OR faslodex OR "ici 182 780" OR "ici 182, 780" OR "ici 182780" OR ici182780 OR "zd 182780" OR "zd 9238" OR zd182780 OR zd9238 OR "zm 182780" OR zm182780	97
64.	anastrazole OR arimidex OR "ici d1033" OR icid1033 OR trozolet OR "zd 1033" OR zd1033	180
65.	goserelin OR "buserelin carbazamide" OR "ici 118 630" OR "ici 118630" OR ici118630 OR prozoladex OR zoladex	649
66.	letrozole OR "cgs 20267" OR cgs20267 OR femar OR femara	490
67.	exemestane OR aromasin OR aromasine OR "fce 24304" OR fce24304 OR nikidess OR "pnu 155971" OR pnu155971	214
68.	"medroxyprogesterone acetate" OR acetoxymethylprogesterone OR amen OR aragest OR clinofem OR clinovir OR curretab OR cycrin OR depo-prodasone OR depo-provera OR depo-subqprovera OR "depo prodasone" OR "depo provera" OR "depo subQ provera" OR depoclinovir OR depomedroxyprogesterone OR depoprodasone OR depopromone OR depoprovera OR estrofarlutal OR farkital OR farlutal OR gestapolar OR gestapuran OR gestapuron OR "hysron h" OR lutopolar OR "lutoral farmit" OR manodepa OR "medioxyprogesterone acetate" OR "medroxy progesterone acetate" OR medroxyprogesteronacetate OR "medroxyprogesterone 17-acetate" OR "medroxyprogesterone 17 acetate" OR meges OR megestron OR meprate OR methylacetoxyprogesterone OR methylpregnone OR "mpa gyn" 5 OR "nsc 26 386" OR "nsc 26386" OR nsc26386 OR oragest OR perkitex OR perlutex OR prodafem OR prodasone OR progen OR progevera OR prothyra OR provera OR ralovera OR repromap OR veramix OR veraplex	1540
69.	megestrol OR megestrole	444
70.	navelbine OR navelbin OR vinbine OR vinelbine OR navelbine OR vinorelbine	769
71.	leuprorelin OR "a 43818" OR a43818 OR "abbott 43818" OR carcinil OR "depo lupron" OR eligard OR enanton OR ginecrin OR leuplin OR leuprogel OR leuprolid OR leupron OR "lorelin depot" OR lucrin OR lupride OR luprolex OR lupron OR "procren depot" OR procrin OR prostap OR reliser OR "tap 144" OR tap144 OR tapros OR trenantone OR viadur	220
72.	“taxane derivatives” OR taxan*	519
73.	"trastuzumab emtansine" OR tdm1 OR "tdm 1" OR tdm?1 OR "t dm 1" OR "trastuzumab emtansine"	4
74.	MeSH descriptor Aromatase Inhibitors explode all trees	379

#	Search term	Results
75.	(#14 OR #15 OR #16 OR #17 OR #18 OR #19 OR #20 OR #21 OR #22 OR #23 OR #24 OR #25 OR #26 OR #27 OR #28 OR #29 OR #30 OR #31 OR #32 OR #33 OR #34 OR #35 OR #36 OR #37 OR #38 OR #39 OR #40 OR #41 OR #42 OR #43 OR #44 OR #45 OR #46 OR #47 OR #48 OR #49 OR #50 OR #51 OR #52 OR #53 OR #54 OR #55 OR #56 OR #57 OR #58 OR #59 OR #60 OR #61 OR #62 OR #63 OR #64 OR #65 OR #66 OR #67 OR #68 OR #69 OR #70 OR #71 OR #72 OR #73 OR #74)	136 918
76.	(#4 AND #8 AND #12 AND #13 AND #75), from 2012 to 2013 limited to Cochrane Reviews (Reviews and Protocols) and Other Reviews	9
77.	(#4 AND #8 AND #12 AND #13 AND #75), from 2012 to 2013 limited to CENTRAL and Method studies	32

3. MEDLINE® In-Process (Pubmed) search strategy (searched on 2 July 2013)

#	Search term	Results
1	Breast Neoplasms[tiab] OR Breast cancer[tiab] OR Breast cancers[tiab] OR Breast neoplasm[tiab] OR Breast neoplasms[tiab] OR Breast tumour[tiab] OR Breast tumor[tiab] OR Breast tumors[tiab] OR Mammary carcinoma[tiab] OR Mammary carcinomas[tiab] OR Mammary neoplasm[tiab] OR Mammary neoplasms[tiab] OR Breast tumours[tiab]	175 343
2	HER2 OR HER-2 OR HER-positive OR HER -overexpressing OR ErbB2 OR Erb-B2 OR Human epidermal growth factor receptor 2 OR cerbB 2 OR Her2neu OR Her2/neu	24 091
3	Advanced OR Metastatic OR Stage 3 OR Stage 4 OR Stage III OR Stage IIIB OR Stage IIIC OR Stage IV OR Metastasis OR Metastases OR Unresectable OR Inoperable	1 296 406
4	#1 AND #2 AND #3	6231
5	Second-line OR Second line OR Secondary[tiab] OR Progression[tiab] OR Progressed[tiab] OR Progressive[tiab] OR Relapse*[tiab] OR Recurren*[tiab] OR Failed[tiab] OR Failure[tiab] OR Resistant[tiab] OR Resistance[tiab] OR Pretreated[tiab] OR Refractory[tiab] OR Previously[tiab] OR Salvage[tiab] OR Prior[tiab] OR Previous*[tiab] OR Previous[tiab]	3 675 069

#	Search term	Results
6	gemcitabine OR gemcite OR gemzar OR "ly 188011" OR ly188011 OR sunitinib OR "pha 2909040ad" OR pha2909040ad OR "su 010398" OR "su 011248" OR "su 10398" OR "su 11248" OR su010398 OR su011248 OR su10398 OR su11248 OR sutent OR bevacizumab OR avastin OR trastuzumab OR herceptin OR lapatinib OR "gw 2016" OR "gw 572016" OR "gw 572016f" OR gw2016 OR gw572016 OR gw572016f OR tykerb OR tyverb OR pertuzumab OR 2C4 OR "monoclonal antibody 2C4" OR omnitarg OR "r 1273" OR r1273 OR "rhumab 2C4" OR neratinib OR hki 272 OR hki272 OR "way 177820" OR way177820 OR ertumaxomab OR rexomun OR dasatinib OR "bms 354825" OR bms354825 OR sprycel	2 358 309
7	cyclophosphamide OR alkyroxan OR "b 518" OR b518 OR carloxan OR ciclofosfamida OR ciclolen OR cicloxal OR clafen OR cyclo-cell OR cycloblastin OR cyclofos amide OR cyclofosfamid OR cyclophar OR cyclophosphamid OR cyclophosphan OR cyclostin OR cycloxan OR cyphos OR cytophosphan OR cytoxan OR "endocyclo phosphate" OR endoxan OR endoxon-asta OR enduxan OR genoxal OR ledoxan OR ledoxina OR mitoxan OR neosan OR neosar OR noristan OR "nsc 26271" OR "nsc 2671" OR procytox OR procytoxide OR semdoxan OR sendoxan OR syklofosfamid OR methotrexate OR "methopterine" OR abitrexate OR amethopterin OR ametopterine OR antifolan OR biotrexate OR canceren OR "cl 14377" OR cl14377 OR emtexate OR emthexat OR emtrexate OR enthexate OR farmitrexat OR farmitrexate OR farmotrex OR folex OR ifamet OR lantarel OR ledertrexate OR maxtrex OR metex OR methoblastin OR methohexate OR methotrate OR methotrexat OR methotrexato OR methoxtrexate OR methrotrexate OR methylaminopterin OR methylaminopterine OR meticil OR metothrexate OR metotrexat OR metotrexate OR metotrexin OR metrex OR mexate OR mexate-aq OR "mpi 5004" OR mpi5004 OR MTX OR neotrexate OR novatrex OR nsc 740 OR nsc740 OR reumatrex OR rheumatrex OR rheumatrex dose pack OR texate OR texorate OR trexall OR xaken OR zexate OR MTX	364 337

#	Search term	Results
8	fluorouracil OR "5 fluoruracil" OR 5 fu OR accusite OR actino-hermal OR adrucil OR carac OR effluderm OR efudex OR efudix OR efurix OR f6627 OR fivoflu OR "fluoro uracil" OR fluoroblastin OR fluoroplex OR "fluorouracil 5" OR fluoruracil OR fluouracil OR fluoxan OR fluracedyl OR fluracil OR fluracilium OR fluril OR "fluro uracil" OR fluroblastin OR ifacil OR "nsc 18913" OR "nsc 19893" OR nsc18913 OR nsc19893 OR oncofu OR "ro 2-9757" OR "ro 2 9757" OR "ro2-9757" OR "ro2 9757" OR uflahex OR utoral OR verrumal OR cisplatin OR abiplatin OR biocisplatinum OR biocysplatinum OR blastolem OR briplatin OR platinum OR "cddp ti" OR cis-platinum OR "cis ddp" OR cis diamine dichloroplatinum OR cis diaminechloroplatinum OR "cis platinous diamino dichloride" OR "cis platinum" OR cytoplatin OR cytosplat OR docistin OR elvecis OR kemoplat OR lederplatin OR mpi 5010 OR mpi5010 OR neoplatin OR niyaplat OR nk 801 OR noveldexis OR nsc 119875 OR platamine OR platamine rtu OR platiblastin OR platidiam OR platimine OR platinex OR platinil OR platinol OR platinol-aq OR platinol aq OR platinoxan OR platiran OR platistil OR platistin OR platosin OR randa OR romcis OR sicatem OR "spi 077" OR tecnoplatin OR carboplatin OR blastocarb OR boplatex OR carboplat OR carbosin OR carbotec OR carplan OR CBDCA OR erbakar OR ercar OR ifacap OR jm-8 OR "jm 8" OR kemocarb OR "nsc 241240" OR oncocarbin OR paraplatin OR paraplatin OR paraplatine OR platinum	113 918
9	oxaliplatin OR crisapla OR dacotin OR dacplat OR eloxatin OR eloxatine OR heloxatin OR oplat OR oxalip OR oxaltic OR transplastin OR xaliplat	5545
10	capecitabine OR apecitab OR "ro 09-1978" OR "ro 09 1978" OR "ro 091978" OR ro09-1978 OR "ro09 1978" OR ro091978 OR xeloda	3609
11	vinorelbine OR navelbin OR vinbine OR vinelbine OR navelbine OR vinorelbine	3201
12	epirubicin OR epiadriamycin OR epidoxorubicin OR binarin OR ellence OR epi-cell OR epiadriamycin OR epidoxo OR epidx OR epifil OR epilem OR "farmorrubicina rtu" OR farmorubicin OR "imi 28" OR "nsc 256942" OR pharmorubicin OR pidorubicin	7841
13	doxorubicin OR "14 hydroxydaunomycin" OR "14 hydroxydaunorubicin" OR a.d.mycin OR adriablastin OR adriacin OR adriamicin OR adriamycin OR adriblastin OR adrim OR adrubicin OR amminac OR caelix OR caelyx OR caelyx/doxil OR carcinocin OR dexorubicin OR "dox sl" OR doxil OR doxolem OR "doxor lyo" OR doxorubin OR evacet OR farmiblastina OR "fi 106" OR fi106 OR ifadox OR lipodox OR myocet OR "nsc 123127" OR nsc123127 OR rastocin OR resmycin OR "rp 25253" OR rp25253 OR rubex OR rubidox OR sarcodoxome OR "tlc d 99"	53 803

#	Search term	Results
14	paclitaxel OR "abi 007" OR abi007 OR abraxane OR anzatax OR asotax OR biotax OR "bms 181339" OR bms181339 OR bristaxol OR britaxol OR coroxane OR formoxol OR genexol OR hunxol OR ifaxol OR intaxel OR medixel OR mitotax OR "nsc 125973" OR nsc125973 OR onxol OR pacitaxel OR pacxel OR padexol OR parexel OR paxceed OR paxene OR paxus OR praxel OR taxocris OR taxol OR "taxus (drug)" OR taycovit OR yewtaxan OR docetaxel OR daxotel OR dexotel OR docefrez OR "lit 976" OR lit976 OR "nsc 628503" OR nsc628503 OR oncodocel OR "rp 56976" OR rp56976 OR taxoter OR texot OR ixabepilone OR "azaepothilone B" OR "bms 247550" OR "bms 247550-1" OR "bms 247550 1" OR bms247550 OR bms247550-1 OR "bms247550 1" OR ixempra OR "nsc 710428" OR nsc710428 OR abraxane OR "abi 007" OR abi007 OR abraxane OR anzatax OR asotax OR biotax OR "bms 181339" OR bms181339 OR bristaxol OR britaxol OR coroxane OR formoxol OR genexol OR hunxol OR ifaxol OR intaxel OR medixel OR mitotax OR "nsc 125973" OR nsc125973 OR onxol OR pacitaxel OR pacxel OR padexol OR parexel OR paxceed OR paxene OR paxus OR praxel OR taxocris OR taxol OR "taxus (drug)" OR taycovit OR yewtaxan OR tamoxifen OR kessar OR "nsc 180973" OR tamoplac OR tamoxasta OR toremifene OR estrimex OR fareston OR "fc 1157 a" OR "fc 1157a" OR fc1157a	1 266 673
15	fulvestrant OR faslodex OR "ici 182 780" OR "ici 182, 780" OR "ici 182780" OR ici182780 OR "zd 182780" OR "zd 9238" OR zd182780 OR zd9238 OR "zm 182780" OR zm182780 OR anastrazole OR arimidex OR "ici d1033" OR icid1033 OR trozolet OR "zd 1033" OR zd1033 OR goserelin OR "buserelin carbazamide" OR "ici 118 630" OR "ici 118630" OR ici118630 OR prozoladex OR zoladex OR letrozole OR "cgs 20267" OR cgs20267 OR femar OR femara OR exemestane OR aromasin OR aromasine OR "fce 24304" OR fce24304 OR nikidess OR "pnu 155971" OR pnu155971	9485

#	Search term	Results
16	"medroxyprogesterone acetate" OR acetoxymethylprogesterone OR amen OR aragest OR clinofem OR clinovir OR curretab OR cycrin OR depo-prodasone OR depo-provera OR depo-subqprovera OR "depo prodasone" OR "depo provera" OR "depo subQ provera" OR depoclinovir OR depomedroxyprogesterone OR depoprodasone OR depopromone OR depoprovera OR estrofarlutal OR farkital OR farlutal OR gestapolar OR gestapuran OR gestapuron OR "hysron h" OR lutopolar OR "lutoral farmit" OR manodepa OR "medioxyprogesterone acetate" OR "medroxy progesterone acetate" OR medroxyprogesteronacetate OR "medroxyprogesterone 17-acetate" OR "medroxyprogesterone 17 acetate" OR meges OR megestron OR meprate OR methylacetoxyprogesterone OR methylpregnone OR "mpa gyn" 5 OR "nsc 26 386" OR "nsc 26386" OR nsc26386 OR oragest OR perkitex OR perlutex OR prodafem OR prodasone OR progen OR progevera OR prothyra OR provera OR ralovera OR repromap OR veramix OR veraplex	7004
17	megestrol OR megestrole OR navelbine OR navelbin OR vinbine OR vinelbine OR navelbine OR vinorelbine	5079
18	leuprorelin OR "a 43818" OR a43818 OR "abbott 43818" OR carcinil OR "depo lupron" OR eligard OR enanton OR ginecrin OR leuplin OR leuprogel OR leuprolid OR leupron OR "lorelin depot" OR lucrin OR lupride OR luprolex OR lupron OR "procren depot" OR procrin OR prostap OR reliser OR "tap 144" OR tap144 OR tapros OR trenantone OR viadur OR "taxane derivatives" OR taxan* OR "trastuzumab emtansine" OR tdm1 OR "tdm 1" OR tdm?1 OR "t dm 1" OR "trastuzumab emtansine"	17 649
19	"antineoplastic agent" OR antineoplastic OR antitumor OR anticancer OR "tumor inhibitor" OR (biologic* OR hormon* OR endocrine OR target*) AND (therap* OR treatment) OR "angiogenesis inhibitor" OR "neovascularization inhibitor" OR "monoclonal antibody" OR her2 inhibitor OR her 2 inhibitor OR her-2 inhibitor OR angiogen inhibitor OR her2 antibody OR her 2 antibody OR her-2 antibody OR ErbB2 inhibitor OR ErbB2 inhibitor OR ErbB2 inhibitor OR angiogen inhibitor OR ErbB2 antibody OR ErbB2 antibody OR ErbB2 antibody OR Erb-B2 inhibitor OR Erb-B2 inhibitor OR Erb-B2 inhibitor OR angiogen inhibitor OR Erb-B2 antibody OR Erb-B2 antibody OR Erb-B2 antibody	1 639 127

#	Search term	Results
20	androgen OR "anti estrogen" OR "anti oestrogen" OR "hormonal therapy" OR "endocrine therapy" OR "endocrine treatment" OR endocrinotherapy OR "hormone therapy" OR "hormone treatment" OR "molecularly targeted therapy" OR "molecular target therapy" OR "molecular targeted therapy" OR "targeted cancer therapy" OR "targeted molecular therapy" OR "targeted therapy" OR "systemic therapy" OR "systemic treatment" OR "biological therapy" OR "biologic therapies" OR "biologic therapy" OR "biological therapies" OR biotherapies OR organotherapy OR "tissue therapy" OR "anti cancer drug" OR "anti neoplastic agent" OR antineoplastic OR "anticancer agent" OR "anticancer drug" OR anticancerogen OR anticarcinogen OR antitumor OR "cancer inhibitor" OR "carcinostatic drug" OR "target therapy" OR "aromatase inhibitor" OR "aromatase inhibitors"	1 399 219
21	#6 OR #7 OR #8 OR #9 OR #10 OR #11 OR #12 OR #13 OR #14 OR #15 OR #16 OR #17 OR #18 OR #19 OR #20	5 481 323
22	#4 AND #5 AND #21	3023
23	#22 AND (in process[sb] OR pubstatusaheadofprint)	197

B. First systematic review update (1 October 2012 to 30 June 2016)

In order to replicate the searches performed in the initial systematic review in December 2012, which were executed in embase.com (medline plus Embase), PubMed (Medline in process) and Cochrane library (on Cochrane website), the same search strings were used to perform 3 searches using the Ovid search platform.

1. Medline and Embase (searched on 30 June 2016)

#	Search term	Results
1	exp clinical trial/	1 845 882
2	randomization/	158628
3	controlled study/	4 967 297
4	'comparative study'/	715 918
5	single blind procedure/	22 380
6	'double blind procedure'/	131 975
7	'crossover procedure'/	47 729

8	'placebo'/	290 151
9	(clinical trial' or 'clinical trials').mp.	2 091 341
10	(controlled clinical trial or controlled clinical trials).mp.	531 878
11	('randomised controlled trial' or 'randomized controlled trial' or 'randomised controlled trials' or 'randomized controlled trials').mp.	1 114 423
12	('randomisation' or 'randomization' or random*).mp.	2 337 154
13	RCT.mp.	34 886
14	'random allocation'.mp.	90 519
15	'randomly allocated'.mp.	46 285
16	'allocated randomly'.mp.	4072
17	((allocated adj2 random) or (assign* adj2 random*)).mp.	194 834
18	((single or double or triple or treble) adj (blind* or mask*)).mp.	440 786
19	placebo*.mp.	572 040
20	'prospective study'/	340 196
21	or/1-20	8 679 707
22	'case study'/	39 048
23	'case report'.mp.	2 385 421
24	'abstract report'/	89 677
25	'letter'/	893 897
26	or/22-25	3 221 577
27	21 not 26	8 518 667
28	(nrct or n rct or n?rct).mp.	197
29	exp 'controlled clinical trial'/	552 197
30	exp Prospective study/	762 758
31	exp Major clinical study/	2 504 990
32	exp Intervention study/	774 213
33	(clinical adj trial*).ti,ab.	624 863
34	or/27-33	10 152 776

35	exp 'chemotherapy'/ or chemotherap*.mp. or ((chemo* or biologic* or hormon* or endocrin* or target*) adj2 (therap* or treat*)).mp.	1 553 931
36	antineoplastic agent/ or (antineoplastic drug or anticancer drug or tumor inhibitor or anticarcinogenic agents or anticarcinogenic agents).mp.	277 127
37	'biological therapy'/ or (biologic therapy or tissue therapy).mp.	15 007
38	'systemic therapy'/	17 489
39	'molecularly targeted therapy'/	15 273
40	'hormonal therapy'/ or (endocrine therapy or endocrine treatment).mp.	44 391
41	'angiogenesis inhibitor'/	15 398
42	aromatase inhibitor/	16 741
43	(((her2 or 'her 2' or 'her 2' or angiogen*) adj4 inhibit*) or ((antiangiogen* or anti angiogen or her 2 or her2 or erbb2 or erbb 2) adj2 antibody)).mp.	64 564
44	(androgen* or anti estrogen or anti oestrogen).mp.	194 978
45	anthracycline/ or anthracycline*.mp.	43 637
46	gemcitabine'/ or (gemcite or gemzar or ly 188011 or ly188011).mp.	41 517
47	'sunitinib'/ or (pha 2909040ad or pha2909040ad or "su 010398" or "su 011248" or su 10398 or su 11248 or su010398 or su011248 or su10398 or su11248 or sutent).mp.	16 832
48	bevacizumab/ or (avastin or nsc 704865 or nsc704865).mp.	49 425
49	trastuzumab/ or herceptin.mp.	33 970
50	'lapatinib'/ or (gw 2016 or gw 572016 or gw 572016f or gw2016 or gw572016 or gw572016f or tykerb or tyverb).mp.	9216
51	'pertuzumab'/ or (2C4 or monoclonal antibody 2C4 or omnitarg or r 1273 or r1273 or rhumab 2C4).mp.	2842
52	'neratinib'/ or (hki 272 or hki272 or way 177820 or way177820).mp.	1041
53	'ertumaxomab'/ or rexomun.mp.	104
54	'dasatinib'/ or (bms 354825 or bms354825 or sprycel).mp.	9363
55	'cyclophosphamide'/ or (alkyroxan or b 518 or b518 or carloxan or ciclofosfamida or ciclolen or cicloxal or clafen or cyclo-cell or cycloblastin or cyclofos amide or cyclofosfamid or cyclophar or cyclophosphamid or cyclophosphan or cyclostin or cycloxan or cyphos	187 947

	or cytophosphan or cytoxan or endocyclo phosphate or endoxan or endoxon-asta or enduxan or genoxal or ledoxan or ledoxina or mitoxan or neosan or neosar or noristan or nsc 26271 or nsc 2671 or procytox or procytoxide or semdoxan or sendoxan or syklofosfamid).mp.	
56	'methotrexate'/ or (methopterine or abitrexate or amethopterin or ametopterine or antifolan or biotrexate or canceren or cl 14377 or cl14377 or emtexate or emthexat or emtrexate or enthexate or farmitrexat or farmitrexate or farmotrex or folex or ifamet or lantarel or ledertrexate or maxtrex or metex or methoblastin or methohexate or methotrate or methotrexat or methotrexato or methoxtrexate or methrotrexate or methylaminopterin or methylaminopterine or meticil or metothrexate or metotrexat or metotrexate or metotrexin or metrex or mexate or mexate-aq or mpi 5004 or mpi5004 or MTX or neotrexate or novatrex or nsc 740 or nsc740 or reumatrex or rheumatrex or rheumatrex dose pack or texate or texorate or trexall or xaken or zexate or MTX).mp.	160 720
57	fluorouracil/ or (5 fluoruracil or 5 fu or accusite or actino-hermal or adrucil or carac or effluderm or efudex or efudix or efurix or f6627 or fivoflu or fluoro uracil or fluoroblastin or fluoroplex or fluorouracil 5 or fluoruracil or fluouracil or fluoxan or fluracedyl or fluracil or fluracilium or fluril or fluro uracil or fluroblastin or ifacil or nsc 18913 or nsc 19893 or nsc18913 or nsc19893 or oncofu or ro 2-9757 or ro 2 9757 or ro2-9757 or ro2 9757 or uflahex or utoral or verrumal).mp.	162 261
58	cisplatin/ or (abiplatin or biocisplatinum or biocysplatinum or blastolem or briplatin or platinum or cddp ti or cis-platinum or cis ddp or cis diamine dichloroplatinum or cis diaminechloroplatinum or cis platinous diamino dichloride or cis platinum or cytoplatin or cytosplat or docistin or elvecis or kemoplat or lederplatin or mpi 5010 or mpi5010 or neoplatin or niyaplat or nk 801 or noveldexis or nsc 119875 or platamine or platamine rtu or platiblastin or platidiam or platimine or platinex or platinil or platinol or platinol-aq or platinol aq or platinoxan or platiran or platistil or platistin or platosin or randa or romcis or sicatem or "spi 077" or tecnoplatin).mp.	254 983
59	carboplatin/ or (blastocarb or boplatex or carboplat or carbosin or carbotec or carplan or CBDCA or erbakar or ercar or ifacap or jm-8 or jm 8 or kemocarb or nsc 241240 or oncocarbin or paraplatin or paraplatin or paraplatine).mp.	62 773

60	platinum/	31 008
61	oxaliplatin/ or (crisapla or dacotin or dacplat or eloxatin or eloxatine or heloxatin or oplat or oxalip or oxaltic or transplastin or xaliplat).mp.	27 149
62	capecitabine/ or (apecitab or ro 09-1978 or "ro 09 1978" or "ro 091978" or ro09-1978 or ro09 1978 or ro091978 or xeloda).mp.	24 520
63	vinorelbine/ or (navelbin or vinbine or vinelbine or navelbine or vinorelbine).mp.	19 082
64	epirubicin/ or (epiadriamycin or epidoxorubicin or binarin or ellence or epi-cell or epiadriamycin or epidoxo or epidx or epifil or epilem or farmorrubicina rtu or farmorubicin or imi 28 or nsc 256942 or pharmorubicin or pidorubicin).mp.	29 083
65	doxorubicin/ or (14 hydroxydaunomycin or 14 hydroxydaunorubicin or ad mycin or adriablastin or adriacin or adriamicin or adriamycin or adriblastin or adrim or adrubicin or amminac or caelix or caelyx or caelyx/doxil or carcinocin or dexorubicin or dox sl or doxil or doxolem or doxor lyo or doxorubin or evacet or farmiblastina or fi 106 or fi106 or ifadox or lipodox or myocet or nsc 123127 or nsc123127 or rastocin or resmycin or rp 25253 or rp25253 or rubex or rubidox or sarcodoxome or tlc d 99).mp.	205 099
66	paclitaxel/ or ("abi 007" or abi007 or abraxane or anzatax or asotax or biotax or bms 181339 or bms181339 or bristaxol or britaxol or coroxane or formoxol or genexol or hunxol or ifaxol or intaxel or medixel or mitotax or nsc 125973 or nsc125973 or onxol or pacitaxel or pacxel or padexol or parexel or paxceed or paxene or paxus or praxel or taxocris or taxol or taxus or taycovit or yewtaxan).mp.	108 893
67	docetaxel/ or (daxotel or dexotel or docefrez or lit 976 or lit976 or nsc 628503 or nsc628503 or oncodocel or rp 56976 or rp56976 or taxoter or texot).mp.	43 066
68	ixabepilone/ or (azaepothilone B or bms 247550 or bms 247550-1 or bms 247550 1 or bms247550 or bms247550-1 or bms247550 1 or ixempra or nsc 710428 or nsc710428).mp.	1647
69	abraxane/ or ("abi 007" or abi007 or abraxane or anzatax or asotax or biotax or bms 181339 or bms181339 or bristaxol or britaxol or coroxane or formoxol or genexol or hunxol or ifaxol or intaxel or medixel or mitotax or nsc 125973 or nsc125973 or onxol or pacitaxel or pacxel or	92 160

	padexol or parexel or paxceed or paxene or paxus or praxel or taxocris or taxol or taxus or taycovit or yewtaxan).mp.	
70	tamoxifen/ or (kessar or nsc 180973 or tamoplac or tamoxasta).mp.	69 566
71	toremifene/ or (estrimex or fareston or fc 1157 a or fc 1157a or fc1157a).mp.	2491
72	fulvestrant/ or (faslodex or ici 182 780 or ici 182, 780 or ici 182780 or ici182780 or zd 182780 or zd 9238 or zd182780 or zd9238 or zm 182780 or zm182780).mp.	9792
73	anastrazole/ or (arimidex or ici d1033 or icid1033 or trozolet or zd 1033 or zd1033).mp.	8104
74	goserelin/ or (buserelin carbazamide or ici 118 630 or ici 118630 or ici118630 or prozoladex or zoladex).mp.	7872
75	letrozole/ or (cgs 20267 or cgs20267 or femar or femara).mp.	8875
76	exemestane/ or (aromasin or aromasine or fce 24304 or fce24304 or nikidess or pnu 155971 or pnu155971).mp.	4888
77	medroxyprogesterone acetate/ or (acetoxymethylprogesterone or amen or aragest or clinofem or clinovir or curretab or cycrin or depo-prodasone or depo-provera or depo-subqprovera or depo prodasone or depo provera or depo subQ provera or depoclinovir or depomedroxyprogesterone or depoprodasone or depopromone or depoprovera or estrofarlutal or farkital or farlutal or gestapolar or gestapuran or gestapuron or hysron h or lutopolar or lutoral farmit or manodepa or medioxyprogesterone acetate or medroxy progesterone acetate or medroxyprogesteronacetate or medroxyprogesterone 17-acetate or medroxyprogesterone 17 acetate or meges or megestron or meprate or methylacetoxyprogesterone or methylpregnone or mpa gyn 5 or nsc 26 386 or nsc 26386 or nsc26386 or oragest or perkitex or perlutex or prodafem or prodasone or progen or progevera or prothyra or provera or ralovera or repromap or veramix or veraplex).mp.	22 072
78	megestrol/ or megestrole.mp.	2597
79	navelbine/ or (navelbin or vinbine or vinelbine or navelbine or vinorelbine).mp.	19 082
80	leuprorelin/ or (a 43818 or a43818 or abbott 43818 or carcinil or depo lupron or eligard or enanton or ginecrin or leuplin or leuprogel or leuprolid or leupron or lorelin depot or lucrin or lupride or luprolex or	12 357

	lupron or procren depot or procrin or prostap or reliser or tap 144 or tap144 or tapros or trenantone or viadur).mp.	
81	(taxane derivatives or taxan*).mp.	27 052
82	trastuzumab emtansine/ or (tdm1 or tdm 1 or tdm?1 or t dm 1 or trastuzumab emtansine).mp.	1627
83	or/35-82	2 394 207
84	exp breast tumor/ or (breast tumour or breast tumor).mp.	669 179
85	exp breast/ or breast.mp.	1 020 962
86	exp breast neoplasms/ or ((breast neoplasm or breast) adj5 carcinoma).mp. or (breast adj5 cancer).mp. or (breast adj5 malignan*).mp.	782 574
87	or/84-86	1 022 733
88	((advanced or metastat* or refract* or recurren* or salva* or late) adj2 (stage or resistan* or stage iii or (stage and iii*) or stage iv or stage 3 or stage 4)).mp. or exp breast metastasis/ or exp metastasis/ or exp recurrent disease/	897 037
89	87 and 88	134 160
90	epidermal growth factor receptor 2/ or (her2 or her 2 or her-2).mp.	77 845
91	(second* or progress* or relapse* or recurren* or fail* or resistance or pretreated or pre treated or pre-treated or refract* or previous* or salvage or prior or second-line or second line).mp.	12 897 506
92	34 and 83 and 89 and 90 and 91	4908
93	limit 92 to “dd=20121001-20160630"	2076

2. Medline (Epub Ahead of Print, In-Process & Other Non-Indexed Citations, Ovid MEDLINE® Daily and Ovid MEDLINE® 1946 to Present) (searched 30 June 2016)

#	**Search terms**	**Results**
1	(Breast Neoplasms or Breast cancer or Breast cancers or Breast neoplasm or Breast neoplasms or Breast tumour or Breast tumor or Breast tumors or Mammary carcinoma or Mammary carcinomas or Mammary neoplasm or Mammary neoplasms or Breast tumours).ti,ab.	225 602

2	(HER2 or HER-2 or HER-positive or HER -overexpressing or ErbB2 or Erb-B2 or Human epidermal growth factor receptor 2 or cerbB 2 or Her2neu).mp. or Her2/	291 46
3	(Advanced or Metastatic or Stage 3 or Stage 4 or Stage III or Stage IIIB or Stage IIIC or Stage IV or Metastasis or Metastases or Unresectable or Inoperable).mp.	748 501
4	1 and 2 and 3	7700
5	Second-line.ti,ab. or Second line,ti.ab. or Secondary.ti,ab. or Progression.ti,ab. or Progressed.ti,ab. or Progressive.ti,ab. or Relapse*.ti,ab. or Recurren*.ti,ab. or Failed.ti,ab. or Failure.ti,ab. or Resistant.ti,ab. or Resistance.ti,ab. or Pretreated.ti,ab. or Refractory.ti,ab. or Previously.ti,ab. or Salvage.ti,ab. or Prior.ti,ab. or Previous*.ti,ab. or Previous.ti,ab.	4 505 949
6	(gemcitabine or gemcite or gemzar or ly 188011 or ly188011 or sunitinib or pha 2909040ad or pha2909040ad or "su 010398" or "su 011248" or su 10398 or su 11248 or su010398 or su011248 or su10398 or su11248 or sutent or bevacizumab or avastin or trastuzumab or herceptin or lapatinib or gw 2016 or gw 572016 or gw 572016f or gw2016 or gw572016 or gw572016f or tykerb or tyverb or pertuzumab or 2C4 or monoclonal antibody 2C4 or omnitarg or r 1273 or r1273 or rhumab 2C4 or neratinib or hki 272 or hki272 or way 177820 or way177820 or ertumaxomab or rexomun or dasatinib or bms 354825 or bms354825 or sprycel).mp.	40 609
7	(cyclophosphamide or alkyroxan or b 518 or b518 or carloxan or ciclofosfamida or ciclolen or cicloxal or clafen or cyclo-cell or cycloblastin or cyclofos amide or cyclofosfamid or cyclophar or cyclophosphamid or cyclophosphan or cyclostin or cycloxan or cyphos or cytophosphan or cytoxan or endocyclo phosphate or endoxan or endoxon-asta or enduxan or genoxal or ledoxan or ledoxina or mitoxan or neosan or neosar or noristan or nsc 26271 or nsc 2671 or procytox or procytoxide or semdoxan or sendoxan or syklofosfamid or methotrexate or methopterine or abitrexate or amethopterin or ametopterine or antifolan or biotrexate or canceren or cl 14377 or cl14377 or emtexate or emthexat or emtrexate or enthexate or farmitrexat or farmitrexate or farmotrex or folex or ifamet or lantarel or ledertrexate or maxtrex or metex or methoblastin or methohexate or methotrate or methotrexat or methotrexato or methoxtrexate or methrotrexate or methylaminopterin or methylaminopterine or meticil or metothrexate or metotrexat or	103 171

	metotrexate or metotrexin or metrex or mexate or mexate-aq or mpi 5004 or mpi5004 or MTX or neotrexate or novatrex or nsc 740 or nsc740 or reumatrex or rheumatrex or rheumatrex dose pack or texate or texorate or trexall or xaken or zexate or MTX).mp.	
8	(fluorouracil or 5 fluoruracil or 5 fu or accusite or actino-hermal or adrucil or carac or effluderm or efudex or efudix or efurix or f6627 or fivoflu or fluoro uracil or fluoroblastin or fluoroplex or fluorouracil 5 or fluoruracil or fluouracil or fluoxan or fluracedyl or fluracil or fluracilium or fluril or fluro uracil or fluroblastin or ifacil or nsc 18913 or nsc 19893 or nsc18913 or nsc19893 or oncofu or ro 2-9757 or ro 2 9757 or ro2-9757 or ro2 9757 or uflahex or utoral or verrumal or cisplatin or abiplatin or biocisplatinum or biocysplatinum or blastolem or briplatin or platinum or cddp ti or cis-platinum or cis ddp or cis diamine dichloroplatinum or cis diaminechloroplatinum or cis platinous diamino dichloride or cis platinum or cytoplatin or cytosplat or docistin or elvecis or kemoplat or lederplatin or mpi 5010 or mpi5010 or neoplatin or niyaplat or nk 801 or noveldexis or nsc 119875 or platamine or platamine rtu or platiblastin or platidiam or platimine or platinex or platinil or platinol or platinol-aq or platinol aq or platinoxan or platiran or platistil or platistin or platosin or randa or romcis or sicatem or "spi 077" or tecnoplatin or carboplatin or blastocarb or boplatex or carboplat or carbosin or carbotec or carplan or CBDCA or erbakar or ercar or ifacap or jm-8 or jm 8 or kemocarb or nsc 241240 or oncocarbin or paraplatin or paraplatin or paraplatine or platinum).mp.	135 667
9	(oxaliplatin or crisapla or dacotin or dacplat or eloxatin or eloxatine or heloxatin or oplat or oxalip or oxaltic or transplastin or xaliplat).mp.	8222
10	(capecitabine or apecitab or ro 09-1978 or "ro 09 1978" or "ro 091978" or ro09-1978 or ro09 1978 or ro091978 or xeloda).mp.	5239
11	(vinorelbine or navelbin or vinbine or vinelbine or navelbine or vinorelbine).mp.	3690
12	(epirubicin or epiadriamycin or epidoxorubicin or binarin or ellence or epi-cell or epiadriamycin or epidoxo or epidx or epifil or epilem or farmorrubicina rtu or farmorubicin or imi 28 or nsc 256942 or pharmorubicin or pidorubicin).mp.	6538
13	(doxorubicin or 14 hydroxydaunomycin or 14 hydroxydaunorubicin or ad mycin or adriablastin or adriacin or adriamicin or adriamycin or	60 414

	adriblastin or adrim or adrubicin or amminac or caelix or caelyx or caelyx?doxil or carcinocin or dexorubicin or dox sl or doxil or doxolem or doxor lyo or doxorubin or evacet or farmiblastina or fi 106 or fi106 or ifadox or lipodox or myocet or nsc 123127 or nsc123127 or rastocin or resmycin or rp 25253 or rp25253 or rubex or rubidox or sarcodoxome or tlc d 99).mp.	
14	(paclitaxel or "abi 007" or abi007 or abraxane or anzatax or asotax or biotax or bms 181339 or bms181339 or bristaxol or britaxol or coroxane or formoxol or genexol or hunxol or ifaxol or intaxel or medixel or mitotax or nsc 125973 or nsc125973 or onxol or pacitaxel or pacxel or padexol or parexel or paxceed or paxene or paxus or praxel or taxocris or taxol or taxus or taycovit or yewtaxan or docetaxel or daxotel or dexotel or docefrez or lit 976 or lit976 or nsc 628503 or nsc628503 or oncodocel or rp 56976 or rp56976 or taxoter or texot or ixabepilone or azaepothilone B or bms 247550 or bms 247550-1 or bms 247550 1 or bms247550 or bms247550?1 or bms247550 1 or ixempra or nsc 710428 or nsc710428 or abraxane or "abi 007" or abi007 or abraxane or anzatax or asotax or biotax or bms 181339 or bms181339 or bristaxol or britaxol or coroxane or formoxol or genexol or hunxol or ifaxol or intaxel or medixel or mitotax or nsc 125973 or nsc125973 or onxol or pacitaxel or pacxel or padexol or parexel or paxceed or paxene or paxus or praxel or taxocris or taxol or taxus or taycovit or yewtaxan or tamoxifen or kessar or nsc 180973 or tamoplac or tamoxasta or toremifene or estrimex or fareston or fc 1157 a or fc 1157a or fc1157a).mp.	65 485
15	(fulvestrant or faslodex or ici 182 780 or ici 182, 780 or ici 182780 or ici182780 or zd 182780 or zd 9238 or zd182780 or zd9238 or zm 182780 or zm182780 or anastrazole or arimidex or ici d1033 or icid1033 or trozolet or zd 1033 or zd1033 or goserelin or buserelin carbazamide or ici 118 630 or ici 118630 or ici118630 or prozoladex or zoladex or letrozole or cgs 20267 or cgs20267 or femar or femara or exemestane or aromasin or aromasine or fce 24304 or fce24304 or nikidess or pnu 155971 or pnu155971).mp.	8682
16	(medroxyprogesterone acetate or acetoxymethylprogesterone or amen or aragest or clinofem or clinovir or curretab or cycrin or depo-prodasone or depo-provera or depo-subqprovera or depo prodasone or depo provera or depo subQ provera or depoclinovir or depomedroxyprogesterone or depoprodasone or depopromone or depoprovera or estrofarlutal or	7493

	farkital or farlutal or gestapolar or gestapuran or gestapuron or hysron h or lutopolar or lutoral farmit or manodepa or medioxyprogesterone acetate or medroxy progesterone acetate or medroxyprogesteronacetate or medroxyprogesterone 17-acetate or medroxyprogesterone 17 acetate or meges or megestron or meprate or methylacetoxyprogesterone or methylpregnone or mpa gyn 5 or nsc 26 386 or nsc 26386 or nsc26386 or oragest or perkitex or perlutex or prodafem or prodasone or progen or progevera or prothyra or provera or ralovera or repromap or veramix or veraplex).mp.	
17	(megestrol or megestrole or navelbine or navelbin or vinbine or vinelbine or navelbine or vinorelbine).mp.	5710
18	(leuprorelin or a 43818 or a43818 or abbott 43818 or carcinil or depo lupron or eligard or enanton or ginecrin or leuplin or leuprogel or leuprolid or leupron or lorelin depot or lucrin or lupride or luprolex or lupron or procren depot or procrin or prostap or reliser or tap 144 or tap144 or tapros or trenantone or viadur or taxane derivatives or taxan* or trastuzumab emtansine or tdm1 or tdm 1 or tdm?1 or t dm 1 or trastuzumab emtansine).mp.	8001
19	(antineoplastic agent or antineoplastic or antitumor or anticancer or tumor inhibitor or ((biologic* or hormon* or endocrine or target*) and (therap* or treatment)) or (angiogenesis inhibitor or neovascularization inhibitor or monoclonal antibody or her2 inhibitor or her 2 inhibitor or her-2 inhibitor or angiogen inhibitor or her2 antibody or her 2 antibody or her-2 antibody or ErbB2 inhibitor or ErbB2 inhibitor or ErbB2 inhibitor or angiogen inhibitor or ErbB2 antibody or ErbB2 antibody or ErbB2 antibody or Erb-B2 inhibitor or Erb-B2 inhibitor or Erb-B2 inhibitor or angiogen inhibitor or Erb-B2 antibody or Erb-B2 antibody or Erb-B2 antibody)).mp.	1 329 918
20	(androgen or anti estrogen or anti oestrogen or hormonal therapy or endocrine therapy or endocrine treatment or endocrinotherapy or hormone therapy or hormone treatment or molecularly targeted therapy or molecular target therapy or molecular targeted therapy or targeted cancer therapy or targeted molecular therapy or targeted therapy or systemic therapy or systemic treatment or biological therapy or biologic therapies or biologic therapy or biological therapies or biotherapies or organotherapy or tissue therapy or anti cancer drug or anti neoplastic agent or antineoplastic or anticancer agent or anticancer drug or	581 347

	anticancerogen or anticarcinogen or antitumor or cancer inhibitor or carcinostatic drug or target therapy or aromatase inhibitor or aromatase inhibitors).mp.	
21	or/6-20	1 531 111
22	4 and 5 and 21	3527
23	limit 22 to “ed=20121001-20160630"	1440

3. Cochrane (searched 30 June 2016)

#	Search terms	Results
1	exp Breast Neoplasms/	8825
2	exp Breast/	636
3	Breast.mp.	28 103
4	1 or 2 or 3	28 114
5	exp Neoplasm Metastasis/	3828
6	(advanced or metastat* or refract* or recurren* or salva* or (late adj stage) or resistan* or stage III or (stage and III*) or stage IV or stage 3 or stage 4 or stage IIIC or stage IIIB or unresect*).mp.	123 908
7	exp Neoplasm Recurrence, Local/	3427
8	5 or 6 or 7	124 919
9	exp Receptor, Epidermal Growth Factor/	394
10	exp Receptor, erbB-2/	502
11	(HER2 or HER-2 or HER 2 or HER positive or HER-positive or HER-overexpressing or HER overexpressing or ErbB2 or Erb-B2 or Human epidermal growth factor receptor 2 or cerbB 2 or Her2neu).mp.	2001
12	9 or 10 or 11	2394
13	(Second-line or Second line or Secondary or Progress* or Relapse* or Recurren* or Fail* or Resist* or Pretreated or pre treated or pre-treated or Refract* or Salvage or Prior or Previous*).mp.	278 783
14	exp Antineoplastic Protocols/	11 453
15	exp Drug Therapy/	121 074
16	((Biologic* or chemo* or systemic or target*) adj2 (therap* or treat*)).mp.	19 477

17	(antineoplastic agent or antineoplastic or antitumor or anticancer or tumor inhibitor or anti tumor or anti tumour or antitumour).mp.	24 546
18	(angiogenesis inhibitor or neovascularization inhibitor or monoclonal antibody).mp.	3214
19	(her2 inhibitor or her 2 inhibitor or angiogen inhibitor or her2 antibody or her 2 antibody or ErbB2 inhibitor or ErbB2 antibody).mp.	21
20	exp Anthracyclines/	4134
21	Anthracycline.mp.	1383
22	exp Cyclophosphamide/	3977
23	exp Methotrexate/	2919
24	exp Fluorouracil/	4069
25	exp Cisplatin/	3395
26	exp Carboplatin/	1062
27	exp Platinum/	111
28	exp Epirubicin/	812
29	exp Doxorubicin/	3477
30	exp Paclitaxel/	1572
31	exp Tamoxifen/	1499
32	exp Toremifene/	68
33	exp Goserelin/	382
34	exp Medroxyprogesterone Acetate/	835
35	exp Megestrol/	240
36	exp Leuprolide/	489
37	(gemcitabine or gemcite or gemzar or ly 188011 or ly188011).mp.	2369
38	(sunitinib or pha 2909040ad or pha2909040ad or "su 010398" or "su 011248" or su 10398 or su 11248 or su010398 or su011248 or su10398 or su11248 or sutent).mp.	428
39	(bevacizumab or avastin or nsc 704865 or nsc704865).mp.	2088
40	((trastuzumab or herceptin or aromatase inhibitor or aomat*) adj2 inhibit*).mp.	766

41	(lapatinib or gw 2016 or gw 572016 or gw 572016f or gw2016 or gw572016 or gw572016f or tykerb or tyverb).mp.	362
42	(pertuzumab or 2C4 or monoclonal antibody 2C4 or omnitarg or r 1273 or r1273 or rhumab 2C4).mp.	101
43	(neratinib or hki 272 or hki272 or way 177820 or way177820).mp.	15
44	(ertumaxomab or rexomun).mp.	0
45	(dasatinib or bms 354825 or bms354825 or sprycel).mp.	178
46	(cyclophosphamide or alkyroxan or b 518 or b518 or carloxan or ciclofosfamida or ciclolen or cicloxal or clafen or cyclo-cell or cycloblastin or cyclofos amide or cyclofosfamid or cyclophar or cyclophosphamid or cyclophosphan or cyclostin or cycloxan or cyphos or cytophosphan or cytoxan or endocyclo phosphate or endoxan or endoxon-asta or enduxan or genoxal or ledoxan or ledoxina or mitoxan or neosan or neosar or noristan or nsc 26271 or nsc 2671 or procytox or procytoxide or semdoxan or sendoxan or syklofosfamid).mp.	8375
47	(methotrexate or methopterine or abitrexate or amethopterin or ametopterine or antifolan or biotrexate or canceren or cl 14377 or cl14377 or emtexate or emthexat or emtrexate or enthexate or farmitrexat or farmitrexate or farmotrex or folex or ifamet or lantarel or ledertrexate or maxtrex or metex or methoblastin or methohexate or methotrate or methotrexat or methotrexato or methoxtrexate or methrotrexate or methylaminopterin or methylaminopterine or meticil or metothrexate or metotrexat or metotrexate or metotrexin or metrex or mexate or mexate-aq or mpi 5004 or mpi5004 or MTX or neotrexate or novatrex or nsc 740 or nsc740 or reumatrex or rheumatrex or rheumatrex dose pack or texate or texorate or trexall or xaken or zexate or MTX).mp.	7072
48	(fluorouracil or 5 fluoruracil or 5 fu or accusite or actino-hermal or adrucil or carac or effluderm or efudex or efudix or efurix or f6627 or fivoflu or fluoro uracil or fluoroblastin or fluoroplex or fluorouracil 5 or fluoruracil or fluouracil or fluoxan or fluracedyl or fluracil or fluracilium or fluril or fluro uracil or fluroblastin or ifacil or nsc 18913 or nsc 19893 or nsc18913 or nsc19893 or oncofu or ro 2-9757 or ro 2 9757 or ro2-9757 or ro2 9757 or uflahex or utoral or verrumal).mp.	8336
49	(cisplatin or abiplatin or biocisplatinum or biocysplatinum or blastolem or briplatin or platinum or cddp ti or cis-platinum or cis ddp or cis	10 081

	diamine dichloroplatinum or cis diaminechloroplatinum or cis platinous diamino dichloride or cis platinum or cytoplatin or cytosplat or docistin or elvecis or kemoplat or lederplatin or mpi 5010 or mpi5010 or neoplatin or niyaplat or nk 801 or noveldexis or nsc 119875 or platamine or platamine rtu or platiblastin or platidiam or platimine or platinex or platinil or platinol or platinol-aq or platinol aq or platinoxan or platiran or platistil or platistin or platosin or randa or romcis or sicatem or "spi 077" or tecnoplatin).mp.	
50	(carboplatin or blastocarb or boplatex or carboplat or carbosin or carbotec or carplan or CBDCA or erbakar or ercar or ifacap or jm-8 or jm 8 or kemocarb or nsc 241240 or oncocarbin or paraplatin or paraplatin or paraplatine).mp.	3294
51	Platinum.mp.	2515
52	(oxaliplatin or crisapla or dacotin or dacplat or eloxatin or eloxatine or heloxatin or oplat or oxalip or oxaltic or transplastin or xaliplat).mp.	1555
53	(capecitabine or apecitab or ro 09-1978 or "ro 09 1978" or "ro 091978" or ro09-1978 or ro09 1978 or ro091978 or xeloda).mp.	1517
54	(vinorelbine or navelbin or vinbine or vinelbine or navelbine or vinorelbine).mp.	1072
55	(epirubicin or epiadriamycin or epidoxorubicin or binarin or ellence or epi-cell or epiadriamycin or epidoxo or epidx or epifil or epilem or farmorrubicina rtu or farmorubicin or imi 28 or nsc 256942 or pharmorubicin or pidorubicin).mp.	2269
56	(doxorubicin or 14 hydroxydaunomycin or 14 hydroxydaunorubicin or ad mycin or adriablastin or adriacin or adriamicin or adriamycin or adriblastin or adrim or adrubicin or amminac or caelix or caelyx or doxil or carcinocin or dexorubicin or dox sl or doxil or doxolem or doxor lyo or doxorubin or evacet or farmiblastina or fi 106 or fi106 or ifadox or lipodox or myocet or nsc 123127 or nsc123127 or rastocin or resmycin or rp 25253 or rp25253 or rubex or rubidox or sarcodoxome or tlc d 99).mp.	6172
57	(paclitaxel or "abi 007" or abi007 or abraxane or anzatax or asotax or biotax or bms 181339 or bms181339 or bristaxol or britaxol or coroxane or formoxol or genexol or hunxol or ifaxol or intaxel or medixel or mitotax or nsc 125973 or nsc125973 or onxol or pacitaxel or pacxel or	4699

	padexol or parexel or paxceed or paxene or paxus or praxel or taxocris or taxol or taxus or taycovit or yewtaxan).mp.	
58	(docetaxel or daxotel or dexotel or docefrez or lit 976 or lit976 or nsc 628503 or nsc628503 or oncodocel or rp 56976 or rp56976 or taxoter or texot).mp.	3123
59	(ixabepilone or azaepothilone B or bms 247550 or bms 247550-1 or bms 247550 1 or bms247550 or bms247550-1 or bms247550 1 or ixempra or nsc 710428 or nsc710428).mp.	88
60	(abraxane or "abi 007" or abi007 or abraxane or anzatax or asotax or biotax or bms 181339 or bms181339 or bristaxol or britaxol or coroxane or formoxol or genexol or hunxol or ifaxol or intaxel or medixel or mitotax or nsc 125973 or nsc125973 or onxol or pacitaxel or pacxel or padexol or parexel or paxceed or paxene or paxus or praxel or taxocris or taxol or taxus or taycovit or yewtaxan).mp.	660
61	(tamoxifen or kessar or nsc 180973 or tamoplac or tamoxasta).mp.	3947
62	(toremifene or estrimex or fareston or fc 1157 a or fc 1157a or fc1157a).mp.	147
63	(fulvestrant or faslodex or ici 182 780 or ici 182, 780 or ici 182780 or ici182780 or zd 182780 or zd 9238 or zd182780 or zd9238 or zm 182780 or zm182780).mp.	215
64	(anastrazole or arimidex or ici d1033 or icid1033 or trozolet or zd 1033 or zd1033).mp.	218
65	(goserelin or buserelin carbazamide or ici 118 630 or ici 118630 or ici118630 or prozoladex or zoladex).mp.	809
66	(letrozole or cgs 20267 or cgs20267 or femar or femara).mp.	887
67	(exemestane or aromasin or aromasine or fce 24304 or fce24304 or nikidess or pnu 155971 or pnu155971).mp.	485
68	(medroxyprogesterone acetate or acetoxymethylprogesterone or amen or aragest or clinofem or clinovir or curretab or cycrin or depo-prodasone or depo-provera or depo-subqprovera or depo prodasone or depo provera or depo subQ provera or depoclinovir or depomedroxyprogesterone or depoprodasone or depopromone or depoprovera or estrofarlutal or farkital or farlutal or gestapolar or gestapuran or gestapuron or hysron h or lutopolar or lutoral farmit or manodepa or medioxyprogesterone acetate or medroxy progesterone acetate or medroxyprogesteronacetate	1737

	or medroxyprogesterone 17-acetate or medroxyprogesterone 17 acetate or meges or megestron or meprate or methylacetoxyprogesterone or methylpregnone or mpa gyn 5 or nsc 26 386 or nsc 26386 or nsc26386 or oragest or perkitex or perlutex or prodafem or prodasone or progen or progevera or prothyra or provera or ralovera or repromap or veramix or veraplex).mp.	
69	(megestrol or megestrole).mp.	502
70	(navelbine or navelbin or vinbine or vinelbine or navelbine or vinorelbine).mp.	1072
71	(leuprorelin or a 43818 or a43818 or abbott 43818 or carcinil or depo lupron or eligard or enanton or ginecrin or leuplin or leuprogel or leuprolid or leupron or lorelin depot or lucrin or lupride or luprolex or lupron or procren depot or procrin or prostap or reliser or tap 144 or tap144 or tapros or trenantone or viadur).mp.	417
72	(taxane derivatives or taxan*).mp.	1160
73	(trastuzumab emtansine or tdm1 or tdm 1 or tdm?1 or t dm 1 or trastuzumab emtansine).mp.	44
74	exp Aromatase Inhibitors/	530
75	or/14-74	165 479
76	4 and 8 and 12 and 13 and 75	968
77	limit 76 to “ed=20121001-20160630" [Limit not valid in DARE; records were retained]	452

C. Second systematic review update (1 January 2016 to 3 January 2018)

1. Embase (searched 3 January 2018)

#	Search terms	Results
1	exp clinical trial/	1 297 868
2	randomization/	76 800
3	controlled study/	5 914 443
4	'comparative study'/	779 814
5	single blind procedure/	30 759
6	'double blind procedure'/	147 410
7	'crossover procedure'/	54 790
8	'placebo'/	320 741
9	(clinical trial' or 'clinical trials').mp.	1 544 074
10	(controlled clinical trial or controlled clinical trials).mp.	483 094
11	('randomised controlled trial' or 'randomized controlled trial' or 'randomised controlled trials' or 'randomized controlled trials').mp.	678 340
12	('randomisation' or 'randomization' or random*).mp.	1 482 743
13	RCT.mp.	28 512
14	'random allocation'.mp.	1911
15	'randomly allocated'.mp.	29 494
16	'allocated randomly'.mp.	2327
17	((allocated adj2 random) or (assign* adj2 random*)).mp.	122 075
18	((single or double or triple or treble) adj (blind* or mask*)).mp.	270 030
19	placebo*.mp.	413 944
20	'prospective study'/	429 686
21	or/1-20	8 249 162
22	'case study'/	52 224
23	'case report'.mp.	2 301 053
24	'abstract report'/	89 737
25	'letter'/	958 183
26	or/22-25	3 199 382
27	21 not 26	8 057 012
28	(nrct or n rct or n?rct).mp.	144
29	exp 'controlled clinical trial'/	666 832
30	exp Prospective study/	429 686

31	exp Major clinical study/	3 043 922
32	exp Intervention study/	35 708
33	(clinical adj trial*).ti,ab.	423 565
34	or/27-33	9 585 955
35	exp 'chemotherapy'/ or chemotherap*.mp. or ((chemo* or biologic* or hormon* or endocrin* or target*) adj2 (therap* or treat*)).mp.	1 149 995
36	antineoplastic agent/ or (antineoplastic drug or anticancer drug or tumor inhibitor or anticarcinogenic agents or anticarcinogenic agents).mp.	275 522
37	'biological therapy'/ or (biologic therapy or tissue therapy).mp.	13 912
38	'systemic therapy'/	23 686
39	'molecularly targeted therapy'/	22 750
40	'hormonal therapy'/ or (endocrine therapy or endocrine treatment).mp.	42 960
41	'angiogenesis inhibitor'/	16 260
42	aromatase inhibitor/	12 839
43	(((her2 or 'her 2' or 'her 2' or angiogen*) adj4 inhibit*) or ((antiangiogen* or anti angiogen or her 2 or her2 or erbb2 or erbb 2) adj2 antibody)).mp.	36 786
44	(androgen* or anti estrogen or anti oestrogen).mp.	122 104
45	anthracycline/ or anthracycline*.mp.	34 095
46	gemcitabine'/ or (gemcite or gemzar or ly 188011 or ly188011).mp.	48 067
47	'sunitinib'/ or (pha 2909040ad or pha2909040ad or "su 010398" or "su 011248" or su 10398 or su 11248 or su010398 or su011248 or su10398 or su11248 or sutent).mp.	19 570
48	bevacizumab/ or (avastin or nsc 704865 or nsc704865).mp.	48 762
49	trastuzumab/ or herceptin.mp.	32 942
50	'lapatinib'/ or (gw 2016 or gw 572016 or gw 572016f or gw2016 or gw572016 or gw572016f or tykerb or tyverb).mp.	10 555
51	'pertuzumab'/ or (2C4 or monoclonal antibody 2C4 or omnitarg or r 1273 or r1273 or rhumab 2C4).mp.	3574
52	'neratinib'/ or (hki 272 or hki272 or way 177820 or way177820).mp.	1286
53	'ertumaxomab'/ or rexomun.mp.	122
54	'dasatinib'/ or (bms 354825 or bms354825 or sprycel).mp.	11 230
55	'cyclophosphamide'/ or (alkyroxan or b 518 or b518 or carloxan or ciclofosfamida or ciclolen or cicloxal or clafen or cyclo-cell or cycloblastin or cyclofos amide or cyclofosfamid or cyclophar or cyclophosphamid or cyclophosphan or cyclostin or cycloxan or cyphos or cytophosphan or cytoxan or endocyclo phosphate or endoxan or endoxon-asta or enduxan or genoxal or ledoxan or ledoxina or mitoxan or neosan or neosar or noristan or nsc 26271 or nsc 2671 or procytox or procytoxide or semdoxan or sendoxan or syklofosfamid).mp.	200 608
56	'methotrexate'/ or (methopterine or abitrexate or amethopterin or ametopterine or antifolan or biotrexate or canceren or cl 14377 or cl14377 or emtexate or emthexat or emtrexate or enthexate or farmitrexat or farmitrexate or farmotrex or folex or ifamet or lantarel or ledertrexate or maxtrex or metex or	164 309

	methoblastin or methohexate or methotrate or methotrexat or methotrexato or methoxtrexate or methrotrexate or methylaminopterin or methylaminopterine or meticil or metothrexate or metotrexat or metotrexate or metotrexin or metrex or mexate or mexate-aq or mpi 5004 or mpi5004 or MTX or neotrexate or novatrex or nsc 740 or nsc740 or reumatrex or rheumatrex or rheumatrex dose pack or texate or texorate or trexall or xaken or zexate or MTX).mp.	
57	fluorouracil/ or (5 fluoruracil or 5 fu or accusite or actino-hermal or adrucil or carac or effluderm or efudex or efudix or efurix or f6627 or fivoflu or fluoro uracil or fluoroblastin or fluoroplex or fluorouracil 5 or fluoruracil or fluouracil or fluoxan or furacedyl or fluracil or fluracilium or fluril or fluro uracil or fluroblastin or ifacil or nsc 18913 or nsc 19893 or nsc18913 or nsc19893 or oncofu or ro 2-9757 or ro 2 9757 or ro2-9757 or ro2 9757 or uflahex or utoral or verrumal).mp.	130 437
58	cisplatin/ or (abiplatin or biocisplatinum or biocysplatinum or blastolem or briplatin or platinum or cddp ti or cis-platinum or cis ddp or cis diamine dichloroplatinum or cis diaminechloroplatinum or cis platinous diamino dichloride or cis platinum or cytoplatin or cytosplat or docistin or elvecis or kemoplat or lederplatin or mpi 5010 or mpi5010 or neoplatin or niyaplat or nk 801 or noveldexis or nsc 119875 or platamine or platamine rtu or platiblastin or platidiam or platimine or platinex or platinil or platinol or platinol-aq or platinol aq or platinoxan or platiran or platistil or platistin or platosin or randa or romcis or sicatem or "spi 077" or tecnoplatin).mp.	204 526
59	carboplatin/ or (blastocarb or boplatex or carboplat or carbosin or carbotec or carplan or CBDCA or erbakar or ercar or ifacap or jm-8 or jm 8 or kemocarb or nsc 241240 or oncocarbin or paraplatin or paraplatin or paraplatine).mp.	59 642
60	platinum/	26 954
61	oxaliplatin/ or (crisapla or dacotin or dacplat or eloxatin or eloxatine or heloxatin or oplat or oxalip or oxaltic or transplastin or xaliplat).mp.	32 243
62	capecitabine/ or (apecitab or ro 09-1978 or "ro 09 1978" or "ro 091978" or ro09-1978 or ro09 1978 or ro091978 or xeloda).mp.	24 596
63	vinorelbine/ or (navelbin or vinbine or vinelbine or navelbine or vinorelbine).mp.	16 825
64	epirubicin/ or (epiadriamycin or epidoxorubicin or binarin or ellence or epi-cell or epiadriamycin or epidoxo or epidx or epifil or epilem or farmorrubicina rtu or farmorubicin or imi 28 or nsc 256942 or pharmorubicin or pidorubicin).mp.	26 433
65	doxorubicin/ or (14 hydroxydaunomycin or 14 hydroxydaunorubicin or ad mycin or adriablastin or adriacin or adriamicin or adriamycin or adriblastin or adrim or adrubicin or amminac or caelix or caelyx or caelyx/doxil or carcinocin or dexorubicin or dox sl or doxil or doxolem or doxor lyo or doxorubin or evacet or farmiblastina or fi 106 or fi106 or ifadox or lipodox or myocet or nsc 123127 or nsc123127 or rastocin or resmycin or rp 25253 or rp25253 or rubex or rubidox or sarcodoxome or tlc d 99).mp.	172 041
66	paclitaxel/ or ("abi 007" or abi007 or abraxane or anzatax or asotax or biotax or bms 181339 or bms181339 or bristaxol or britaxol or coroxane or formoxol or genexol or hunxol or ifaxol or intaxel or medixel or mitotax or nsc 125973 or nsc125973 or onxol or pacitaxel or pacxel or padexol or parexel or paxceed	95 553

	or paxene or paxus or praxel or taxocris or taxol or taxus or taycovit or yewtaxan).mp.	
67	docetaxel/ or (daxotel or dexotel or docefrez or lit 976 or lit976 or nsc 628503 or nsc628503 or oncodocel or rp 56976 or rp56976 or taxoter or texot).mp.	50 136
68	ixabepilone/ or (azaepothilone B or bms 247550 or bms 247550-1 or bms 247550 1 or bms247550 or bms247550-1 or bms247550 1 or ixempra or nsc 710428 or nsc710428).mp.	1670
69	abraxane/ or ("abi 007" or abi007 or abraxane or anzatax or asotax or biotax or bms 181339 or bms181339 or bristaxol or britaxol or coroxane or formoxol or genexol or hunxol or ifaxol or intaxel or medixel or mitotax or nsc 125973 or nsc125973 or onxol or pacitaxel or pacxel or padexol or parexel or paxceed or paxene or paxus or praxel or taxocris or taxol or taxus or taycovit or yewtaxan).mp.	95 553
70	tamoxifen/ or (kessar or nsc 180973 or tamoplac or tamoxasta).mp.	57 029
71	toremifene/ or (estrimex or fareston or fc 1157 a or fc 1157a or fc1157a).mp.	2030
72	fulvestrant/ or (faslodex or ici 182 780 or ici 182, 780 or ici 182780 or ici182780 or zd 182780 or zd 9238 or zd182780 or zd9238 or zm 182780 or zm182780).mp.	8138
73	anastrazole/ or (arimidex or ici d1033 or icid1033 or trozolet or zd 1033 or zd1033).mp.	8683
74	goserelin/ or (buserelin carbazamide or ici 118 630 or ici 118630 or ici118630 or prozoladex or zoladex).mp.	6661
75	letrozole/ or (cgs 20267 or cgs20267 or femar or femara).mp.	10 203
76	exemestane/ or (aromasin or aromasine or fce 24304 or fce24304 or nikidess or pnu 155971 or pnu155971).mp.	5447
77	medroxyprogesterone acetate/ or (acetoxymethylprogesterone or amen or aragest or clinofem or clinovir or curretab or cycrin or depo-prodasone or depo-provera or depo-subqprovera or depo prodasone or depo provera or depo subQ provera or depoclinovir or depomedroxyprogesterone or depoprodasone or depopromone or depoprovera or estrofarlutal or farkital or farlutal or gestapolar or gestapuran or gestapuron or hysron h or lutopolar or lutoral farmit or manodepa or medioxyprogesterone acetate or medroxy progesterone acetate or medroxyprogesteronacetate or medroxyprogesterone 17-acetate or medroxyprogesterone 17 acetate or meges or megestron or meprate or methylacetoxyprogesterone or methylpregnone or mpa gyn 5 or nsc 26 386 or nsc 26386 or nsc26386 or oragest or perkitex or perlutex or prodafem or prodasone or progen or progevera or prothyra or provera or ralovera or repromap or veramix or veraplex).mp.	17 419
78	megestrol/ or megestrole.mp.	1570
79	navelbine/ or (navelbin or vinbine or vinelbine or navelbine or vinorelbine).mp.	16 825
80	leuprorelin/ or (a 43818 or a43818 or abbott 43818 or carcinil or depo lupron or eligard or enanton or ginecrin or leuplin or leuprogel or leuprolid or leupron or lorelin depot or lucrin or lupride or luprolex or lupron or procren depot or procrin or prostap or reliser or tap 144 or tap144 or tapros or trenantone or viadur).mp.	10 401
81	(taxane derivatives or taxan*).mp.	22 738

82	trastuzumab emtansine/ or (tdm1 or tdm 1 or tdm?1 or t dm 1 or trastuzumab emtansine).mp.	1956
83	or/35-82	1 822 056
84	exp breast tumor/ or (breast tumour or breast tumor).mp.	468 498
85	exp breast/ or breast.mp.	684 100
86	exp breast neoplasms/ or ((breast neoplasm or breast) adj5 carcinoma).mp. or (breast adj5 cancer).mp. or (breast adj5 malignan*).mp.	529 423
87	or/84-86	685 980
88	((advanced or metastat* or refract* or recurren* or salva* or late) adj2 (stage or resistan* or stage iii or (stage and iii*) or stage iv or stage 3 or stage 4)).mp. or exp breast metastasis/ or exp metastasis/ or exp recurrent disease/	764 233
89	87 and 88	105 971
90	epidermal growth factor receptor 2/ or (her2 or her 2 or her-2).mp.	67 755
91	(second* or progress* or relapse* or recurren* or fail* or resistance or pretreated or pre treated or pre-treated or refract* or previous* or salvage or prior or second-line or second line).mp.	8 179 323
92	34 and 83 and 89 and 90 and 91	4946
93	limit 92 to yr="2016 -Current"	1526

2. Medline (searched 3 January 2018)

#	Searches	Results
1	(Breast Neoplasms or Breast cancer or Breast cancers or Breast neoplasm or Breast neoplasms or Breast tumour or Breast tumor or Breast tumors or Mammary carcinoma or Mammary carcinomas or Mammary neoplasm or Mammary neoplasms or Breast tumours).ti,ab.	270 350
2	(HER2 or HER-2 or HER-positive or HER -overexpressing or ErbB2 or Erb-B2 or Human epidermal growth factor receptor 2 or cerbB 2 or Her2neu).mp. or Her2/	37 262
3	(Advanced or Metastatic or Stage 3 or Stage 4 or Stage III or Stage IIIB or Stage IIIC or Stage IV or Metastasis or Metastases or Unresectable or Inoperable).mp.	909 423
4	1 and 2 and 3	10 481
5	Second-line.ti,ab. or Second line,ti.ab. or Secondary.ti,ab. or Progression.ti,ab. or Progressed.ti,ab. or Progressive.ti,ab. or Relapse*.ti,ab. or Recurren*.ti,ab. or Failed.ti,ab. or Failure.ti,ab. or Resistant.ti,ab. or Resistance.ti,ab. or Pretreated.ti,ab. or Refractory.ti,ab. or Previously.ti,ab. or Salvage.ti,ab. or Prior.ti,ab. or Previous*.ti,ab. or Previous.ti,ab.	5 357 112
6	(gemcitabine or gemcite or gemzar or ly 188011 or ly188011 or sunitinib or pha 2909040ad or pha2909040ad or "su 010398" or "su 011248" or su 10398 or su 11248 or su010398 or su011248 or su10398 or su11248 or sutent or bevacizumab or avastin or trastuzumab or herceptin or lapatinib or gw 2016 or gw 572016 or gw 572016f or gw2016 or gw572016 or gw572016f or tykerb or tyverb or pertuzumab or 2C4 or monoclonal antibody 2C4 or omnitarg or r 1273 or r1273 or rhumab 2C4 or neratinib or hki 272 or hki272 or way 177820	

	or way177820 or ertumaxomab or rexomun or dasatinib or bms 354825 or bms354825 or sprycel).mp.	
7	(cyclophosphamide or alkyroxan or b 518 or b518 or carloxan or ciclofosfamida or ciclolen or cicloxal or clafen or cyclo-cell or cycloblastin or cyclofos amide or cyclofosfamid or cyclophar or cyclophosphamid or cyclophosphan or cyclostin or cycloxan or cyphos or cytophosphan or cytoxan or endocyclo phosphate or endoxan or endoxon-asta or enduxan or genoxal or ledoxan or ledoxina or mitoxan or neosan or neosar or noristan or nsc 26271 or nsc 2671 or procytox or procytoxide or semdoxan or sendoxan or syklofosfamid or methotrexate or methopterine or abitrexate or amethopterin or ametopterine or antifolan or biotrexate or canceren or cl 14377 or cl14377 or emtexate or emthexat or emtrexate or enthexate or farmitrexat or farmitrexate or farmotrex or folex or ifamet or lantarel or ledertrexate or maxtrex or metex or methoblastin or methohexate or methotrate or methotrexat or methotrexato or methoxtrexate or methrotrexate or methylaminopterin or methylaminopterine or meticil or metothrexate or metotrexat or metotrexate or metotrexin or metrex or mexate or mexate-aq or mpi 5004 or mpi5004 or MTX or neotrexate or novatrex or nsc 740 or nsc740 or reumatrex or rheumatrex or rheumatrex dose pack or texate or texorate or trexall or xaken or zexate or MTX).mp.	119 560
8	(fluorouracil or 5 fluoruracil or 5 fu or accusite or actino-hermal or adrucil or carac or effluderm or efudex or efudix or efurix or f6627 or fivoflu or fluoro uracil or fluoroblastin or fluoroplex or fluorouracil 5 or fluoruracil or fluouracil or fluoxan or fluracedyl or fluracil or fluracilium or fluril or fluro uracil or fluroblastin or ifacil or nsc 18913 or nsc 19893 or nsc18913 or nsc19893 or oncofu or ro 2-9757 or ro 2 9757 or ro2-9757 or ro2 9757 or uflahex or utoral or verrumal or cisplatin or abiplatin or biocisplatinum or biocysplatinum or blastolem or briplatin or platinum or cddp ti or cis-platinum or cis ddp or cis diamine dichloroplatinum or cis diaminechloroplatinum or cis platinous diamino dichloride or cis platinum or cytoplatin or cytosplat or docistin or elvecis or kemoplat or lederplatin or mpi 5010 or mpi5010 or neoplatin or niyaplat or nk 801 or noveldexis or nsc 119875 or platamine or platamine rtu or platiblastin or platidiam or platimine or platinex or platinil or platinol or platinol-aq or platinol aq or platinoxan or platiran or platistil or platistin or platosin or randa or romcis or sicatem or "spi 077" or tecnoplatin or carboplatin or blastocarb or boplatex or carboplat or carbosin or carbotec or carplan or CBDCA or erbakar or ercar or ifacap or jm-8 or jm 8 or kemocarb or nsc 241240 or oncocarbin or paraplatin or paraplatin or paraplatine or platinum).mp.	161 196
9	(oxaliplatin or crisapla or dacotin or dacplat or eloxatin or eloxatine or heloxatin or oplat or oxalip or oxaltic or transplastin or xaliplat).mp.	10 833
10	(capecitabine or apecitab or ro 09-1978 or "ro 09 1978" or "ro 091978" or ro09-1978 or ro09 1978 or ro091978 or xeloda).mp.	6857
11	(vinorelbine or navelbin or vinbine or vinelbine or navelbine or vinorelbine).mp.	4387
12	(epirubicin or epiadriamycin or epidoxorubicin or binarin or ellence or epi-cell or epiadriamycin or epidoxo or epidx or epifil or epilem or farmorrubicina rtu or farmorubicin or imi 28 or nsc 256942 or pharmorubicin or pidorubicin).mp.	7778

13	(doxorubicin or 14 hydroxydaunomycin or 14 hydroxydaunorubicin or ad mycin or adriablastin or adriacin or adriamicin or adriamycin or adriblastin or adrim or adrubicin or amminac or caelix or caelyx or caelyx?doxil or carcinocin or dexorubicin or dox sl or doxil or doxolem or doxor lyo or doxorubin or evacet or farmiblastina or fi 106 or fi106 or ifadox or lipodox or myocet or nsc 123127 or nsc123127 or rastocin or resmycin or rp 25253 or rp25253 or rubex or rubidox or sarcodoxome or tlc d 99).mp.	71 814
14	(paclitaxel or "abi 007" or abi007 or abraxane or anzatax or asotax or biotax or bms 181339 or bms181339 or bristaxol or britaxol or coroxane or formoxol or genexol or hunxol or ifaxol or intaxel or medixel or mitotax or nsc 125973 or nsc125973 or onxol or pacitaxel or pacxel or padexol or parexel or paxceed or paxene or paxus or praxel or taxocris or taxol or taxus or taycovit or yewtaxan or docetaxel or daxotel or dexotel or docefrez or lit 976 or lit976 or nsc 628503 or nsc628503 or oncodocel or rp 56976 or rp56976 or taxoter or texot or ixabepilone or azaepothilone B or bms 247550 or bms 247550-1 or bms 247550 1 or bms247550 or bms247550?1 or bms247550 1 or ixempra or nsc 710428 or nsc710428 or abraxane or "abi 007" or abi007 or abraxane or anzatax or asotax or biotax or bms 181339 or bms181339 or bristaxol or britaxol or coroxane or formoxol or genexol or hunxol or ifaxol or intaxel or medixel or mitotax or nsc 125973 or nsc125973 or onxol or pacitaxel or pacxel or padexol or parexel or paxceed or paxene or paxus or praxel or taxocris or taxol or taxus or taycovit or yewtaxan or tamoxifen or kessar or nsc 180973 or tamoplac or tamoxasta or toremifene or estrimex or fareston or fc 1157 a or fc 1157a or fc1157a).mp.	78 735
15	(fulvestrant or faslodex or ici 182 780 or ici 182, 780 or ici 182780 or ici182780 or zd 182780 or zd 9238 or zd182780 or zd9238 or zm 182780 or zm182780 or anastrazole or arimidex or ici d1033 or icid1033 or trozolet or zd 1033 or zd1033 or goserelin or buserelin carbazamide or ici 118 630 or ici 118630 or ici118630 or prozoladex or zoladex or letrozole or cgs 20267 or cgs20267 or femar or femara or exemestane or aromasin or aromasine or fce 24304 or fce24304 or nikidess or pnu 155971 or pnu155971).mp.	10 201
16	(medroxyprogesterone acetate or acetoxymethylprogesterone or amen or aragest or clinofem or clinovir or curretab or cycrin or depo-prodasone or depo-provera or depo-subqprovera or depo prodasone or depo provera or depo subQ provera or depoclinovir or depomedroxyprogesterone or depoprodasone or depopromone or depoprovera or estrofarlutal or farkital or farlutal or gestapolar or gestapuran or gestapuron or hysron h or lutopolar or lutoral farmit or manodepa or medioxyprogesterone acetate or medroxy progesterone acetate or medroxyprogesteronacetate or medroxyprogesterone 17-acetate or medroxyprogesterone 17 acetate or meges or megestron or meprate or methylacetoxyprogesterone or methylpregnone or mpa gyn 5 or nsc 26 386 or nsc 26386 or nsc26386 or oragest or perkitex or perlutex or prodafem or prodasone or progen or progevera or prothyra or provera or ralovera or repromap or veramix or veraplex).mp.	8327
17	(megestrol or megestrole or navelbine or navelbin or vinbine or vinelbine or navelbine or vinorelbine).mp.	6681
18	(leuprorelin or a 43818 or a43818 or abbott 43818 or carcinil or depo lupron or eligard or enanton or ginecrin or leuplin or leuprogel or leuprolid or leupron or lorelin depot or lucrin or lupride or luprolex or lupron or procren depot or	10 156

	procrin or prostap or reliser or tap 144 or tap144 or tapros or trenantone or viadur or taxane derivatives or taxan* or trastuzumab emtansine or tdm1 or tdm 1 or tdm?1 or t dm 1 or trastuzumab emtansine).mp.	
19	(antineoplastic agent or antineoplastic or antitumor or anticancer or tumor inhibitor or ((biologic* or hormon* or endocrine or target*) and (therap* or treatment)) or (angiogenesis inhibitor or neovascularization inhibitor or monoclonal antibody or her2 inhibitor or her 2 inhibitor or her-2 inhibitor or angiogen inhibitor or her2 antibody or her 2 antibody or her-2 antibody or ErbB2 inhibitor or ErbB2 inhibitor or ErbB2 inhibitor or angiogen inhibitor or ErbB2 antibody or ErbB2 antibody or ErbB2 antibody or Erb-B2 inhibitor or Erb-B2 inhibitor or Erb-B2 inhibitor or angiogen inhibitor or Erb-B2 antibody or Erb-B2 antibody or Erb-B2 antibody)).mp.	1 616 798
20	(androgen or anti estrogen or anti oestrogen or hormonal therapy or endocrine therapy or endocrine treatment or endocrinotherapy or hormone therapy or hormone treatment or molecularly targeted therapy or molecular target therapy or molecular targeted therapy or targeted cancer therapy or targeted molecular therapy or targeted therapy or systemic therapy or systemic treatment or biological therapy or biologic therapies or biologic therapy or biological therapies or biotherapies or organotherapy or tissue therapy or anti cancer drug or anti neoplastic agent or antineoplastic or anticancer agent or anticancer drug or anticancerogen or anticarcinogen or antitumor or cancer inhibitor or carcinostatic drug or target therapy or aromatase inhibitor or aromatase inhibitors).mp.	700 216
21	or/6-20	1 851 653
22	4 and 5 and 21	4957
23	limit 22 to yr="2016 -Current"	1108

3. Cochrane (searched 3 January 2018)

#	Searches	Results
1	exp Breast Neoplasms/	9109
2	exp Breast/	666
3	Breast.mp.	32 818
4	1 or 2 or 3	32 832
5	exp Neoplasm Metastasis/	4008
6	(advanced or metastat* or refract* or recurren* or salva* or (late adj stage) or resistan* or stage III or (stage and III*) or stage IV or stage 3 or stage 4 or stage IIIC or stage IIIB or unresect*).mp.	151 676
7	exp Neoplasm Recurrence, Local/	3722
8	5 or 6 or 7	152 707
9	exp Receptor, Epidermal Growth Factor/	419
10	exp Receptor, erbB-2/	610
11	(HER2 or HER-2 or HER 2 or HER positive or HER-positive or HER-overexpressing or HER overexpressing or ErbB2 or Erb-B2 or Human epidermal growth factor receptor 2 or cerbB 2 or Her2neu).mp.	3224

12	9 or 10 or 11	3649
13	(Second-line or Second line or Secondary or Progress* or Relapse* or Recurren* or Fail* or Resist* or Pretreated or pre treated or pre-treated or Refract* or Salvage or Prior or Previous*).mp.	342 271
14	exp Antineoplastic Protocols/	12 374
15	exp Drug Therapy/	129 040
16	((Biologic* or chemo* or systemic or target*) adj2 (therap* or treat*)).mp.	26 362
17	(antineoplastic agent or antineoplastic or antitumor or anticancer or tumor inhibitor or anti tumor or anti tumour or antitumour).mp.	28 174
18	(angiogenesis inhibitor or neovascularization inhibitor or monoclonal antibody).mp.	4819
19	(her2 inhibitor or her 2 inhibitor or angiogen inhibitor or her2 antibody or her 2 antibody or ErbB2 inhibitor or ErbB2 antibody).mp.	36
20	exp Anthracyclines/	4326
21	Anthracycline.mp.	1866
22	exp Cyclophosphamide/	4145
23	exp Methotrexate/	3085
24	exp Fluorouracil/	4295
25	exp Cisplatin/	3601
26	exp Carboplatin/	1164
27	exp Platinum/	127
28	exp Epirubicin/	868
29	exp Doxorubicin/	3633
30	exp Paclitaxel/	1757
31	exp Tamoxifen/	1549
32	exp Toremifene/	69
33	exp Goserelin/	398
34	exp Medroxyprogesterone Acetate/	859
35	exp Megestrol/	245
36	exp Leuprolide/	509
37	(gemcitabine or gemcite or gemzar or ly 188011 or ly188011).mp.	3197
38	(sunitinib or pha 2909040ad or pha2909040ad or "su 010398" or "su 011248" or su 10398 or su 11248 or su010398 or su011248 or su10398 or su11248 or sutent).mp.	676
39	(bevacizumab or avastin or nsc 704865 or nsc704865).mp.	3213
40	((trastuzumab or herceptin or aromatase inhibitor or aomat*) adj2 inhibit*).mp.	1013
41	(lapatinib or gw 2016 or gw 572016 or gw 572016f or gw2016 or gw572016 or gw572016f or tykerb or tyverb).mp.	473
42	(pertuzumab or 2C4 or monoclonal antibody 2C4 or omnitarg or r 1273 or r1273 or rhumab 2C4).mp.	250
43	(neratinib or hki 272 or hki272 or way 177820 or way177820).mp.	49
44	(ertumaxomab or rexomun).mp.	0
45	(dasatinib or bms 354825 or bms354825 or sprycel).mp.	293

46	(cyclophosphamide or alkyroxan or b 518 or b518 or carloxan or ciclofosfamida or ciclolen or cicloxal or clafen or cyclo-cell or cycloblastin or cyclofos amide or cyclofosfamid or cyclophar or cyclophosphamid or cyclophosphan or cyclostin or cycloxan or cyphos or cytophosphan or cytoxan or endocyclo phosphate or endoxan or endoxon-asta or enduxan or genoxal or ledoxan or ledoxina or mitoxan or neosan or neosar or noristan or nsc 26271 or nsc 2671 or procytox or procytoxide or semdoxan or sendoxan or syklofosfamid).mp.	9671
47	(methotrexate or methopterine or abitrexate or amethopterin or ametopterine or antifolan or biotrexate or canceren or cl 14377 or cl14377 or emtexate or emthexat or emtrexate or enthexate or farmitrexat or farmitrexate or farmotrex or folex or ifamet or lantarel or ledertrexate or maxtrex or metex or methoblastin or methohexate or methotrate or methotrexat or methotrexato or methoxtrexate or methrotrexate or methylaminopterin or methylaminopterine or meticil or metothrexate or metotrexat or metotrexate or metotrexin or metrex or mexate or mexate-aq or mpi 5004 or mpi5004 or MTX or neotrexate or novatrex or nsc 740 or nsc740 or reumatrex or rheumatrex or rheumatrex dose pack or texate or texorate or trexall or xaken or zexate or MTX).mp.	8445
48	(fluorouracil or 5 fluoruracil or 5 fu or accusite or actino-hermal or adrucil or carac or effluderm or efudex or efudix or efurix or f6627 or fivoflu or fluoro uracil or fluoroblastin or fluoroplex or fluorouracil 5 or fluoruracil or fluouracil or fluoxan or fluracedyl or fluracil or fluracilium or fluril or fluro uracil or fluroblastin or ifacil or nsc 18913 or nsc 19893 or nsc18913 or nsc19893 or oncofu or ro 2-9757 or ro 2 9757 or ro2-9757 or ro2 9757 or uflahex or utoral or verrumal).mp.	9351
49	(cisplatin or abiplatin or biocisplatinum or biocysplatinum or blastolem or briplatin or platinum or cddp ti or cis-platinum or cis ddp or cis diamine dichloroplatinum or cis diaminechloroplatinum or cis platinous diamino dichloride or cis platinum or cytoplatin or cytosplat or docistin or elvecis or kemoplat or lederplatin or mpi 5010 or mpi5010 or neoplatin or niyaplat or nk 801 or noveldexis or nsc 119875 or platamine or platamine rtu or platiblastin or platidiam or platimine or platinex or platinil or platinol or platinol-aq or platinol aq or platinoxan or platiran or platistil or platistin or platosin or randa or romcis or sicatem or "spi 077" or tecnoplatin).mp.	12 270
50	(carboplatin or blastocarb or boplatex or carboplat or carbosin or carbotec or carplan or CBDCA or erbakar or ercar or ifacap or jm-8 or jm 8 or kemocarb or nsc 241240 or oncocarbin or paraplatin or paraplatin or paraplatine).mp.	4101
51	Platinum.mp.	3458
52	(oxaliplatin or crisapla or dacotin or dacplat or eloxatin or eloxatine or heloxatin or oplat or oxalip or oxaltic or transplastin or xaliplat).mp.	2234
53	(capecitabine or apecitab or ro 09-1978 or "ro 09 1978" or "ro 091978" or ro09-1978 or ro09 1978 or ro091978 or xeloda).mp.	2091
54	(vinorelbine or navelbin or vinbine or vinelbine or navelbine or vinorelbine).mp.	1219
55	(epirubicin or epiadriamycin or epidoxorubicin or binarin or ellence or epi-cell or epiadriamycin or epidoxo or epidx or epifil or epilem or farmorrubicina rtu or farmorubicin or imi 28 or nsc 256942 or pharmorubicin or pidorubicin).mp.	2561

56	(doxorubicin or 14 hydroxydaunomycin or 14 hydroxydaunorubicin or ad mycin or adriablastin or adriacin or adriamicin or adriamycin or adriblastin or adrim or adrubicin or amminac or caelix or caelyx or doxil or carcinocin or dexorubicin or dox sl or doxil or doxolem or doxor lyo or doxorubin or evacet or farmiblastina or fi 106 or fi106 or ifadox or lipodox or myocet or nsc 123127 or nsc123127 or rastocin or resmycin or rp 25253 or rp25253 or rubex or rubidox or sarcodoxome or tlc d 99).mp.	6992
57	(paclitaxel or "abi 007" or abi007 or abraxane or anzatax or asotax or biotax or bms 181339 or bms181339 or bristaxol or britaxol or coroxane or formoxol or genexol or hunxol or ifaxol or intaxel or medixel or mitotax or nsc 125973 or nsc125973 or onxol or pacitaxel or pacxel or padexol or parexel or paxceed or paxene or paxus or praxel or taxocris or taxol or taxus or taycovit or yewtaxan).mp.	6052
58	(docetaxel or daxotel or dexotel or docefrez or lit 976 or lit976 or nsc 628503 or nsc628503 or oncodocel or rp 56976 or rp56976 or taxoter or texot).mp.	4085
59	(ixabepilone or azaepothilone B or bms 247550 or bms 247550-1 or bms 247550 1 or bms247550 or bms247550-1 or bms247550 1 or ixempra or nsc 710428 or nsc710428).mp.	100
60	(abraxane or "abi 007" or abi007 or abraxane or anzatax or asotax or biotax or bms 181339 or bms181339 or bristaxol or britaxol or coroxane or formoxol or genexol or hunxol or ifaxol or intaxel or medixel or mitotax or nsc 125973 or nsc125973 or onxol or pacitaxel or pacxel or padexol or parexel or paxceed or paxene or paxus or praxel or taxocris or taxol or taxus or taycovit or yewtaxan).mp.	690
61	(tamoxifen or kessar or nsc 180973 or tamoplac or tamoxasta).mp.	4201
62	(toremifene or estrimex or fareston or fc 1157 a or fc 1157a or fc1157a).mp.	150
63	(fulvestrant or faslodex or ici 182 780 or ici 182, 780 or ici 182780 or ici182780 or zd 182780 or zd 9238 or zd182780 or zd9238 or zm 182780 or zm182780).mp.	346
64	(anastrazole or arimidex or ici d1033 or icid1033 or trozolet or zd 1033 or zd1033).mp.	225
65	(goserelin or buserelin carbazamide or ici 118 630 or ici 118630 or ici118630 or prozoladex or zoladex).mp.	869
66	(letrozole or cgs 20267 or cgs20267 or femar or femara).mp.	1164
67	(exemestane or aromasin or aromasine or fce 24304 or fce24304 or nikidess or pnu 155971 or pnu155971).mp.	609
68	(medroxyprogesterone acetate or acetoxymethylprogesterone or amen or aragest or clinofem or clinovir or curretab or cycrin or depo-prodasone or depo-provera or depo-subqprovera or depo prodasone or depo provera or depo subQ provera or depoclinovir or depomedroxyprogesterone or depoprodasone or depopromone or depoprovera or estrofarlutal or farkital or farlutal or gestapolar or gestapuran or gestapuron or hysron h or lutopolar or lutoral farmit or manodepa or medioxyprogesterone acetate or medroxy progesterone acetate or medroxyprogesteronacetate or medroxyprogesterone 17-acetate or medroxyprogesterone 17 acetate or meges or megestron or meprate or methylacetoxyprogesterone or methylpregnone or mpa gyn 5 or nsc 26 386 or nsc 26386 or nsc26386 or oragest or perkitex or perlutex or prodafem or	1829

	prodasone or progen or progevera or prothyra or provera or ralovera or repromap or veramix or veraplex).mp.	
69	(megestrol or megestrole).mp.	526
70	(navelbine or navelbin or vinbine or vinelbine or navelbine or vinorelbine).mp.	1219
71	(leuprorelin or a 43818 or a43818 or abbott 43818 or carcinil or depo lupron or eligard or enanton or ginecrin or leuplin or leuprogel or leuprolid or leupron or lorelin depot or lucrin or lupride or luprolex or lupron or procren depot or procrin or prostap or reliser or tap 144 or tap144 or tapros or trenantone or viadur).mp.	484
72	(taxane derivatives or taxan*).mp.	1677
73	(trastuzumab emtansine or tdm1 or tdm 1 or tdm?1 or t dm 1 or trastuzumab emtansine).mp.	129
74	exp Aromatase Inhibitors/	567
75	or/14-74	187614
76	4 and 8 and 12 and 13 and 75	1613
77	limit 76 to yr="2016 -Current" [Limit not valid in DARE; records were retained]	670
