# Supplementary material for: Evaluating the clinical effectiveness and safety of various HER2-targeted regimens after prior taxane/trastuzumab in patients with previously treated, unresectable, or metastatic HER2-positive breast cancer: a systematic review and network meta-analysis
Source: Breast Cancer Res Treat. 2020 Feb 25;180(3):597–609. doi: 10.1007/s10549-020-05577-7 (PMC7103014; doi:10.1007/s10549-020-05577-7)
Supplement: Supplementary file 2 — Supplementary file2 (PDF 369 kb) [file 10549_2020_5577_MOESM2_ESM.pdf]

SUPPLEMENTARY APPENDICES

Evaluating the clinical effectiveness and safety of various HER2-targeted regimens after prior taxane/trastuzumab in patients with previously treated, unresectable, or metastatic HER2-positive breast cancer: a systematic review and network meta-analysis

Authors:

Noman Paracha, Adriana Reyes, Véronique Diéras, Ian Krop, Xavier Pivot, Ander Urruticoechea

Corresponding author:

Noman Paracha

F. Hoffmann-La Roche AG
Grenzacherstrasse 124
4070 Basel
Switzerland

Tel: +41 61 688 2661

Email: noman.paracha@roche.com

Online Resource 2: Appendix 2. Eligibility criteria

Criteria	Inclusion criteria	Rationale
**Inclusion criteria**	**Population**<br>• Age: adults (≥ 18 years old)<br>• Sex: any<br>• Race: any<br>• Disease: HER2-positive LABC or mBC<br>• Line of therapy: progression during or after most recent treatment for LABC (with previous treatment with trastuzumab and a taxane) or mBC, or within 6 months after treatment for early-stage disease<br>• Early BC studies may contain fast-relapsing populations that may be of interest<br>• Studies detailing first-line treatments should be included and tagged in the first instance for later scrutiny and potential exclusion	The patient population was restricted to align with the licensed indication for Kadcyla in the treatment of HER2-positive, unresectable LABC or mBC
	**Intervention – not pre-specified, or limited to, but could have included:**<br>• trastuzumab emtansine<br>• bevacizumab<br>• capecitabine<br>• lapatinib<br>• neratinib<br>• pertuzumab<br>• sunitinib<br>• tamoxifen<br>• toremifene<br>• trastuzumab<br>• vinorelbine<br>• any other hormonal, biological or chemotherapeutic agent<br><br>• Combination interventions will also be included	All pharmacological interventions for treatment of HER2-positive, unresectable LABC or mBC (not pre-specified or limited *a priori*)
	**Comparator – not pre-specified, or limited to, but could have included:**<br>• any of the included interventions<br>• placebo<br>• best supportive care[a]<br>• any chemotherapy/immunotherapy	These comparators have been selected potentially to enable both direct and indirect comparisons between the interventions of interest (not pre-specified or limited *a priori*)

	**Study design**<br>• RCTs and non-RCTs with any blinding status<br>• Prospective clinical trials, RCTs and non-RCTs with any blinding status and open-label design, evaluating effectiveness and safety of trastuzumab emtansine (i.e. reporting overall response rates, progression free survival, overall survival and adverse events)<br>• Retrospective studies will only be included if they examine relevant treatments/outcomes	RCTs are the gold standard of clinical evidence, minimizing the risk of confounding and allowing comparison of the relative efficacy of interventions<br><br>To enhance completeness, non-RCTs and open-label studies will also be searched for
	**Phases of trial**<br>• All phases	All phases of trials evaluating pharmacological interventions will be included to avoid missing any potential studies
	**Language restrictions**<br>• Include English-language articles only	The inclusion of English language articles only will not limit results substantially, because a high proportion of articles are written in English
	**Date restrictions**<br>• 1 January 1998 to present	
**Exclusion criteria**	**Intervention**<br>• Studies reporting neo-adjuvant therapies<br><br>**Study design**<br>• Single-arm studies<br>• Clinical registry studies should be excluded<br>• Ongoing studies and study protocols without reported data; efforts will be made to obtain data from any such studies that are supported by Roche<br><br>**No subgroup analysis**<br>• No subgroup analysis for disease of interest<br>• No subgroup analysis for disease stage of interest<br>• No subgroup analysis for previously treated patients	Studies with no subgroup data for the disease, disease stage or previous treatment will not be included, because they would introduce heterogeneity into the review

[a]Best supportive care definition includes (though not restricted to): observation alone; non-chemotherapy drugs; palliative care; and even radiotherapy

BC breast cancer, *HER2* human epidermal growth factor receptor 2, *LABC* locally advanced breast cancer, *mBC* metastatic breast cancer, *RCT* randomized controlled trial, *SR* systematic review
