# Supplementary material for: Evaluating the clinical effectiveness and safety of various HER2-targeted regimens after prior taxane/trastuzumab in patients with previously treated, unresectable, or metastatic HER2-positive breast cancer: a systematic review and network meta-analysis
Source: Breast Cancer Res Treat. 2020 Feb 25;180(3):597–609. doi: 10.1007/s10549-020-05577-7 (PMC7103014; doi:10.1007/s10549-020-05577-7)
Supplement: Supplementary file 3 — Supplementary file3 (PDF 359 kb) [file 10549_2020_5577_MOESM3_ESM.pdf]

SUPPLEMENTARY APPENDICES

Evaluating the clinical effectiveness and safety of various HER2-targeted regimens after prior taxane/trastuzumab in patients with previously treated, unresectable, or metastatic HER2-positive breast cancer: a systematic review and network meta-analysis

Authors:

Noman Paracha, Adriana Reyes, Véronique Diéras, Ian Krop, Xavier Pivot, Ander Urruticoechea

Corresponding author:

Noman Paracha

F. Hoffmann-La Roche AG
Grenzacherstrasse 124
4070 Basel
Switzerland

Tel: +41 61 688 2661

Email: noman.paracha@roche.com

Online Resource 3: Appendix 3. Critical appraisal of relevant studies

**Study: NCT00829166 [EMILIA]**			
**Criterion**	**Description**	**Risk bias**	**Support for judgment**
**Method of randomization**	"randomized…"	Low	"via a hierarchical, dynamic randomisation assignment procedure" (p. 3)
**Allocation concealment**	Central allocation	Low	"an interactive voice response system" (p. 3)
**Baseline comparability**	Comparable	Low	"patient baseline characteristics were well balanced between treatment groups" (Table 1; p. 6)
**Blinding:**<br>• Blinding of participants and personnel	"Open label"	High	Study participants and personnel were not blinded from knowledge of which intervention a participant received
• Blinding of outcomes assessors	"The secondary endpoints of this study were investigator…assessed"; "investigator assessed disease progression"	Unclear	There is a degree of uncertainty about the effects of a lack of blinding of the outcomes assessors some of whom were study personnel.
**Follow-up**	CONSORT Flow Diagram	Low	"All patients were followed up for survival in the clinic or by phone until death" (p. 4) and flow diagram (Fig. 1; p. 5)
**Selective reporting**	Two full text publications but cover OS and Safety i.e. adverse events and co-primary endpoints PFS and ORR	Low	The prespecified outcomes in the clinicaltrials.gov protocol and the methods section appear to have been fully reported albeit separately across the two publications
**Analysis**	ITT at 2nd Interim Analysis	Low	ITT population used for the Overall Survival and the safety population (496 v 495) 2nd Interim Analysis. PFS (496 v 495)
**Other source of bias**		Low	The study appeared to be free from other sources of bias
**Study: NCT00148876 [GBG 26]**			
**Criterion**	**Description**	**Risk bias**	**Support for judgment**
**Method of randomization**	Randomized, open-label study	Low	"For each stratum a randomization list was prepared beforehand; a block permutation method was used with a block size of 4" (p. 22)

**Allocation concealment**	Central allocation	Low	"GBG assigned a randomization number to each participant according to the randomization lists provided by CRS" (p. 26)
**Baseline comparability**	Comparable	Low	"No difference was observed between the two arms in terms of other baseline characteristics" (p. 51)
**Blinding**<br>• Blinding of participants and personnel<br><br>• Blinding of outcomes assessors	"Non-blinded"	High<br><br>High	Study participants and personnel were not blinded from knowledge of which intervention a participant received<br><br>Study personnel were the outcomes assessors, and objective as well as potentially subjective clinical outcomes were included
**Follow-up**	CONSORT Flow Diagram	Low	Flow diagram (Fig. 1; p. 47) All patients accounted for [NB Trial ended prematurely]
**Selective reporting**	Primary and secondary endpoints clearly defined in methods section	Low	All prespecified outcomes in the Methods section appear to have been reported
**Analysis**		Low	Low number of patients post randomization who didn't receive treatment (5/156), moderate but balanced number of patients who discontinued or provided no data (28%) but combined with an ITT analysis (151) for both safety and efficacy outcomes
**Other source of bias**		Low	The study appeared to be free from other sources of bias
**Study: NCT00078572 Cameron et al. and *Geyer et al. 2006* [EGF100151]**			
**Criterion**	**Description**	**Risk bias**	**Support for judgment**
**Method of randomization**	Phase III randomized comparison	Unclear	Reference is made in *Cameron et al. 2008* to an earlier report of this study *Geyer et al. 2006* https://www.nejm.org/doi/full/10.1056/nejmoa064320 Nothing further reported other than "randomly assigned in a 1:1 ratio…permuted blocks of six women"
**Allocation concealment**	Methods of allocation concealment was not reported	Unclear	The method used to conceal the allocation sequence, that is to determine whether intervention allocations could have been foreseen in advance of, or during enrolment, was not reported
**Baseline comparability**	Comparable	Low	"The demographic characteristics of the two groups were similar" (Table 1; p. 535)
**Blinding**			

• Blinding of participants and personnel<br><br>• Blinding of outcomes assessors	“Open-label”<br><br>Outcome assessor was reported to be blinded for TTP and response outcomes	High<br><br>Unclear	Open label study, investigators/study personnel not blinded from knowledge of which intervention a participant received<br><br>*Geyer et al. 2006* states “For analyses of the TTP, PFS, the ORR, and the clinical benefit rate, copies of serial radiographs and photographs of visible lesions used for efficacy determinations were collected for independent assessment under blinded conditions. Supportive analyses of these end points were conducted with the use of investigator-reported assessments”<br>Nothing further specified regarding the “under blinded conditions” methods, but the study included “investigator-reported assessments”, resulting in a degree of uncertainty in the risk of bias
**Follow-up**	CONSORT Flow Diagram	Low	Flow diagram (Fig. 1; p. 536) All patients accounted for
**Selective reporting**	Primary and secondary endpoints clearly defined in Methods section	Low	All prespecified outcomes in the Methods section appear to have been reported
**Analysis**	ITT	Low	Moderately balanced number of; post randomization patients who didn’t receive treatment or received incorrect therapy and data analysed using ITT population. [Also, in *Geyer et al. 2006*] Low risk of bias
**Other source of bias**		Low	The study appeared to be free from other sources of bias
**Study: NCT [Martin et al.] NCT00777101**			
**Criterion**	**Description**	**Risk bias**	**Support for judgement**
**Method of randomization**	Phase II randomised trial	Unclear	“Randomised 1:1 to treatment…” (p. 3765) Nothing further reported
**Allocation concealment**	Method of allocation concealment not reported	Unclear	The method used to conceal the allocation sequence, that is to determine whether intervention allocations could have been foreseen in advance of, or during enrolment, was not reported
**Baseline comparability**	Comparable	Unclear	“Demographic and baseline disease characteristics were balanced between treatment arms” (Table 1; p. 3766)
**Blinding**<br>• Blinding of participants and personnel	Open label	High	Study participants and personnel did not appear to be blinded from knowledge of which intervention a participant received

• Blinding of outcomes assessors		High	Nothing specified but clinical outcome assessments appear to have been made by investigators
**Follow-up**	CONSORT Flow Diagram	Low	Flow diagram (Fig. 1; p. 3767) All patients accounted for
**Selective reporting**	Primary and secondary endpoints clearly defined in Methods section	Low	All prespecified outcomes in the Methods section appear to have been reported
**Analysis**	ITT	Low	ITT population used for all analyses
**Other source of bias**		Low	The study appeared to be free from other sources of bias
**Study: NCT00820222 [CEREBEL]**			
**Criterion**	**Description**	**Risk bias**	**Support for judgment**
**Method of randomization**	Phase III randomized study	Unclear	"Patients were randomly assigned 1:1…" Permuted block design…with blocks of size 4 within each stratum…" (p. 1564)<br>Nothing further reported
**Allocation concealment**	Method of allocation concealment not reported	Unclear	The method used to conceal the allocation sequence, that is to determine whether intervention allocations could have been foreseen in advance of, or during enrolment, was not reported
**Baseline comparability**	Comparable	Low	The trialists report reasonable attempts to ensure baseline balance between intervention groups which include stratified randomization and a permuted block design (4) per stratum
**Blinding**<br>• Blinding of participants and personnel<br><br>• Blinding of outcomes assessors	Open label	High<br><br>Unclear	Study participants and personnel did not appear to be blinded from knowledge of which intervention a participant received<br><br>Primary endpoint incidence of CNS (as site of first relapse) was based on IRC; secondary endpoints were investigator assessed and potentially 'unblinded'
**Follow-up**	CONSORT Flow Diagram	Low	Flow diagram (Fig. 1; p. 1565) All patients accounted for with balanced losses to follow-up per treatment arm<br>[NB study terminated early at 3 years]
**Selective reporting**	Primary and secondary endpoints clearly defined in Methods section	Low	All prespecified outcomes in the NCT study protocol and Methods section appear to have been reported

**Analysis**	Unclear	Unclear[a]	Primary and secondary efficacy analysis based on M-ITT population. (Table 2; p. 1567)
**Other source of bias**	Study was underpowered for the primary endpoint because it was terminated early based on recommendation of the IDMC, after analysis of interim safety and efficacy data	High	Statistical analysis biased due to early study termination at 540 patients (650 patients [325/arm] required to achieve 80% power)
**Study: NCT01026142 [PHEREXA]**			
**Criterion**	**Description**	**Risk bias**	**Support for judgment**
**Method of randomization**	Randomized phase III study	Low	"with a dynamic randomization list…" (p. 3031)
**Allocation concealment**	Central allocation	Low	"were randomly assigned 1:1 via interactive voice response system and/or interactive web-based response system" (p. 3031)
**Baseline comparability**	Comparable	Low	"Demographics and baseline characteristics were generally similar with a slight imbalance in the number of patients from Asia" (p. 3032)<br>Imbalance between groups in Asia cohort but represent a relatively low % of total number of participants
**Blinding**<br>• Blinding of participants and personnel<br><br>• Blinding of outcomes assessors	Open label	High<br><br><br>Unclear	Open label study, investigators/study personnel not blinded from knowledge of which intervention a participant received<br><br>Open label study, some investigators/study personnel assessed outcomes ("secondary objectives") and an IRC assessed outcome ("primary objective"), but these were largely objective and did not include any patient assessed outcomes.<br>There is a degree of uncertainty about the effects of a lack of blinding of the outcomes assessors some of whom were study personnel
**Follow-up**	CONSORT Flow Diagram	Low	Flow diagram (Fig 1; p. 3032) All patients accounted for

**Selective reporting**	Primary and secondary objectives clearly defined in Methods section	Low risk	All prespecified outcomes in the Methods section appear to have been reported
**Analysis**	ITT analysis for key outcomes	Low	Low number of post randomization patients who didn't receive treatment (6/446) excluded from Safety population.<br>PFS/OS assessed in ITT population.<br>Low risk of bias
**Other source of bias**		Low	The study appeared to be free from other sources of bias
**Study: NCT [ELTOP]**			
**Criterion**	**Description**	**Risk bias**	**Support for judgment**
**Method of randomization**	Randomized phase II trial	Unclear	"randomly assigned" (p. 67)<br>Nothing further reported
**Allocation concealment**	Method of allocation concealment not reported	Unclear	The method used to conceal the allocation sequence, that is to determine whether intervention allocations could have been foreseen in advance of, or during enrolment, was not reported
**Baseline comparability**	Comparable	Low	"Patient characteristics were balanced between the 2 arms" (Table 1; p. 69). The trialists report reasonable attempts to ensure baseline balance between intervention groups including stratified randomization
**Blinding**<br>• Blinding of participants and personnel<br>• Blinding of outcomes assessors	Open label	High<br><br>High	Study participants and personnel did not appear to be blinded from knowledge of which intervention a participant received<br><br>The report did not confirm who were the outcome assessors nor any method used to achieve their blinding
**Follow-up**	CONSORT Flow Diagram	Low	Flow diagram (Fig. 1; p. 69) All patients accounted for
**Selective reporting**	Primary and secondary endpoints clearly defined in Methods section	Low	All prespecified outcomes in the Methods section appear to have been reported
**Analysis**	ITT	Low	ITT analysis used for efficacy and safety
**Other source of bias**		Low	The study appeared to be free from other sources of bias

[a]Unclear risk due to non-disclosure of how study accounted for missing data
