# Supplementary material for: Evaluating the clinical effectiveness and safety of various HER2-targeted regimens after prior taxane/trastuzumab in patients with previously treated, unresectable, or metastatic HER2-positive breast cancer: a systematic review and network meta-analysis
Source: Breast Cancer Res Treat. 2020 Feb 25;180(3):597–609. doi: 10.1007/s10549-020-05577-7 (PMC7103014; doi:10.1007/s10549-020-05577-7)
Supplement: Supplementary file 4 — Supplementary file4 (PDF 343 kb) [file 10549_2020_5577_MOESM4_ESM.pdf]

SUPPLEMENTARY APPENDICES

Evaluating the clinical effectiveness and safety of various HER2-targeted regimens after prior taxane/trastuzumab in patients with previously treated, unresectable, or metastatic HER2-positive breast cancer: a systematic review and network meta-analysis

Authors:

Noman Paracha, Adriana Reyes, Véronique Diéras, Ian Krop, Xavier Pivot, Ander Urruticoechea

Corresponding author:

Noman Paracha

F. Hoffmann-La Roche AG
Grenzacherstrasse 124
4070 Basel
Switzerland

Tel: +41 61 688 2661

Email: noman.paracha@roche.com

Online Resource 4: Appendix 4. Statistical methodology

- Bayesian analysis was conducted using the GeMTC R package in the Roche Biometrics Experimental Environment (BEE) using R version 3.4.4 [1, 2]; hazard ratios (HRs) were estimated using both fixed- and random-effects models.
  - Prior distribution for the log HRs was specified as d ~ N(0,1000)) for progression-free survival (PFS) and d ~ N(0,100) for overall survival (OS) [43].
  - The between-study variance could not be accurately estimated owing to the small number of studies available per comparison; informative priors were used instead.
  - The prior of the variance of the random effects are endpoint-dependent.
    - OS: tau2~LN(-4.27, 1.48^2).
    - PFS: tau2~LN(-3.23, 1.35^2).
    - Overall response rate: tau~u(0,0.1).
    - A set of informative priors was developed [3] for the between-study variance (tau^2) for binomial outcomes based on a study of almost 15 000 meta-analyses. They report as prior for tau^2 ~ log normal (−2.10,1.58^2) for analysis of adverse events comparing pharmacological to pharmacological interventions.
  - For most of the analysis:
    - burn in of 5000 of a total of 90 000 iterations.
  - When convergence was not achieved:
    - burn in of 50 000 of a total of 900 000 iterations.
  - And when convergence was still not achieved:
    - burn in of 500 000 of a total of 9 000 000 iterations.
  - Three parallel chains were run, and the Markov Chain Monte Carlo (MCMC) convergence was assessed inspecting trace plots and the Brooks–Gelman–Rubin statistics (Rhat). Sufficient numbers of iterations were used to achieve effective sample sizes (n.eff) allowing for posterior inference (n.eff > 1000).
- Treatment crossover–adjusted OS estimates were used in a sensitivity analysis where applicable.

References

1. van Valkenhoef G, Kuiper J (2009) gemtc: network meta-analysis using Bayesian methods. R package version 0.8-2. 2016. Available at: https://CRAN.R-project.org/package=gemtc. Accessed 09 April 2019

2. R Core Team (2009) R: A language and environment for statistical computing. R Foundation for Statistical Computing, Vienna, Austria. 2018. Available at: https://www.R-project.org/. Accessed 09 April 2019

3. Turner RM, Jackson D, Wei Y, Thompson SG, Higgins JPT (2015) Predictive distributions for between-study heterogeneity and simple methods for their application in Bayesian meta-analysis. Stat Med 2015;34(6):984–998
