# Supplementary material for: Evaluating the clinical effectiveness and safety of various HER2-targeted regimens after prior taxane/trastuzumab in patients with previously treated, unresectable, or metastatic HER2-positive breast cancer: a systematic review and network meta-analysis
Source: Breast Cancer Res Treat. 2020 Feb 25;180(3):597–609. doi: 10.1007/s10549-020-05577-7 (PMC7103014; doi:10.1007/s10549-020-05577-7)
Supplement: Supplementary file 5 — Supplementary file5 (PDF 389 kb) [file 10549_2020_5577_MOESM5_ESM.pdf]

# SUPPLEMENTARY APPENDICES

## Evaluating the clinical effectiveness and safety of various HER2-targeted regimens after prior taxane/trastuzumab in patients with previously treated, unresectable, or metastatic HER2-positive breast cancer: a systematic review and network meta-analysis

**Authors:**

Noman Paracha, Adriana Reyes, Véronique Diéras, Ian Krop, Xavier Pivot, Ander Urruticoechea

**Corresponding author**:

Noman Paracha

F. Hoffmann-La Roche AG
Grenzacherstrasse 124
4070 Basel
Switzerland

Tel: +41 61 688 2661

Email: noman.paracha@roche.com

# Online Resource 5: Appendix 5. Data inputs for NMA

## Data inputs – PFS and OS

| Study name | Treatment 1 | Treatment 2 | PFS HR (95% CI) Treatment 1 vs 2 | OS HR (95% CI) Treatment 1 vs 2 | XO Adj. OS HR (95% CI) Treatment 1 vs 2 |
|---|---|---|---|---|---|
| EMILIA [43] | T-DM1 | LapCap | 0.65 (0.55, 0.77) | 0.75 (0.64, 0.88) | 0.69[a] (0.58, 0.85) |
| EGF100151 [36] | LapCap | Cap alone | 0.55 (0.40, 0.74) | 0.87 (0.70, 1.08) | 0.80[b] (0.64, 0.99) |
| GBG 26 [32] | TrasCap | Cap alone | 0.68 (0.48, 0.96) | 0.94 (0.65, 1.35) | NR |
| Martin et al., 2013 [39] | Neratinib | LapCap | 1.19 (0.89, 1.60) | 1.25 (0.83, 1.86) | NR |
| CEREBEL [17] | LapCap | TrasCap | 1.13 (0.85, 1.50) | 1.18 (0.76, 1.18) | NR |
| PHEREXA [26] | PerTrasCap | TrasCap | 0.83 (0.68, 1.02) | 0.76 (0.60, 0.98) | NR |
| ELTOP [40] | LapCap | TrasCap | 0.81 (0.55, 1.21) | 0.58 (0.26, 1.31) | NR |

[a]Using RPSFTM – see NICE appraisal [43]

[b]XO as time dependent covariate; a subsequent follow-up analysis determined that HR for OS in EMILIA allowing for treatment crossover was 0.75 (95% CI: 0.64, 0.88) [13]

*Cap* capecitabine, *CI* confidence interval, *HR* hazard ratio, *LapCap* lapatinib plus capecitabine, *NR* not reported, *OS* overall survival, *PFS* progression-free survival, *PerTrasCap* pertuzumab plus trastuzumab plus capecitabine, *T-DM1* trastuzumab emtansine, *TrasCap* trastuzumab plus capecitabine

**Data inputs – ORR**

| Study name[a] | Treatment | Event (r) | Evaluable (n) | % |
|---|---|---|---|---|
| EMILIA [10] | T-DM1 | 173 | 397 | 44% |
| EMILIA [10] | LapCap | 120 | 389 | 31% |
| EGF100151 [38] | LapCap | 47 | 198 | 24% |
| EGF100151 [38] | Cap | 28 | 201 | 14% |
| GBG 26 [32] | TrasCap | 37 | 77 | 48% |
| GBG 26 [32] | Cap | 20 | 74 | 27% |
| Martin et al., 2013 [39] | Neratinib | 34 | 117 | 29% |
| Martin et al., 2013 [39] | LapCap | 47 | 116 | 41% |
| PHEREXA [26] | PerTrasCap | 73 | 163 | 45% |
| PHEREXA [26] | TrasCap | 61 | 164 | 37% |
| ELTOP [40] | TrasCap | 16 | 40 | 40% |
| ELTOP [40] | LapCap | 15 | 37 | 41% |

[a]Data for CEREBEL not available

*Cap* capecitabine, *LapCap* lapatinib plus capecitabine, *ORR* overall response rate, *PerTrasCap* pertuzumab plus trastuzumab plus capecitabine, *T-DM1* trastuzumab emtansine, *TrasCap* trastuzumab plus capecitabine

## Data inputs – adverse events

| Study name[a] | Treatment | Evaluable (n) | Event (r) | | | | | | | | | | |
|---|---|---|---|---|---|---|---|---|---|---|---|---|---|
| | | | Anemia | Diarrhea | Fatigue | Increased ALT | Increased AST | Mucosal inflammation | Nausea | Neutropenia | PPE | Thrombocytopenia | Vomiting |
| EMILIA [13] | T-DM1 | 490 | 19 | 9 | 12 | 15 | 22 | 1 | 4 | 11 | 0 | 70 | 5 |
| EMILIA [13] | LapCap | 488 | 11 | 103 | 17 | 9 | 7 | 11 | 13 | 21 | 87 | 2 | 24 |
| EGF100151 [38] | LapCap | 198 | 2 | 28 | 6 | 4 | 6 | 0 | 4 | 8 | 24 | 2 | 4 |
| EGF100151 [38] | Cap | 191 | 2 | 19 | 7 | 2 | 4 | 4 | 4 | 6 | 27 | 4 | 4 |
| GBG 26 [32] | TrasCap | 77 | 0 | 12 | 3 | 3 | 5 | 1 | 1 | 4 | 25 | 0 | 1 |
| GBG 26 [32] | Cap | 74 | 2 | 14 | 4 | 2 | 2 | 2 | 3 | 3 | 18 | 1 | 3 |
| Martin et al., 2013 [39] | Neratinib | 116 | NR | 33 | 3 | 4 | 4 | NR | 5 | 2 | 0 | NR | 5 |
| Martin et al., 2013 [39] | LapCap | 115 | NR | 11 | 3 | 1 | 3 | NR | 4 | 4 | 16 | NR | 2 |
| PHEREXA [26] | PerTrasCap | 228 | 3 | 37 | 6 | 3 | NR | 6 | 6 | 9 | 23 | 3 | 4 |
| PHEREXA [26] | TrasCap | 218 | 6 | 22 | 3 | 1 | NR | 7 | 7 | 13 | 48 | 1 | 7 |
| ELTOP [40] | TrasCap | 43 | 0 | 4 | NR | 2 | 1 | NR | NR | 2 | 9 | NR | NR |
| ELTOP [40] | LapCap | 43 | 0 | 7 | NR | 1 | 3 | NR | NR | 1 | 9 | NR | NR |

*ALT* alanine aminotransferase, *AST* aspartate aminotransferase, *Cap* capecitabine, *LapCap* lapatinib plus capecitabine, *NR* not reported, *PerTrasCap* pertuzumab plus trastuzumab plus capecitabine, *PPE* palmar–plantar erythrodysesthesia, *T-DM1* trastuzumab emtansine, *TrasCap* trastuzumab plus capecitabine

| Study name[a] | Treatment | Event (r) | | | |
|---|---|---|---|---|---|
| | | Evaluable (n) | Grade 3 and above | Serious AEs | Tx disc. due to AEs |
| EMILIA [13] | T-DM1 | 490 | 233 | 92 | 50 |
| EMILIA [13] | LapCap | 488 | 291 | 99 | 59 |
| EGF100151 [38] | LapCap | 198 | 12[a] | NR | 28 |
| EGF100151 [38] | Cap | 191 | 12[a] | NR | 27 |
| GBG 26 [32] | TrasCap | 77 | 49 | 13 | 8 |
| GBG 26 [32] | Cap | 74 | 49 | 18 | 10 |
| Martin et al., 2013 [39] | Neratinib | 116 | NR | 31 | 7 |
| Martin et al., 2013 [39] | LapCap | 115 | NR | 25 | 20 |
| PHEREXA [26] | PerTrasCap | 228 | 118 | 56 | 48 |
| PHEREXA [26] | TrasCap | 218 | 130 | 52 | 42 |
| ELTOP [40] | TrasCap | 43 | NR | NR | 5 |
| ELTOP [40] | LapCap | 43 | NR | NR | 12 |

*AE* adverse event, *Cap* capecitabine, *LapCap* lapatinib plus capecitabine, *NR* not reported, *PerTrasCap* pertuzumab plus trastuzumab plus capecitabine, *T-DM1* trastuzumab emtansine, *TrasCap* trastuzumab plus capecitabine

[a]These data are given for grade 4 and above
