# Supplementary material for: Evaluating the clinical effectiveness and safety of various HER2-targeted regimens after prior taxane/trastuzumab in patients with previously treated, unresectable, or metastatic HER2-positive breast cancer: a systematic review and network meta-analysis
Source: Breast Cancer Res Treat. 2020 Feb 25;180(3):597–609. doi: 10.1007/s10549-020-05577-7 (PMC7103014; doi:10.1007/s10549-020-05577-7)
Supplement: Supplementary file 6 — Supplementary file6 (PDF 268 kb) [file 10549_2020_5577_MOESM6_ESM.pdf]

SUPPLEMENTARY APPENDICES

Evaluating the clinical effectiveness and safety of various HER2-targeted regimens after prior taxane/trastuzumab in patients with previously treated, unresectable, or metastatic HER2-positive breast cancer: a systematic review and network meta-analysis

Authors:

Noman Paracha, Adriana Reyes, Véronique Diéras, Ian Krop, Xavier Pivot, Ander Urruticoechea

Corresponding author:

Noman Paracha

F. Hoffmann-La Roche AG
Grenzacherstrasse 124
4070 Basel
Switzerland

Tel: +41 61 688 2661

Email: noman.paracha@roche.com

Online Resource 6: Appendix 6. Treatment network plots for adverse events: (a) adverse events (grade 3 and above); (b) treatment discontinuation due to adverse events; (c) serious adverse events; (d) diarrhea, neutropenia, increased ALT; (e) fatigue, nausea, vomiting; (f) increased AST

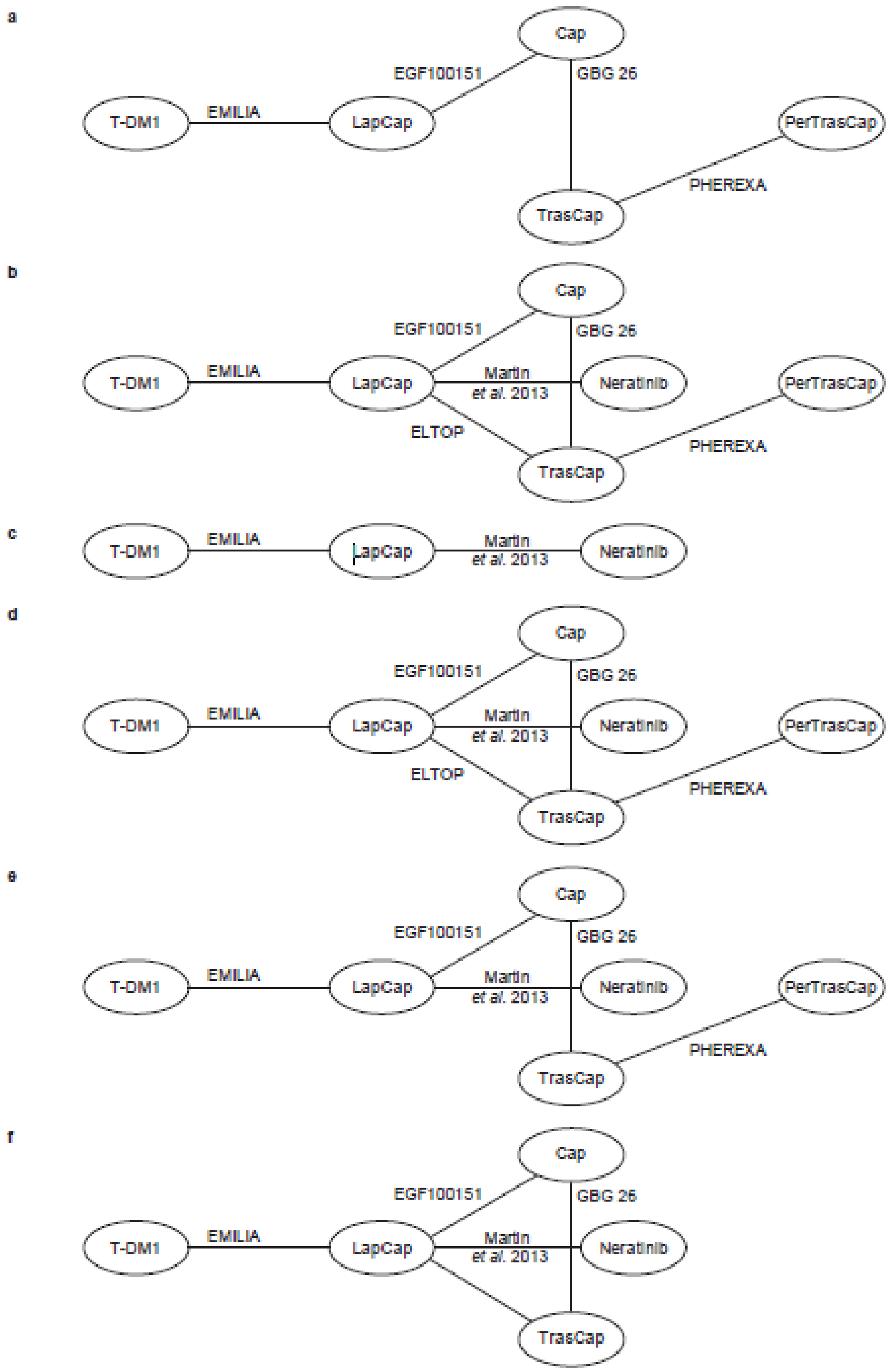

ALT alanine aminotransferase, *AST* aspartate aminotransferase, *Cap* capecitabine, *Lap* lapatinib, *Per* pertuzumab, *T-DM1* trastuzumab emtansine, *Tras* trastuzumab
