# Supplementary material for: Evaluating the clinical effectiveness and safety of various HER2-targeted regimens after prior taxane/trastuzumab in patients with previously treated, unresectable, or metastatic HER2-positive breast cancer: a systematic review and network meta-analysis
Source: Breast Cancer Res Treat. 2020 Feb 25;180(3):597–609. doi: 10.1007/s10549-020-05577-7 (PMC7103014; doi:10.1007/s10549-020-05577-7)
Supplement: Supplementary file 7 — Supplementary file7 (PDF 260 kb) [file 10549_2020_5577_MOESM7_ESM.pdf]

<u>SUPPLEMENTARY APPENDICES</u>

Evaluating the clinical effectiveness and safety of various HER2-targeted regimens after prior taxane/trastuzumab in patients with previously treated, unresectable, or metastatic HER2-positive breast cancer: a systematic review and network meta-analysis

Authors:

Noman Paracha, Adriana Reyes, Véronique Diéras, Ian Krop, Xavier Pivot, Ander Urruticoechea

Corresponding author:

Noman Paracha

F. Hoffmann-La Roche AG
Grenzacherstrasse 124
4070 Basel
Switzerland

Tel: +41 61 688 2661

Email: noman.paracha@roche.com

Online Resource 7: Appendix 7. Odds ratio estimates for anemia with study GBG 26 excluded

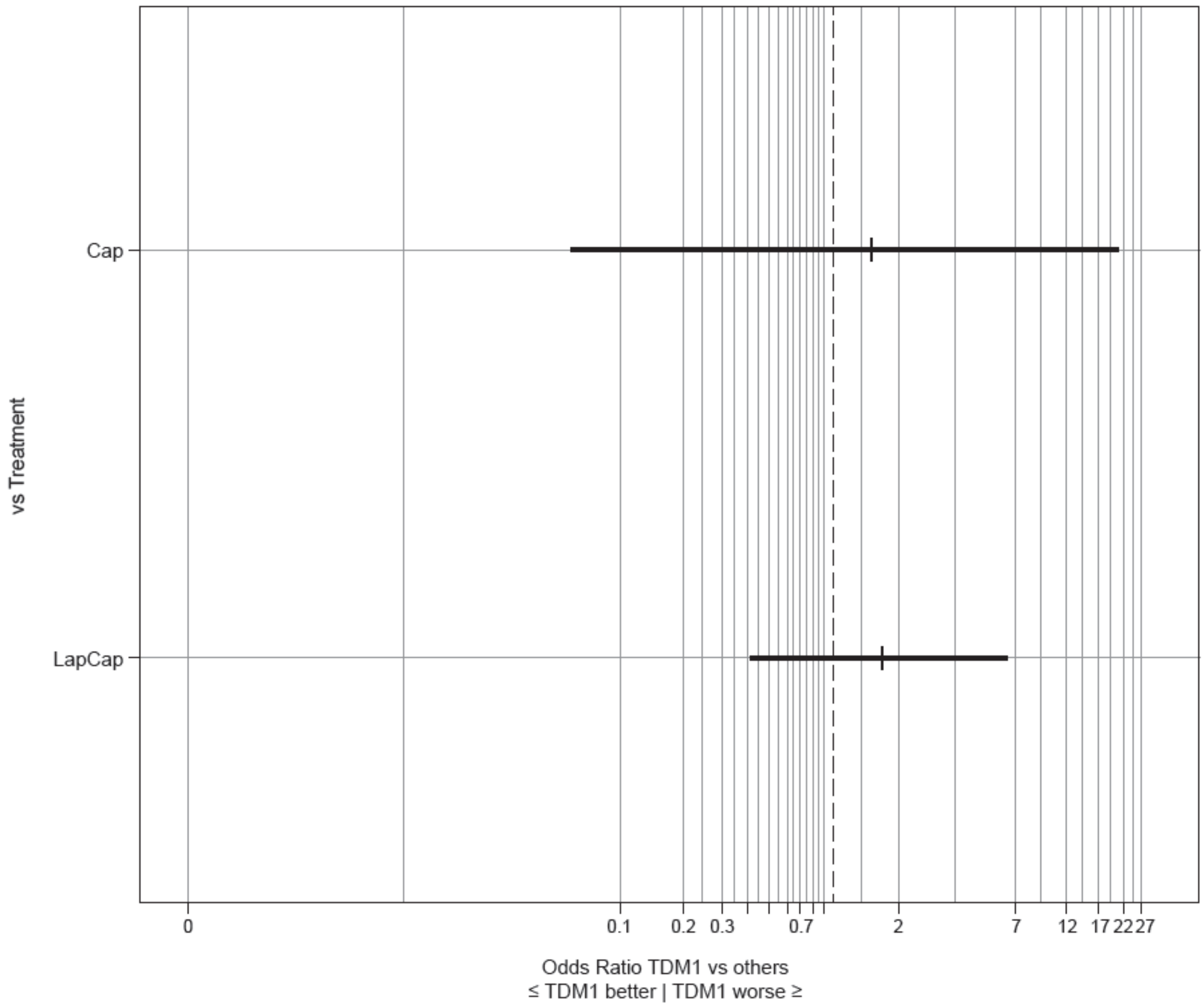

SUCRA percentages: LapCap, 67.6%; Cap, 54.2%; T-DM1, 28.1%

OR (95% CrI) vs T-DM1: Cap, 1.50 (0.06, 20.91); LapCap, 1.68 (0.41, 6.52)

Cap capecitabine, *CrI* credible interval, *Lap* lapatinib, *OR* odds ratio, *SUCRA* Surface Under the Cumulative Ranking Curve, *T-DM1* trastuzumab emtansine
