# Supplementary material for: Evaluating the clinical effectiveness and safety of various HER2-targeted regimens after prior taxane/trastuzumab in patients with previously treated, unresectable, or metastatic HER2-positive breast cancer: a systematic review and network meta-analysis
Source: Breast Cancer Res Treat. 2020 Feb 25;180(3):597–609. doi: 10.1007/s10549-020-05577-7 (PMC7103014; doi:10.1007/s10549-020-05577-7)
Supplement: Supplementary file 8 — Supplementary file8 (PDF 370 kb) [file 10549_2020_5577_MOESM8_ESM.pdf]

SUPPLEMENTARY APPENDICES

Evaluating the clinical effectiveness and safety of various HER2-targeted regimens after prior taxane/trastuzumab in patients with previously treated, unresectable, or metastatic HER2-positive breast cancer: a systematic review and network meta-analysis

Authors:

Noman Paracha, Adriana Reyes, Véronique Diéras, Ian Krop, Xavier Pivot, Ander Urruticoechea

Corresponding author:

Noman Paracha

F. Hoffmann-La Roche AG
Grenzacherstrasse 124
4070 Basel
Switzerland

Tel: +41 61 688 2661

Email: noman.paracha@roche.com

Online Resource 8: Appendix 8. Random-effects model convergence statistics

NMA	Estimate						
	**LapCap – Cap**	**Cap – TrasCap**	**LapCap – Neratinib**	**LapCap – T-DM1**	**LapCap – TrasCap**	**TrasCap – PerTrasCap**	**Standard deviation**
OS	1 (28532)		1 (26964)	1 (26530)	1 (26441)	1 (29485)	1.01 (15665)
OSX	1 (27000)		1 (27037)	1 (27000)	1 (26264)	1 (26240)	1 (20315)
PFS	1 (11041)		1 (13147)	1 (20294)	1 (10982)	1 (17832)	1 (1178)
ORR	1 (16092)		1 (19658)	1 (24980)	1 (12773)	1 (23079)	1 (18640)
Treatment discontinuation	1 (23373)		1 (23506)	1.01 (25206)	1 (19402)	1 (28918)	1.02 (3333)
Discontinuation, grade 3+ AEs	1 (23434)	1 (24392)		1 (30782)		1 (26747)	1.01 (2359)
SAE			1 (28007)	1 (30463)			1.01 (3518)
Anemia	1 (27000)	1 (24742)		1 (26794)			1 (20005)
Diarrhea	1 (22415)		1 (21268)	1 (25399)	1 (17237)	1 (26731)	1 (4376)
Fatigue	1 (20186)	1 (13118)	1 (11709)	1 (24054)		1 (15550)	1.01 (2768)
Increased ALT	1 (26151)		1 (22017)	1 (27000)	1 (25275)	1 (25535)	1 (15246)
Increased AST	1 (20186)	1 (13118)	1 (11709)	1 (24054)		1 (15550)	1.01 (4545)
Mucosal inflammation	1 (26435)	1 (27000)		1 (27351)		1 (27843)	1.01 (19174)
Nausea	1 (14442)	1 (5939)	1 (14415)	1 (19323)		1 (17465)	1.01 (3012)
Neutropenia	1 (13750)		1 (10064)	1 (24024)	1 (8867)	1 (22246)	1 (2580)
Thrombocytopenia	1 (27000)	1 (24270)		1 (27000)		1 (27000)	1 (24227)
Vomiting	1 (27000)	1 (25716)	1 (27000)	1 (26552)		1 (27887)	1.01(11419)

Data are Rhat convergence statistic (effective n). Values of Rhat ≤ 1.02 and effective n ≥ 1000 indicate no evidence of a lack of convergence

AE adverse event, *ALT* alanine aminotransferase, *AST* aspartate aminotransferase, *Cap* capecitabine, *Lap* lapatinib, *ORR* overall response rate, *OS* overall survival, *OSX* OS adjusted for crossover, *Per* pertuzumab; *PFS* progression-free survival, *SAE* serious adverse event, *T-DM1* trastuzumab emtansine, *Tras* trastuzumab
