# Supplementary material for: Evaluating the clinical effectiveness and safety of various HER2-targeted regimens after prior taxane/trastuzumab in patients with previously treated, unresectable, or metastatic HER2-positive breast cancer: a systematic review and network meta-analysis
Source: Breast Cancer Res Treat. 2020 Feb 25;180(3):597–609. doi: 10.1007/s10549-020-05577-7 (PMC7103014; doi:10.1007/s10549-020-05577-7)
Supplement: Supplementary file 9 — Supplementary file9 (PDF 462 kb) [file 10549_2020_5577_MOESM9_ESM.pdf]

SUPPLEMENTARY APPENDICES

Evaluating the clinical effectiveness and safety of various HER2-targeted regimens after prior taxane/trastuzumab in patients with previously treated, unresectable, or metastatic HER2-positive breast cancer: a systematic review and network meta-analysis

Authors:

Noman Paracha, Adriana Reyes, Véronique Diéras, Ian Krop, Xavier Pivot, Ander Urruticoechea

Corresponding author:

Noman Paracha

F. Hoffmann-La Roche AG
Grenzacherstrasse 124
4070 Basel
Switzerland

Tel: +41 61 688 2661

Email: noman.paracha@roche.com

Online Resource 9: Appendix 9. Fixed-effects model results

Cross tabulation of treatment HR (95% CrI) for OS

Drug	Neratinib	Cap	LapCap	TrasCap	T-DM1	PerTrasCap
Neratinib		1.13 (0.74, 1.74)	1.25 (0.85, 1.86)	1.35 (0.88, 2.10)	1.68 (1.11, 2.54)	1.78 (1.08, 2.93)
Cap	0.88 (0.58, 1.36)		1.11 (0.92, 1.35)	1.19 (0.94, 1.53)	1.48 (1.15, 1.94)	1.58 (1.12, 2.20)
LapCap	0.80 (0.54, 1.18)	0.90 (0.74, 1.08)		1.07 (0.89, 1.30)	1.34 (1.14, 1.57)	1.42 (1.03, 1.91)
TrasCap	0.74 (0.48, 1.14)	0.84 (0.65, 1.06)	0.93 (0.77, 1.13)		1.24 (0.97, 1.61)	1.32 (1.02, 1.69)
T-DM1	0.59 (0.39, 0.90)	0.67 (0.52, 0.87)	0.75 (0.64, 0.88)	0.80 (0.62, 1.03)		1.06 (0.73, 1.48)
PerTrasCap	0.56 (0.34, 0.92)	0.63 (0.46, 0.90)	0.71 (0.52, 0.97)	0.76 (0.59, 0.98)	0.94 (0.68, 1.37)	

HR < 1 indicates a better outcome with the drug in column 1 than with the comparator drug (columns 2–7)
Cap capecitabine, *CrI* credible interval, *HR* hazard ratio, *Lap* lapatinib, *OS* overall survival, *Per* pertuzumab, *T-DM1* trastuzumab emtansine, *Tras* trastuzumab

Cross tabulation of treatment HR (95% CrI) for OSX

Drug	Neratinib	Cap	LapCap	TrasCap	PerTrasCap	T-DM1
Neratinib		1.06 (0.68, 1.69)	1.25 (0.82, 1.86)	1.33 (0.83, 2.13)	1.73 (1.03, 2.99)	1.80 (1.13, 2.81)
Cap	0.95 (0.59, 1.48)		1.18 (0.98, 1.43)	1.26 (0.99, 1.60)	1.64 (1.19, 2.30)	1.71 (1.30, 2.25)
LapCap	0.80 (0.54, 1.22)	0.85 (0.70, 1.02)		1.06 (0.88, 1.30)	1.39 (1.01, 1.88)	1.44 (1.18, 1.76)
TrasCap	0.75 (0.47, 1.21)	0.79 (0.63, 1.01)	0.94 (0.77, 1.13)		1.30 (1.02, 1.66)	1.35 (1.04, 1.78)
PerTrasCap	0.58 (0.33, 0.97)	0.61 (0.43, 0.84)	0.72 (0.53, 0.99)	0.77 (0.60, 0.98)		1.03 (0.72, 1.49)
T-DM1	0.55 (0.36, 0.88)	0.59 (0.44, 0.77)	0.69 (0.57, 0.84)	0.74 (0.56, 0.96)	0.97 (0.67, 1.39)	

HR < 1 indicates a better outcome with the drug in column 1 than with the comparator drug (columns 2–7)
Cap capecitabine, *CrI* credible interval, *HR* hazard ratio, *Lap* lapatinib, *OSX* OS adjusted for crossover, *Per* pertuzumab, *T-DM1* trastuzumab emtansine, *Tras* trastuzumab

Cross tabulation of treatment HR (95% CrI) for PFS

Drug	Cap	Neratinib	TrasCap	LapCap	PerTrasCap	T-DM1
Cap		1.42 (0.97, 2.14)	1.64 (1.26, 2.10)	1.69 (1.31, 2.15)	1.97 (1.40, 2.68)	2.61 (1.92, 3.50)
Neratinib	0.70 (0.47, 1.03)		1.15 (0.80, 1.63)	1.18 (0.88, 1.63)	1.39 (0.92, 2.03)	1.81 (1.28, 2.56)
TrasCap	0.61 (0.48, 0.79)	0.87 (0.61, 1.24)		1.03 (0.85, 1.27)	1.21 (0.98, 1.47)	1.59 (1.22, 2.06)
LapCap	0.59 (0.46, 0.76)	0.85 (0.61, 1.13)	0.97 (0.79, 1.18)		1.17 (0.88, 1.53)	1.54 (1.30, 1.83)
PerTrasCap	0.51 (0.37, 0.71)	0.72 (0.49, 1.09)	0.83 (0.68, 1.02)	0.85 (0.66, 1.13)		1.32 (0.96, 1.83)
T-DM1	0.38 (0.29, 0.52)	0.55 (0.39, 0.78)	0.63 (0.48, 0.82)	0.65 (0.55, 0.77)	0.76 (0.55, 1.05)	

HR < 1 indicates a better outcome with the drug in column 1 than with the comparator drug (columns 2–7)

Cap capecitabine, *CrI* credible interval, *HR* hazard ratio, *Lap* lapatinib, *Per* pertuzumab, *PFS* progression-free survival, *T-DM1* trastuzumab emtansine, *Tras* trastuzumab

Cross tabulation of treatment OR (95% CrI) for ORR

Drug	T-DM1	PerTrasCap	TrasCap	LapCap	Neratinib	Cap
T-DM1		1.09 (0.49, 2.38)	1.50 (0.76, 2.95)	1.73 (1.31, 2.35)	2.90 (1.64, 5.39)	3.50 (1.99, 6.20)
PerTrasCap	0.92 (0.42, 2.05)		1.36 (0.88, 2.16)	1.59 (0.75, 3.55)	2.66 (1.05, 7.25)	3.20 (1.59, 6.66)
TrasCap	0.67 (0.34, 1.32)	0.73 (0.46, 1.14)		1.17 (0.65, 2.20)	1.93 (0.88, 4.61)	2.35 (1.37, 4.23)
LapCap	0.58 (0.43, 0.76)	0.63 (0.28, 1.33)	0.86 (0.46, 1.54)		1.67 (1.00, 2.89)	2.01 (1.26, 3.34)
Neratinib	0.34 (0.19, 0.61)	0.38 (0.14, 0.95)	0.52 (0.22, 1.14)	0.60 (0.35, 1.00)		1.21 (0.57, 2.54)
Cap	0.29 (0.16, 0.50)	0.31 (0.15, 0.63)	0.43 (0.24, 0.73)	0.50 (0.30, 0.79)	0.83 (0.39, 1.76)	

OR > 1 indicates a better outcome with the drug in column 1 than with the comparator drug (columns 2–7)

Cap capecitabine, *CrI* credible interval, *Lap* lapatinib, *OR* odds ratio, *ORR* overall response rate, *Per* pertuzumab, *T-DM1* trastuzumab emtansine, *Tras* trastuzumab

Cross tabulation of OR (95% CrI) for discontinuation due to any AE

Drug	PerTrasCap	TrasCap	Cap	LapCap	T-DM1	Neratinib
PerTrasCap		1.16 (0.75, 1.84)	1.47 (0.61, 3.68)	1.78 (0.70, 4.66)	2.15 (0.79, 5.97)	6.05 (1.61, 24.34)
TrasCap	0.86 (0.54, 1.34)		1.27 (0.60, 2.78)	1.52 (0.70, 3.48)	1.84 (0.75, 4.50)	5.15 (1.55, 20.04)
Cap	0.68 (0.27, 1.63)	0.79 (0.36, 1.67)		1.20 (0.70, 2.08)	1.44 (0.75, 2.80)	4.06 (1.44, 13.09)
LapCap	0.56 (0.21, 1.42)	0.66 (0.29, 1.43)	0.83 (0.48, 1.42)		1.21 (0.81, 1.76)	3.36 (1.40, 9.47)
T-DM1	0.47 (0.17, 1.26)	0.54 (0.22, 1.34)	0.69 (0.36, 1.34)	0.83 (0.57, 1.23)		2.78 (1.09, 8.60)
Neratinib	0.17 (0.04, 0.62)	0.19 (0.05, 0.65)	0.25 (0.08, 0.70)	0.30 (0.11, 0.72)	0.36 (0.12, 0.91)	

OR < 1 indicates a better outcome with the drug in column 1 than with the comparator drug (columns 2–7)

AE adverse event, *Cap* capecitabine, *CrI* credible interval, *Lap* lapatinib, *OR* odds ratio, *Per* pertuzumab, *T-DM1* trastuzumab emtansine, *Tras* trastuzumab

Cross tabulation of OR (95% CrI) for discontinuation due to any grade 3+ AEs

Drug	Cap	LapCap	TrasCap	PerTrasCap	T-DM1
Cap		1.07 (0.48, 2.55)	1.13 (0.57, 2.19)	1.49 (0.65, 3.22)	1.73 (0.74, 4.46)
LapCap	0.94 (0.39, 2.07)		1.06 (0.35, 2.96)	1.38 (0.42, 4.26)	1.63 (1.24, 2.10)
TrasCap	0.88 (0.46, 1.77)	0.94 (0.34, 2.87)		1.32 (0.89, 1.92)	1.51 (0.54, 4.68)
PerTrasCap	0.67 (0.31, 1.53)	0.72 (0.23, 2.41)	0.76 (0.52, 1.12)		1.16 (0.37, 4.03)
T-DM1	0.58 (0.22, 1.35)	0.61 (0.48, 0.81)	0.66 (0.21, 1.86)	0.86 (0.25, 2.70)	

OR < 1 indicates a better outcome with the drug in column 1 than with the comparator drug (columns 2–7)

AE adverse event, *Cap* capecitabine, *CrI* credible interval, *Lap* lapatinib, *OR* odds ratio, *Per* pertuzumab, *T-DM1* trastuzumab emtansine, *Tras* trastuzumab

Cross tabulation of OR (95% CrI) for SAEs

Drug	Neratinib	LapCap	T-DM1
Neratinib		1.32 (0.72, 2.45)	1.43 (0.71, 2.91)
LapCap	0.76 (0.41, 1.39)		1.09 (0.79, 1.50)
T-DM1	0.70 (0.34, 1.41)	0.92 (0.67, 1.27)	

OR < 1 indicates a better outcome with the drug in column 1 than with the comparator drug (columns 2–7)

Cap capecitabine, *CrI* credible interval, *Lap* lapatinib, *OR* odds ratio, *Per* pertuzumab, *SAE* serious adverse event, *T-DM1* trastuzumab emtansine, *Tras* trastuzumab
