# Supplementary material for: Evaluating the clinical effectiveness and safety of various HER2-targeted regimens after prior taxane/trastuzumab in patients with previously treated, unresectable, or metastatic HER2-positive breast cancer: a systematic review and network meta-analysis
Source: Breast Cancer Res Treat. 2020 Feb 25;180(3):597–609. doi: 10.1007/s10549-020-05577-7 (PMC7103014; doi:10.1007/s10549-020-05577-7)
Supplement: Supplementary file 10 — Supplementary file10 (PDF 27936 kb) [file 10549_2020_5577_MOESM10_ESM.pdf]

SUPPLEMENTARY APPENDICES

Evaluating the clinical effectiveness and safety of various HER2-targeted regimens after prior taxane/trastuzumab in patients with previously treated, unresectable, or metastatic HER2-positive breast cancer: a systematic review and network meta-analysis

Authors:

Noman Paracha, Adriana Reyes, Véronique Diéras, Ian Krop, Xavier Pivot, Ander Urruticoechea

Corresponding author:

Noman Paracha

F. Hoffmann-La Roche AG
Grenzacherstrasse 124
4070 Basel
Switzerland

Tel: +41 61 688 2661

Email: noman.paracha@roche.com

Online Resource 10: Appendix 10. Random-effects model convergence plots

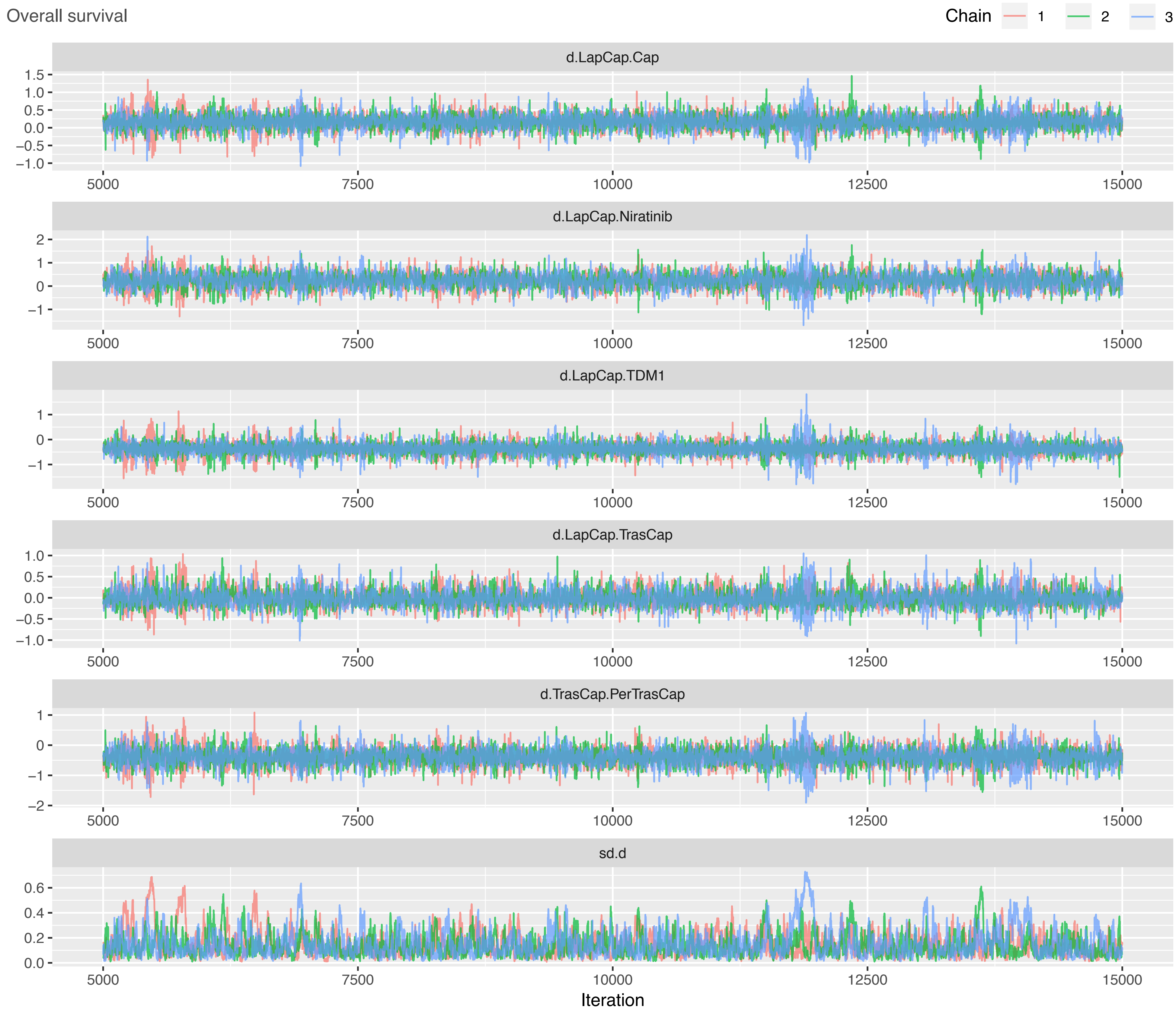
Overall survival
Chain
1
2
3
d.LapCap.Cap
1.5
1.0
0.5
0.0
−0.5
−1.0
5000
7500
10000
12500
15000
d.LapCap.Niratinib
2
1
0
−1
d.LapCap.TDM1
1
0
−1
d.LapCap.TrasCap
1.0
0.5
0.0
−0.5
−1.0
d.TrasCap.PerTrasCap
1
0
−1
−2
sd.d
0.6
0.4
0.2
0.0
Iteration

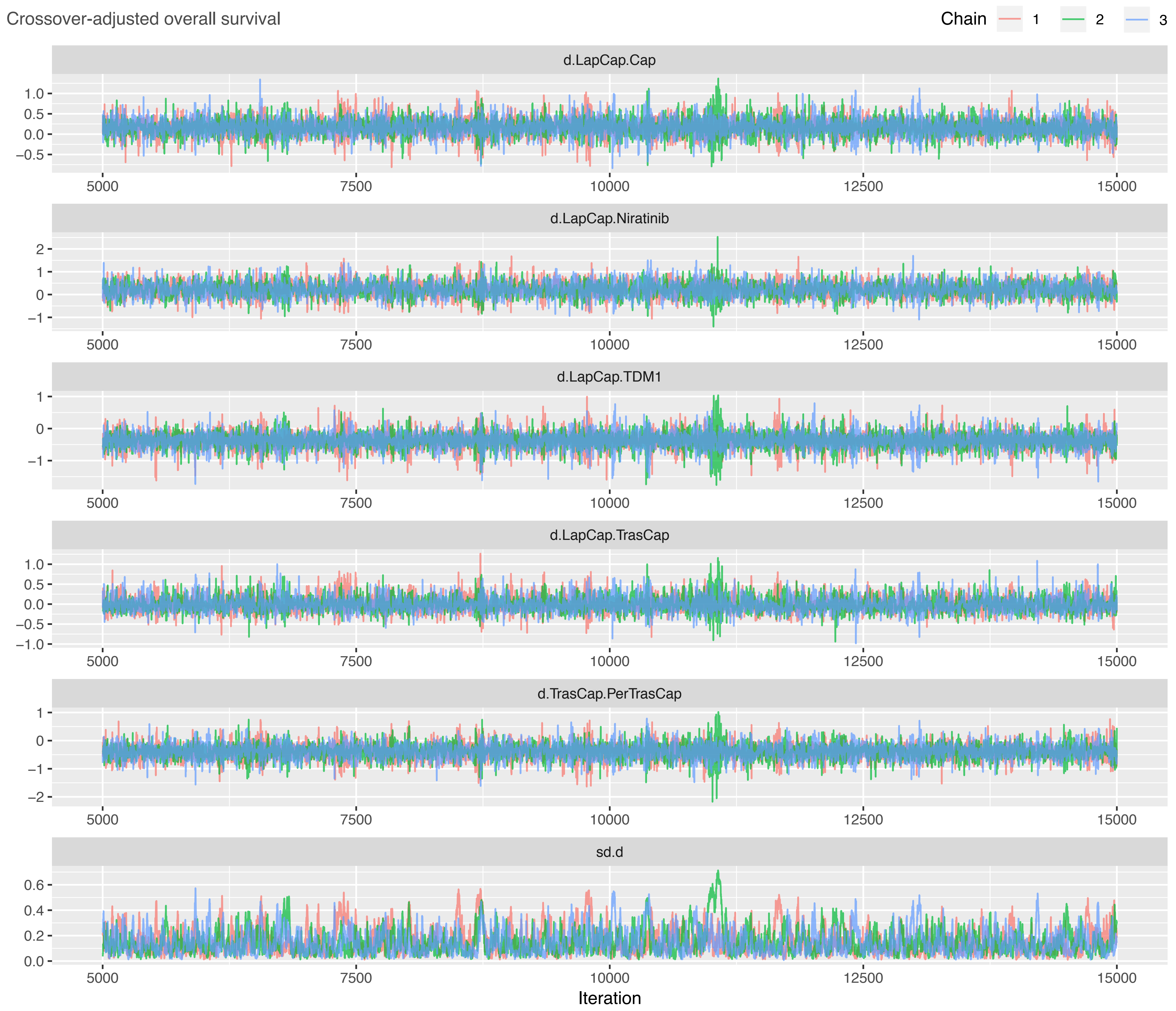

Crossover-adjusted overall survival
Chain 1 2 3
d.LapCap.Cap
d.LapCap.Niratinib
d.LapCap.TDM1
d.LapCap.TrasCap
d.TrasCap.PerTrasCap
sd.d
5000 7500 10000 12500 15000
Iteration

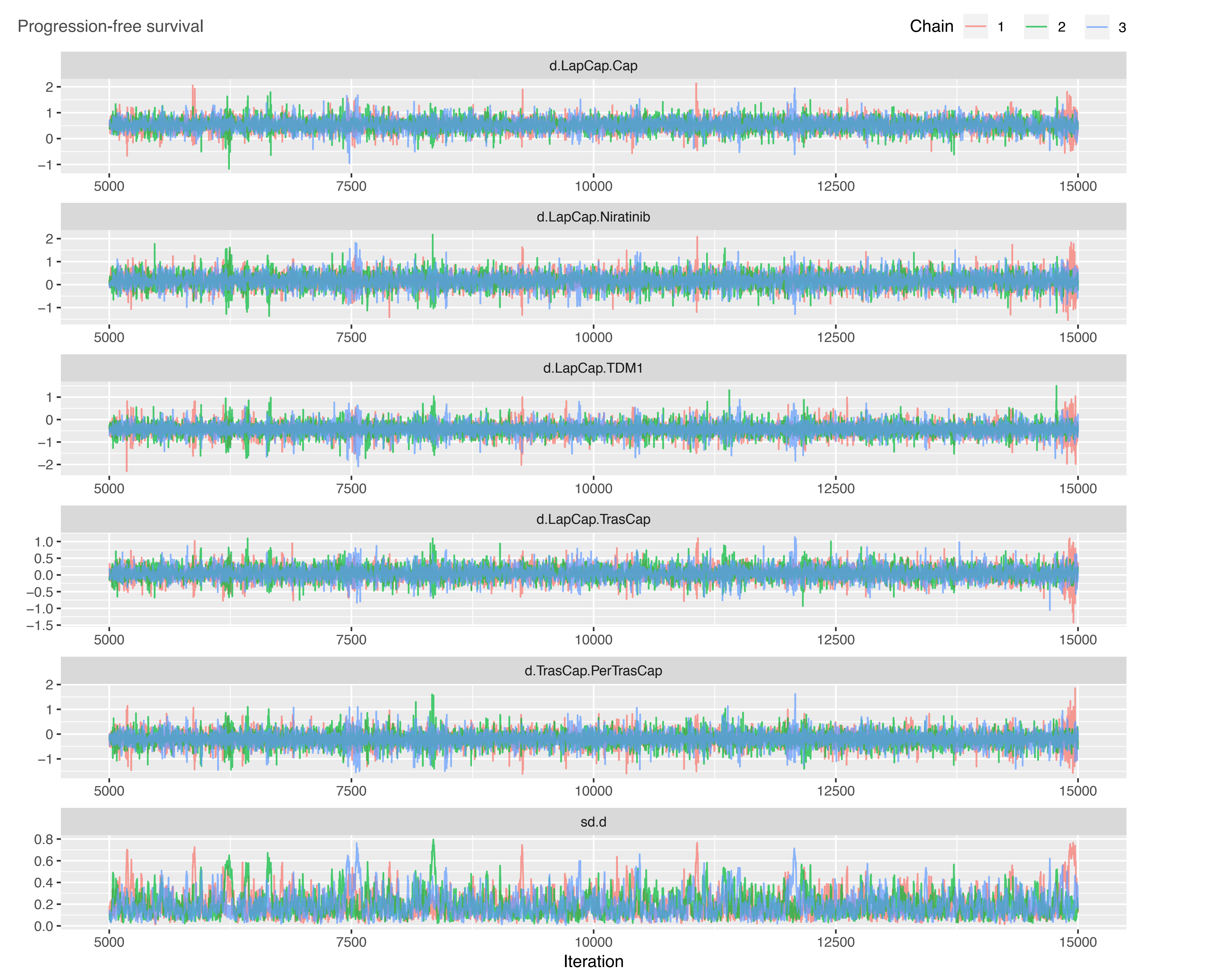
Progression-free survival
Chain
1
2
3
d.LapCap.Cap
d.LapCap.Niratinib
d.LapCap.TDM1
d.LapCap.TrasCap
d.TrasCap.PerTrasCap
sd.d
5000
7500
10000
12500
15000
Iteration

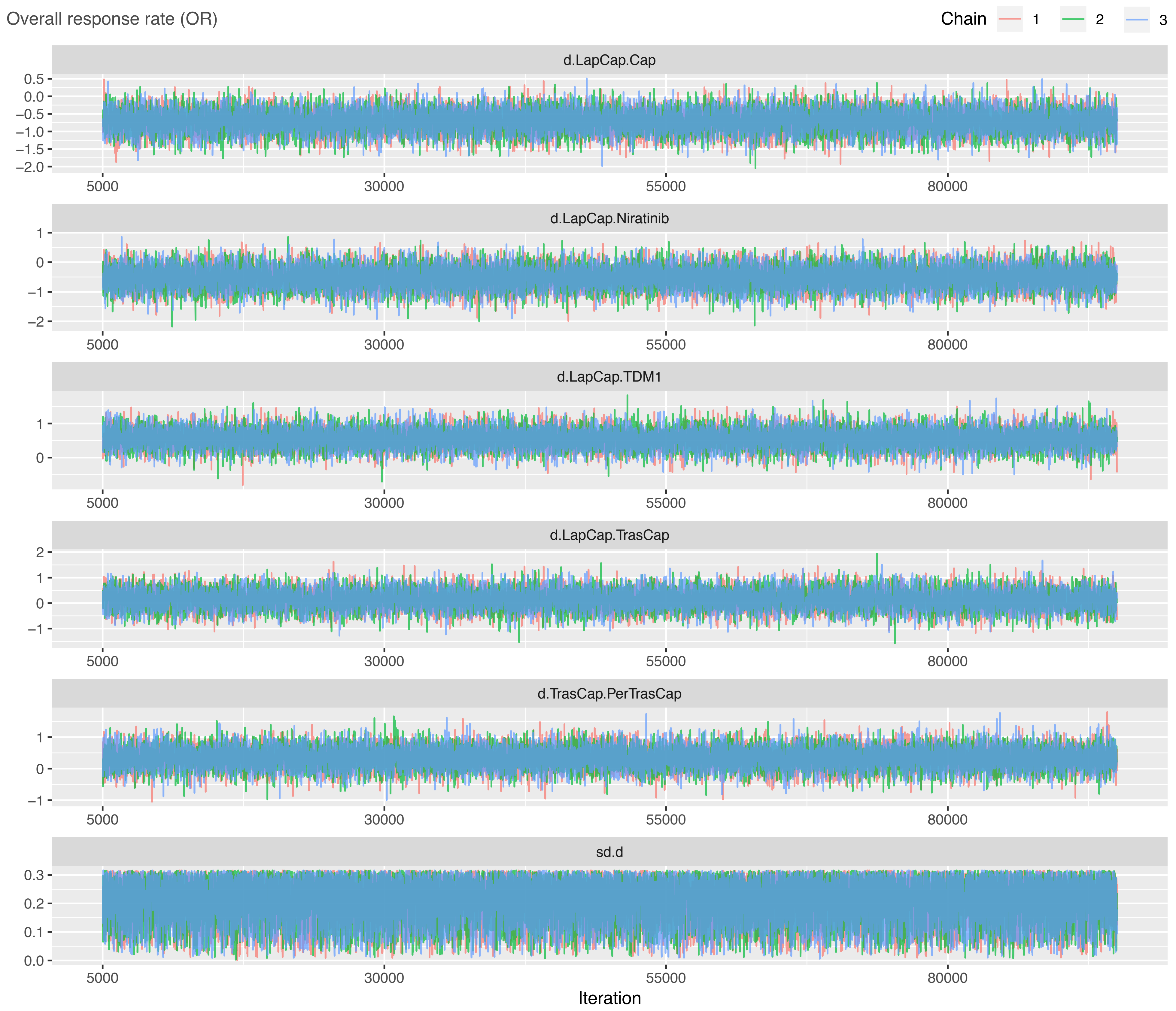

Overall response rate (OR)
Chain
1
2
3
d.LapCap.Cap
d.LapCap.Niratinib
d.LapCap.TDM1
d.LapCap.TrasCap
d.TrasCap.PerTrasCap
sd.d
0.5
0.0
−0.5
−1.0
−1.5
−2.0
1
0
−1
−2
2
0.3
0.2
0.1
5000
30000
55000
80000
Iteration

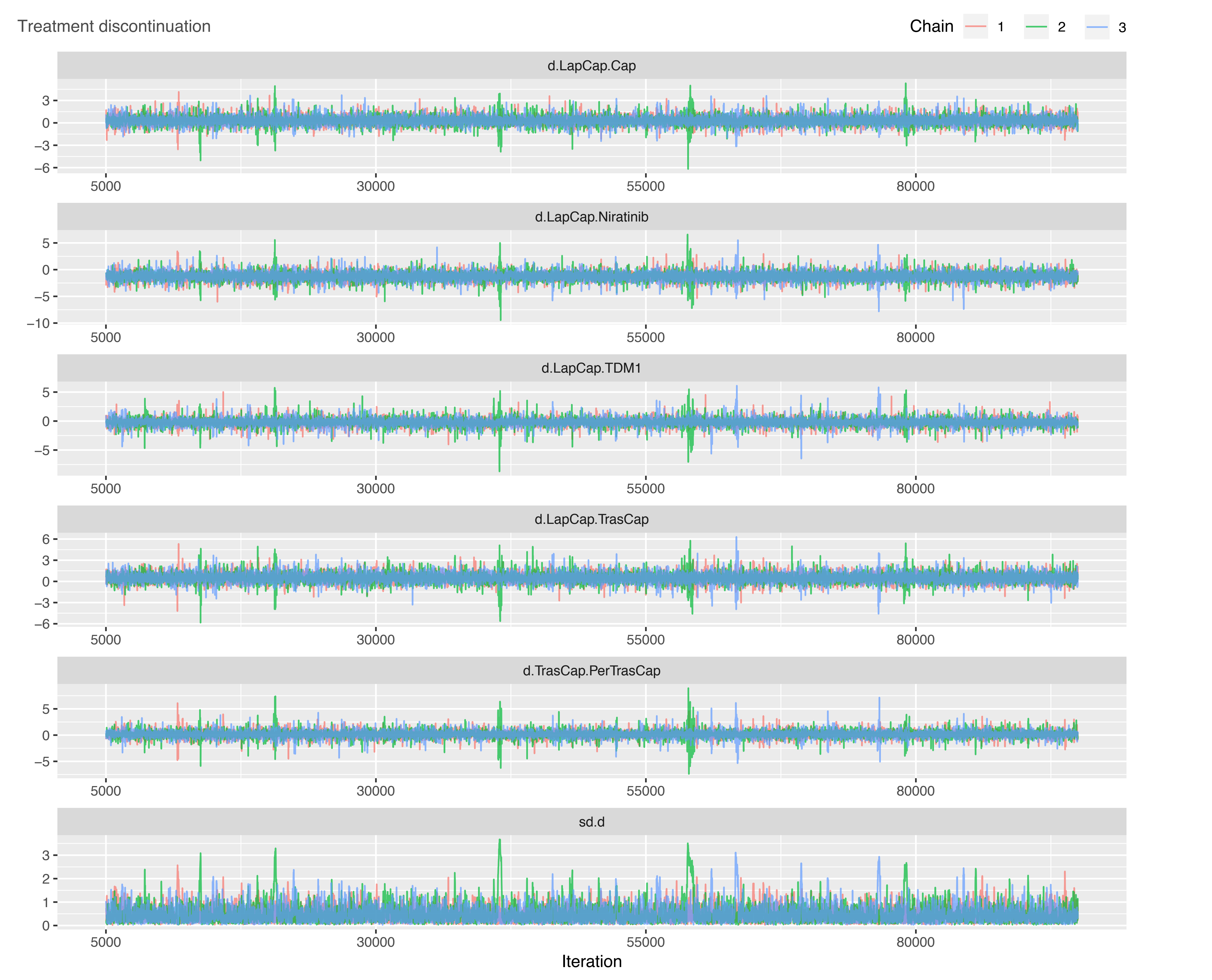
Treatment discontinuation
Chain
1
2
3
d.LapCap.Cap
d.LapCap.Niratinib
d.LapCap.TDM1
d.LapCap.TrasCap
d.TrasCap.PerTrasCap
sd.d
5000
30000
55000
80000
Iteration

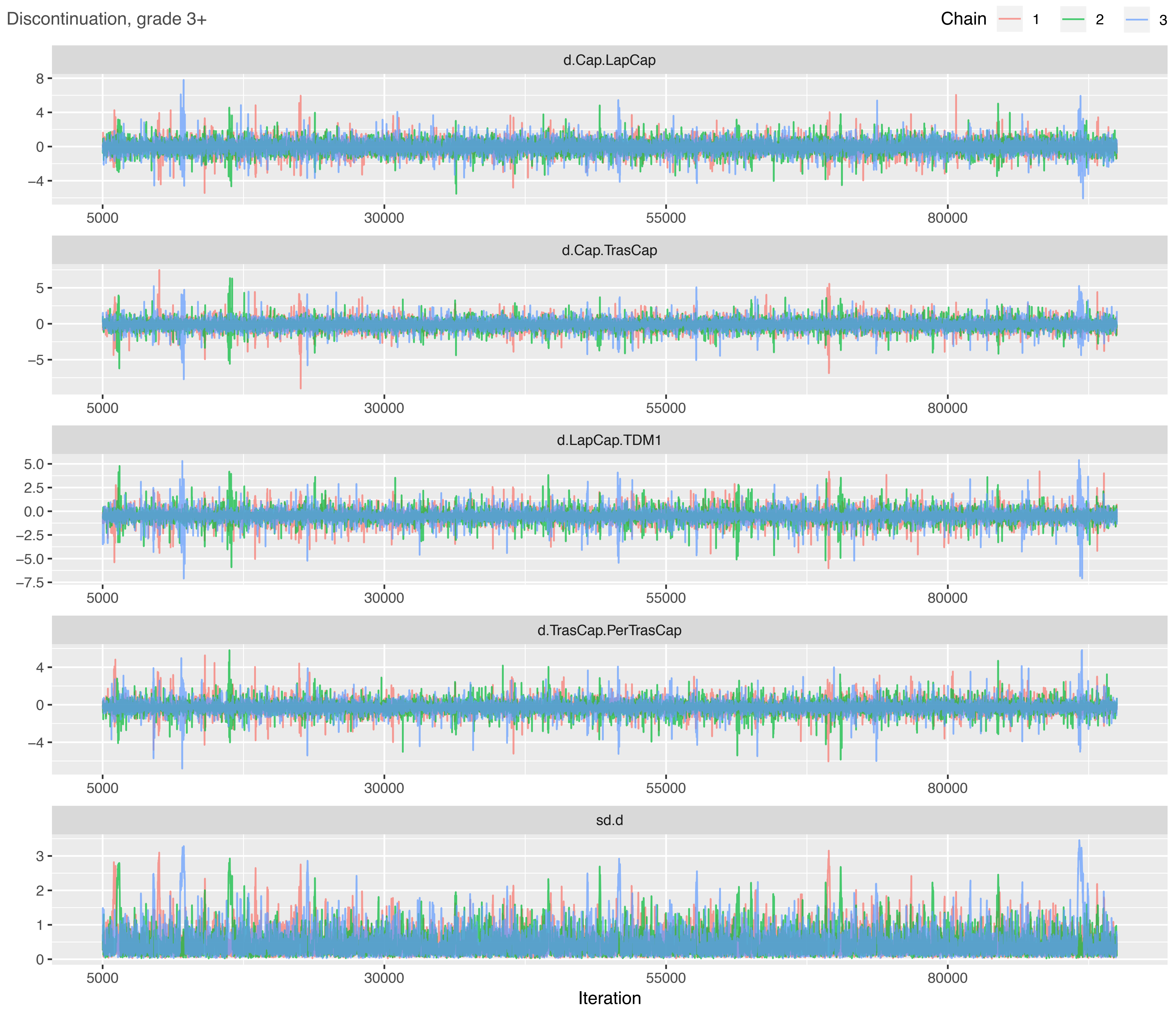

Discontinuation, grade 3+
Chain
1
2
3
d.Cap.LapCap
d.Cap.TrasCap
d.LapCap.TDM1
d.TrasCap.PerTrasCap
sd.d
8
4
0
−4
5
0
−5
5.0
2.5
0.0
−2.5
−5.0
−7.5
4
0
−4
3
2
1
0
5000
30000
55000
80000
Iteration

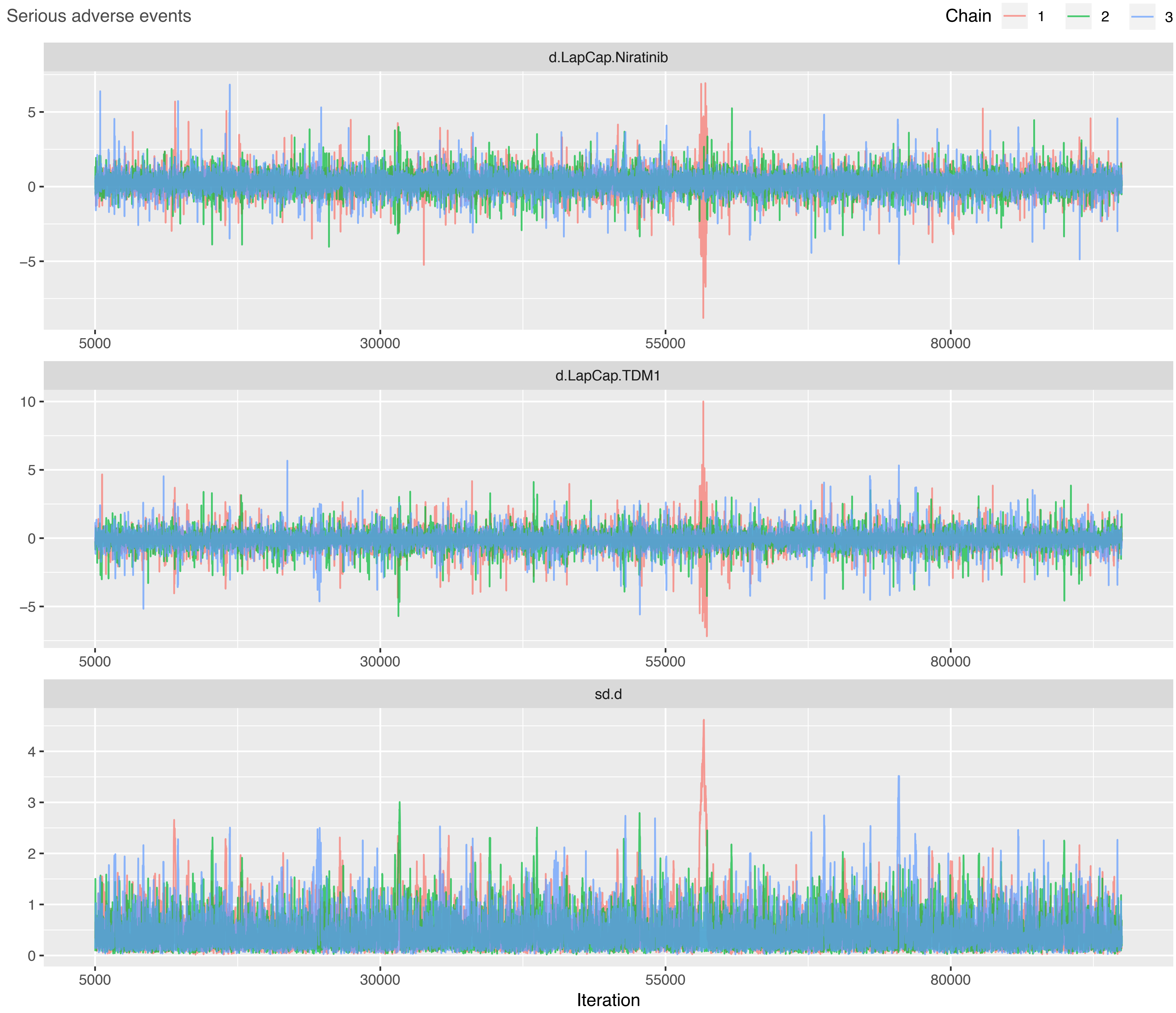

Serious adverse events
Chain
1
2
3
d.LapCap.Niratinib
d.LapCap.TDM1
sd.d
5000
30000
55000
80000
Iteration

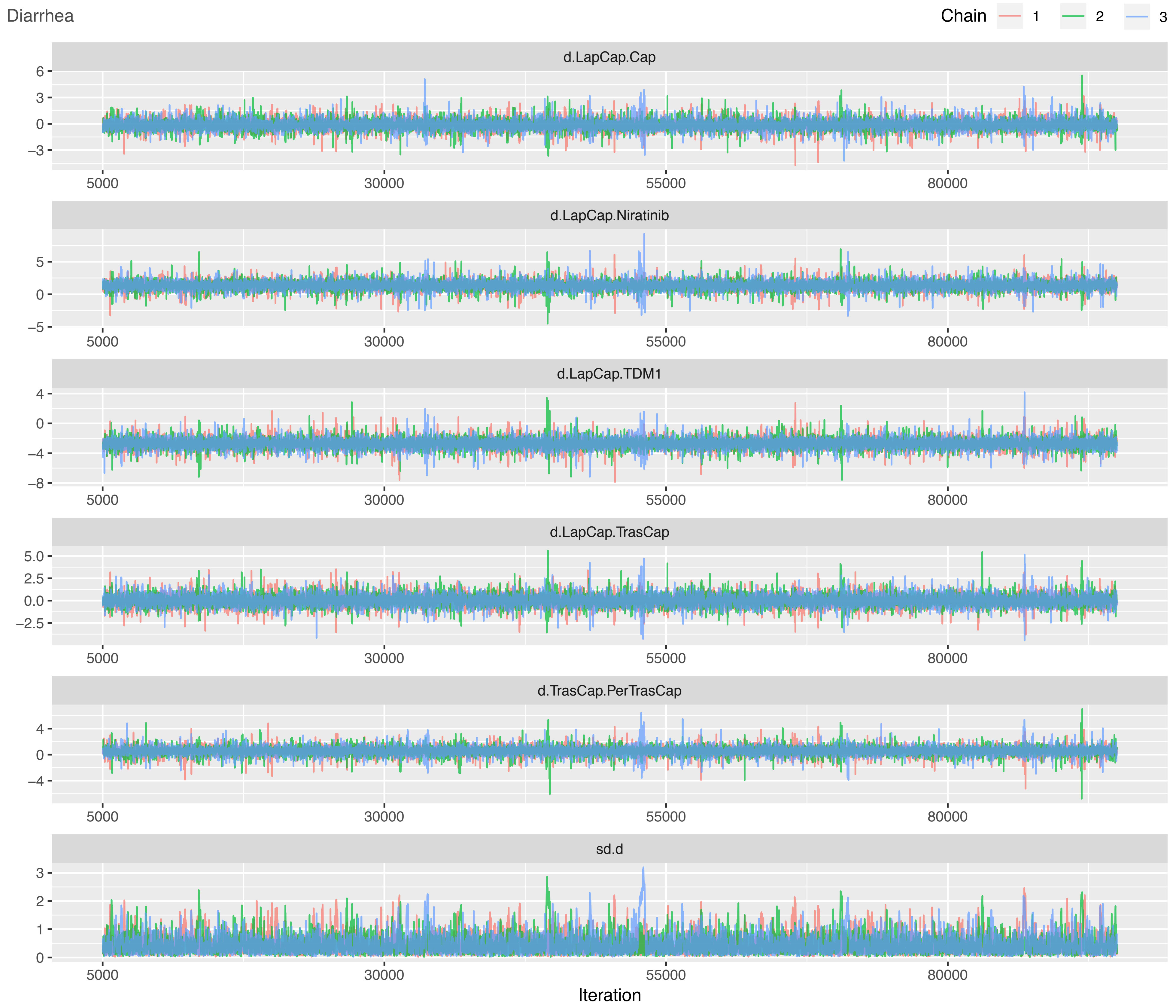

Diarrhea
Chain
1
2
3
d.LapCap.Cap
d.LapCap.Niratinib
d.LapCap.TDM1
d.LapCap.TrasCap
d.TrasCap.PerTrasCap
sd.d
5000
30000
55000
80000
Iteration

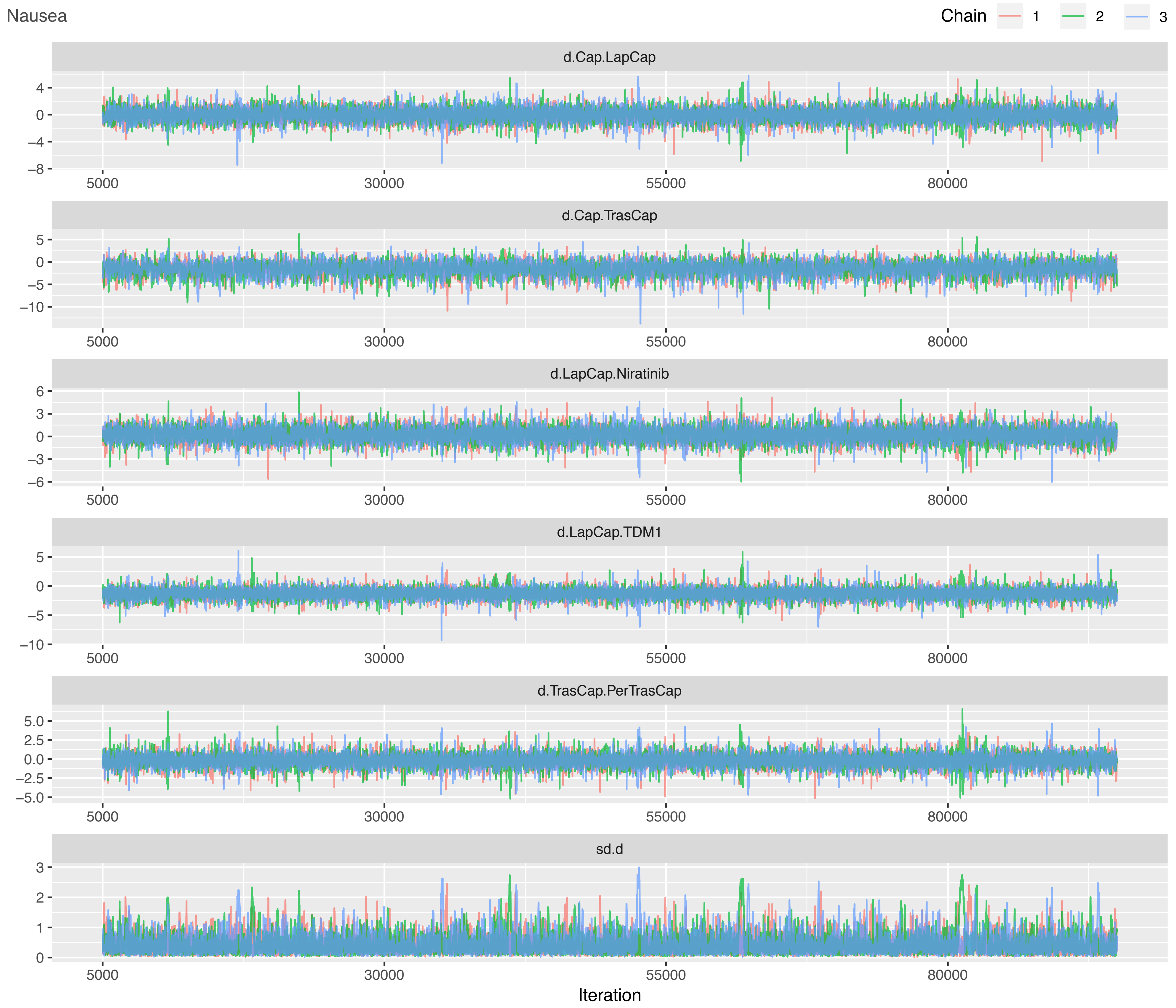

Nausea
Chain 1 2 3
d.Cap.LapCap
d.Cap.TrasCap
d.LapCap.Niratinib
d.LapCap.TDM1
d.TrasCap.PerTrasCap
sd.d
5000
30000
55000
80000
Iteration

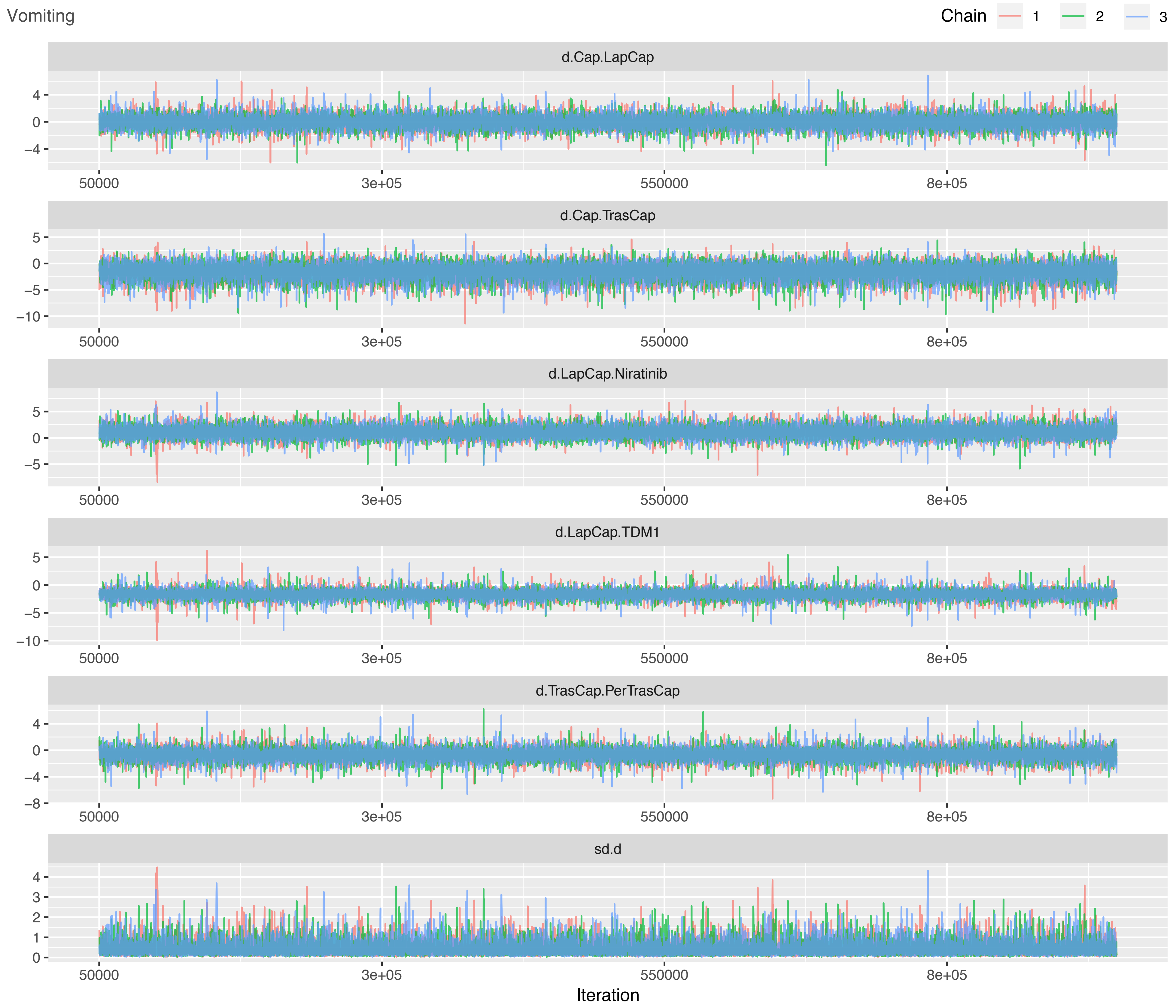
Vomiting
Chain
1
2
3
d.Cap.LapCap
d.Cap.TrasCap
d.LapCap.Niratinib
d.LapCap.TDM1
d.TrasCap.PerTrasCap
sd.d
50000
3e+05
550000
8e+05
Iteration

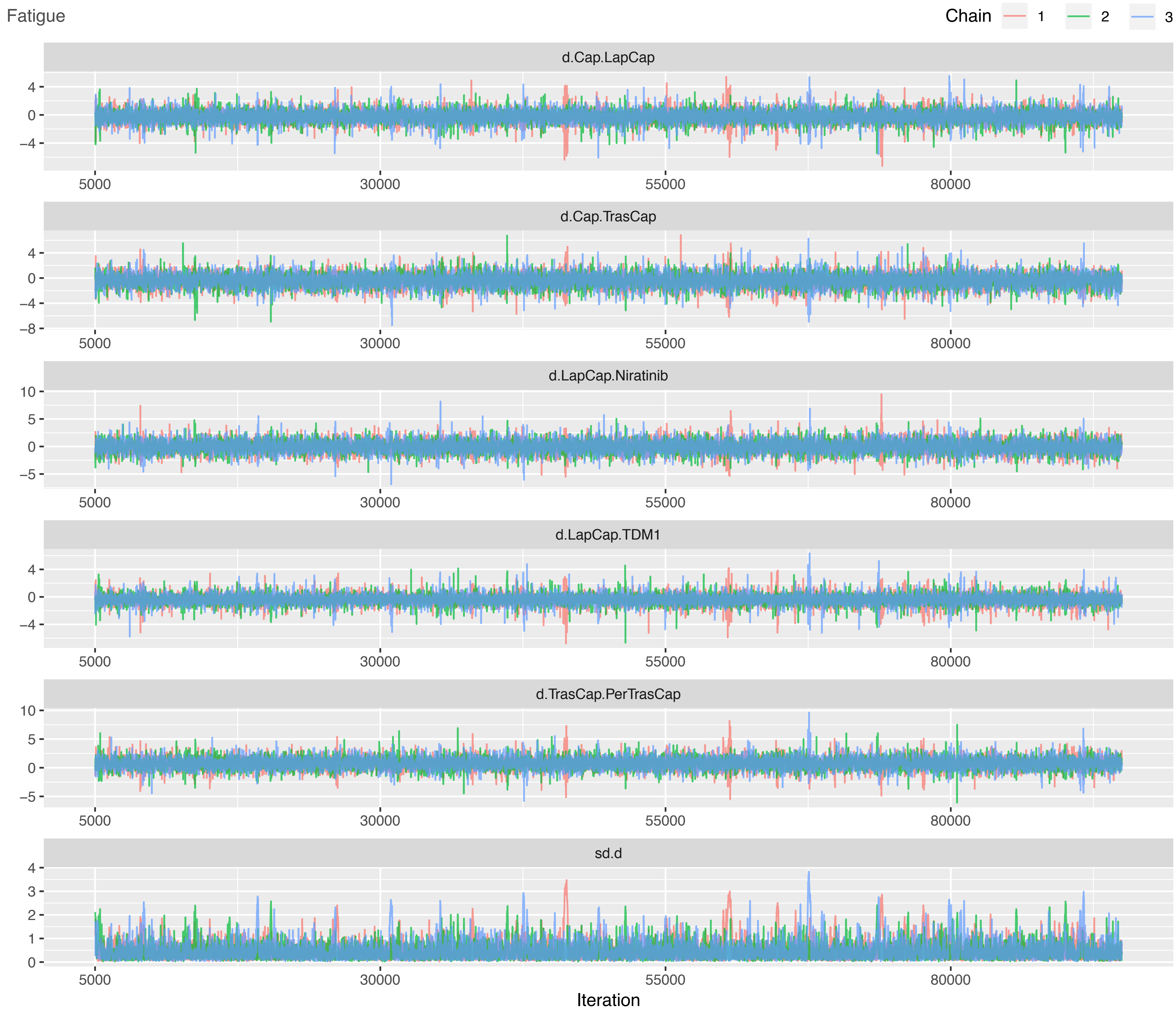

Fatigue
Chain
1
2
3
d.Cap.LapCap
d.Cap.TrasCap
d.LapCap.Niratinib
d.LapCap.TDM1
d.TrasCap.PerTrasCap
sd.d
5000
30000
55000
80000
Iteration

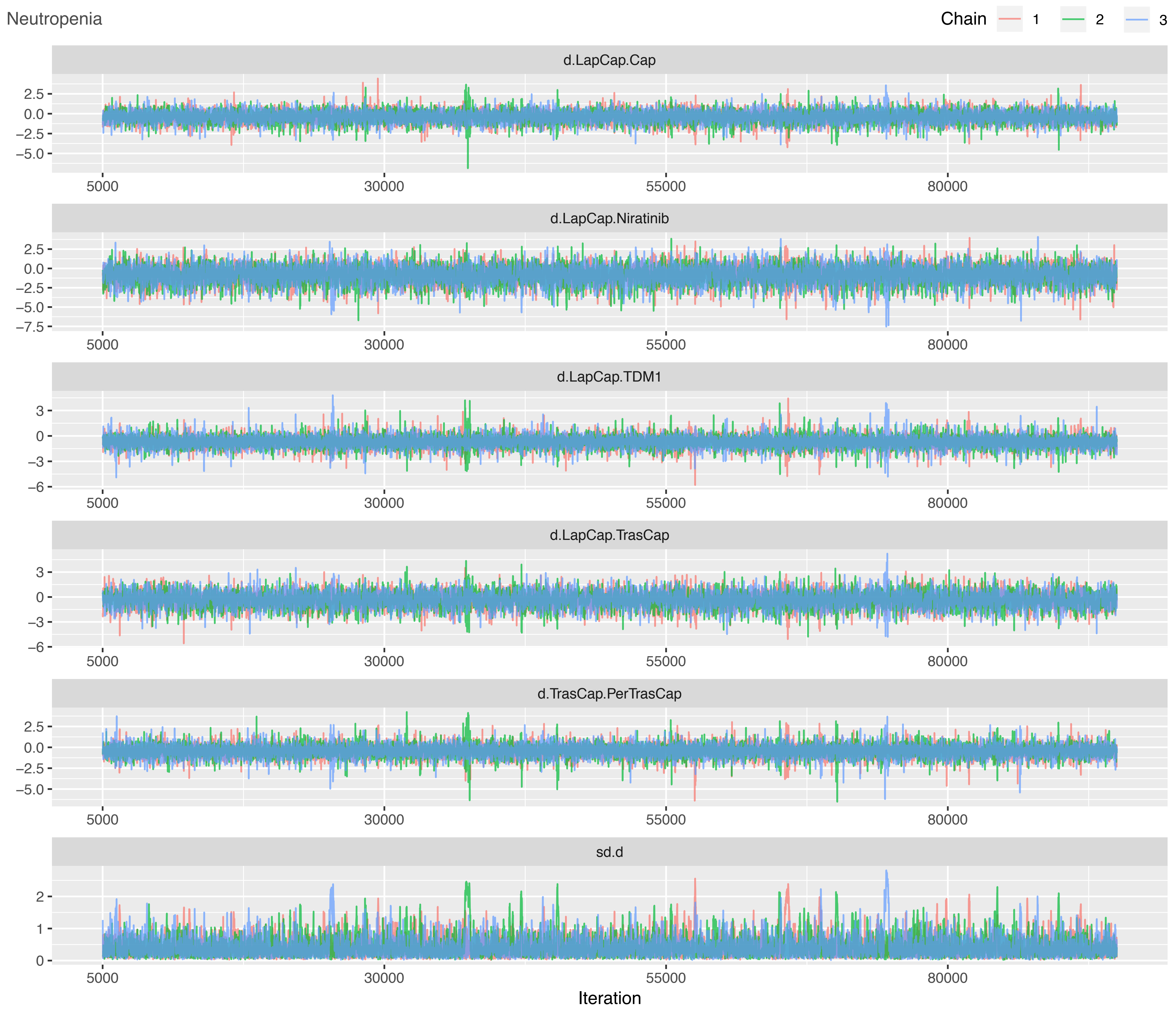
Neutropenia
Chain 1 2 3
d.LapCap.Cap
d.LapCap.Niratinib
d.LapCap.TDM1
d.LapCap.TrasCap
d.TrasCap.PerTrasCap
sd.d
5000
30000
55000
80000
Iteration

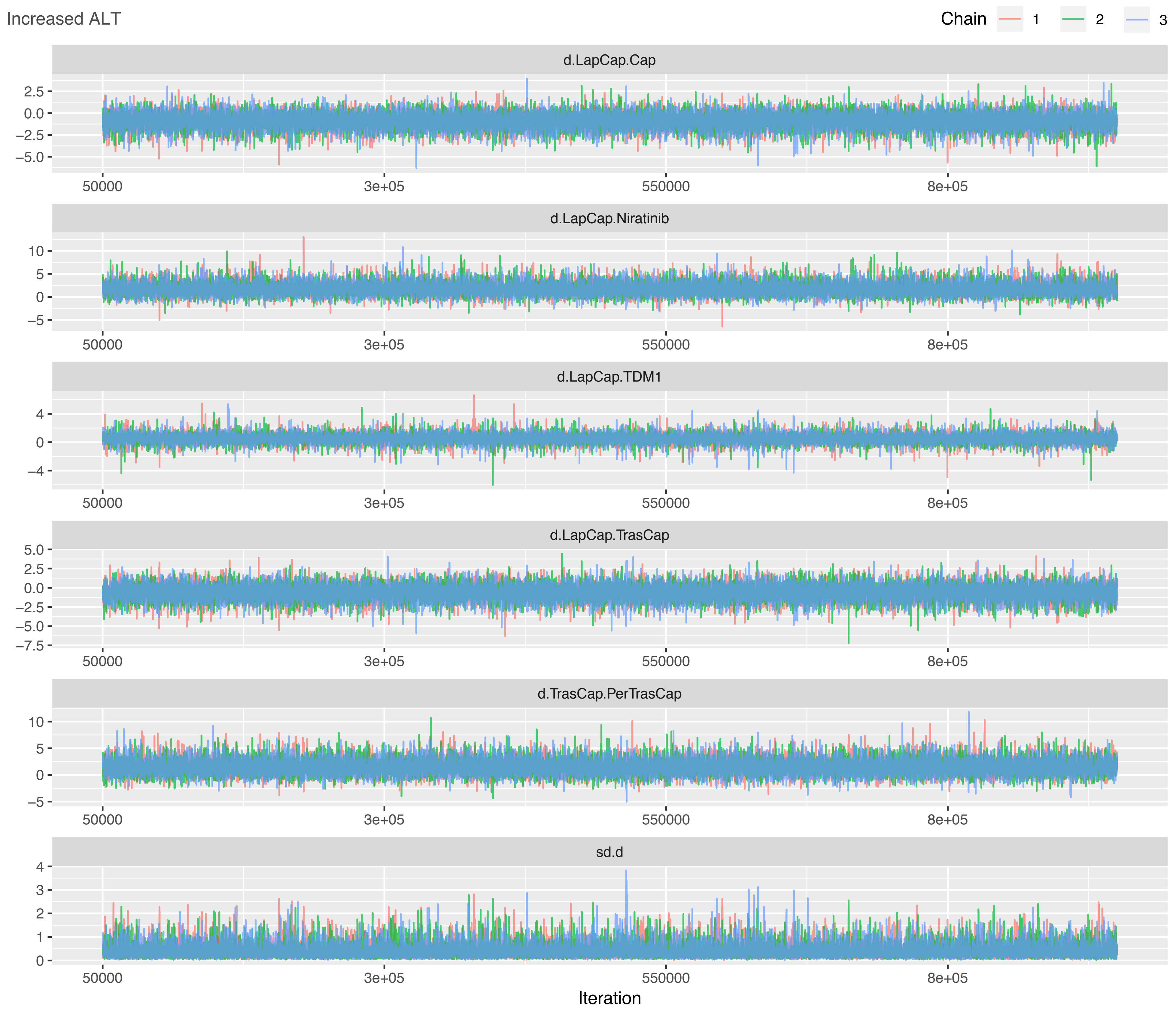

Increased ALT
Chain
1
2
3
d.LapCap.Cap
2.5
0.0
−2.5
−5.0
d.LapCap.Niratinib
10
5
0
−5
d.LapCap.TDM1
4
0
−4
d.LapCap.TrasCap
5.0
2.5
0.0
−2.5
−5.0
−7.5
d.TrasCap.PerTrasCap
10
5
0
−5
sd.d
4
3
2
1
0
50000
3e+05
550000
8e+05
Iteration

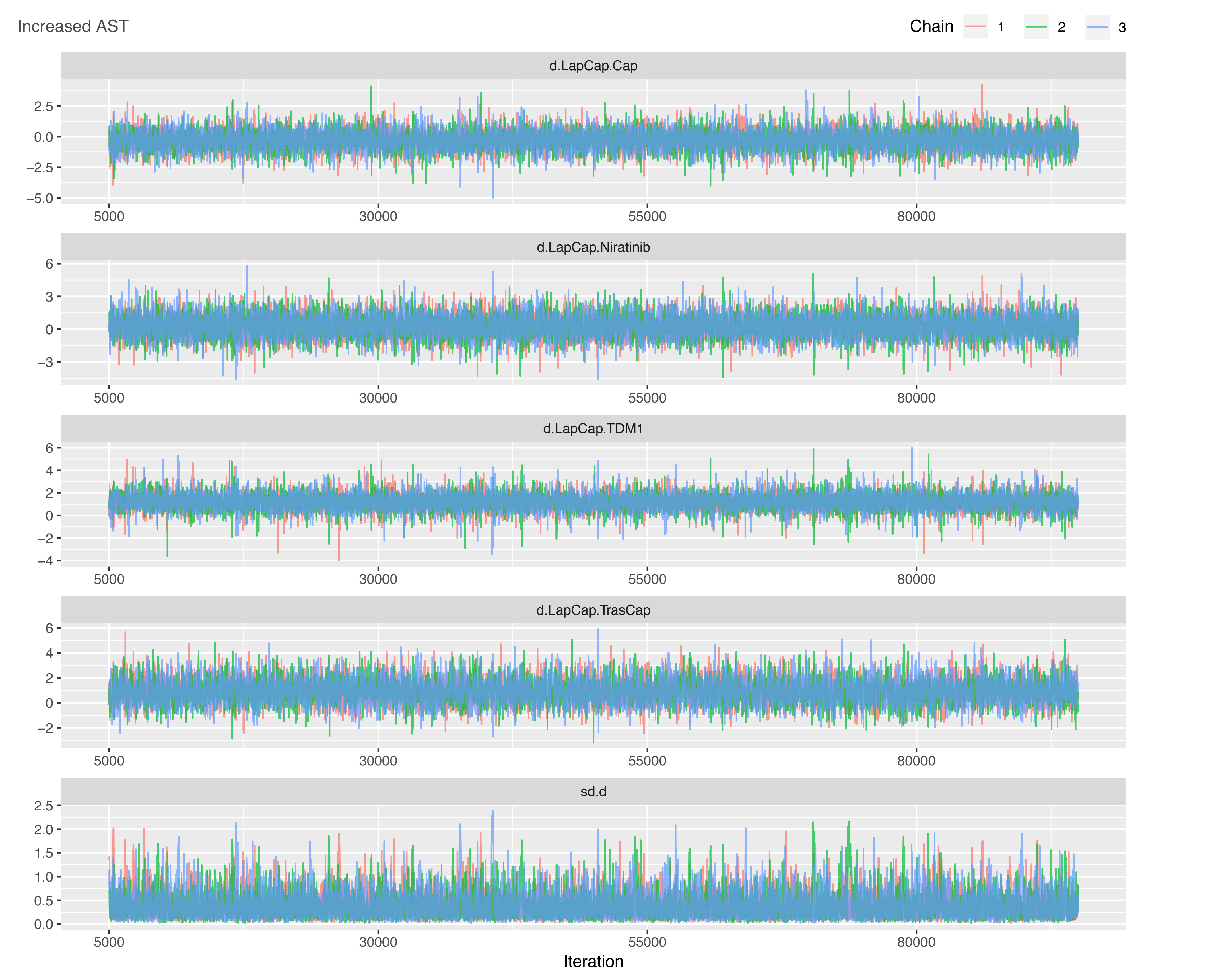

Increased AST
Chain
1
2
3
d.LapCap.Cap
2.5
0.0
−2.5
−5.0
d.LapCap.Niratinib
6
3
0
−3
d.LapCap.TDM1
6
4
2
0
−2
−4
d.LapCap.TrasCap
6
4
2
0
−2
sd.d
2.5
2.0
1.5
1.0
0.5
0.0
5000
30000
55000
80000
Iteration

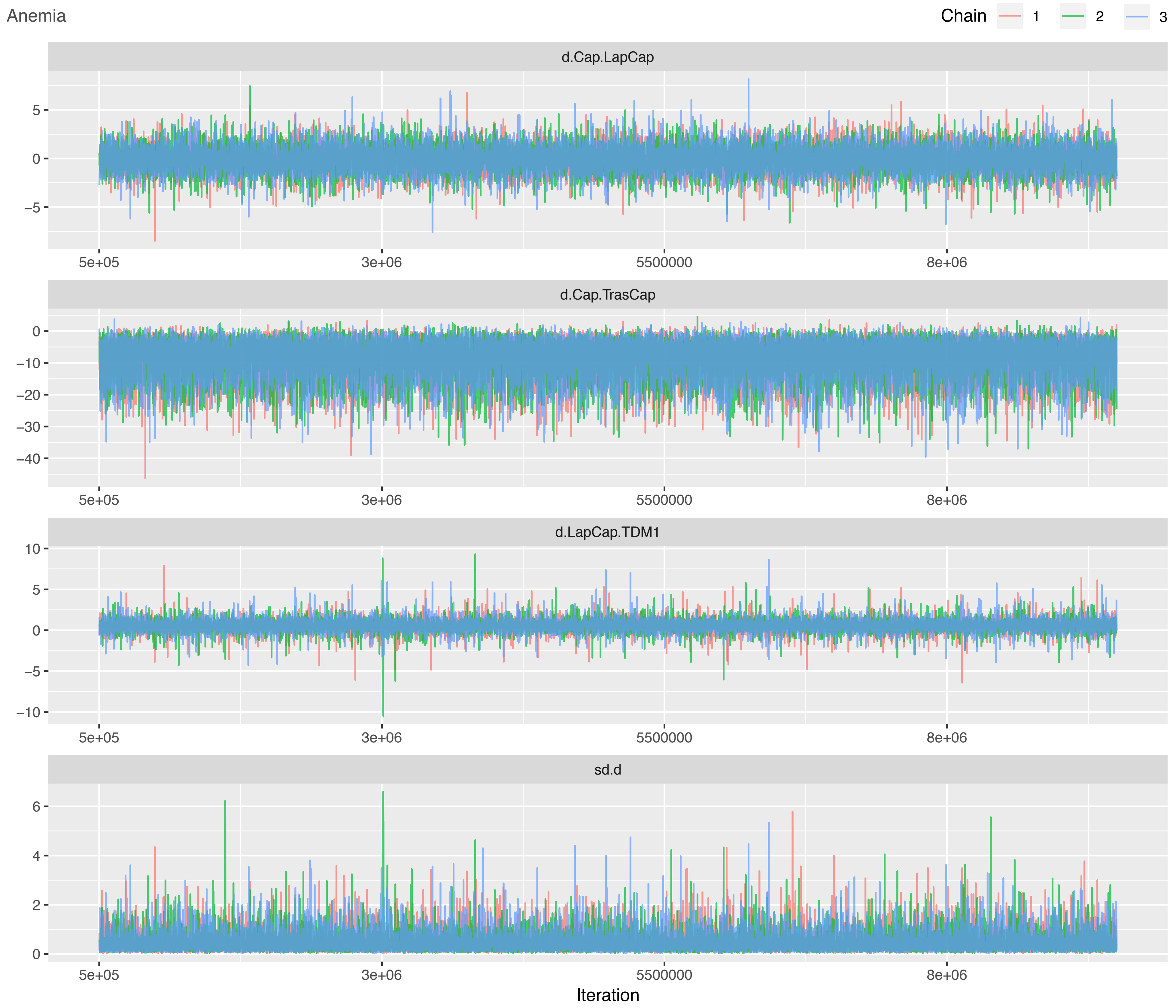

Anemia
Chain 1 2 3
d.Cap.LapCap
d.Cap.TrasCap
d.LapCap.TDM1
sd.d
5e+05
3e+06
5500000
8e+06
Iteration

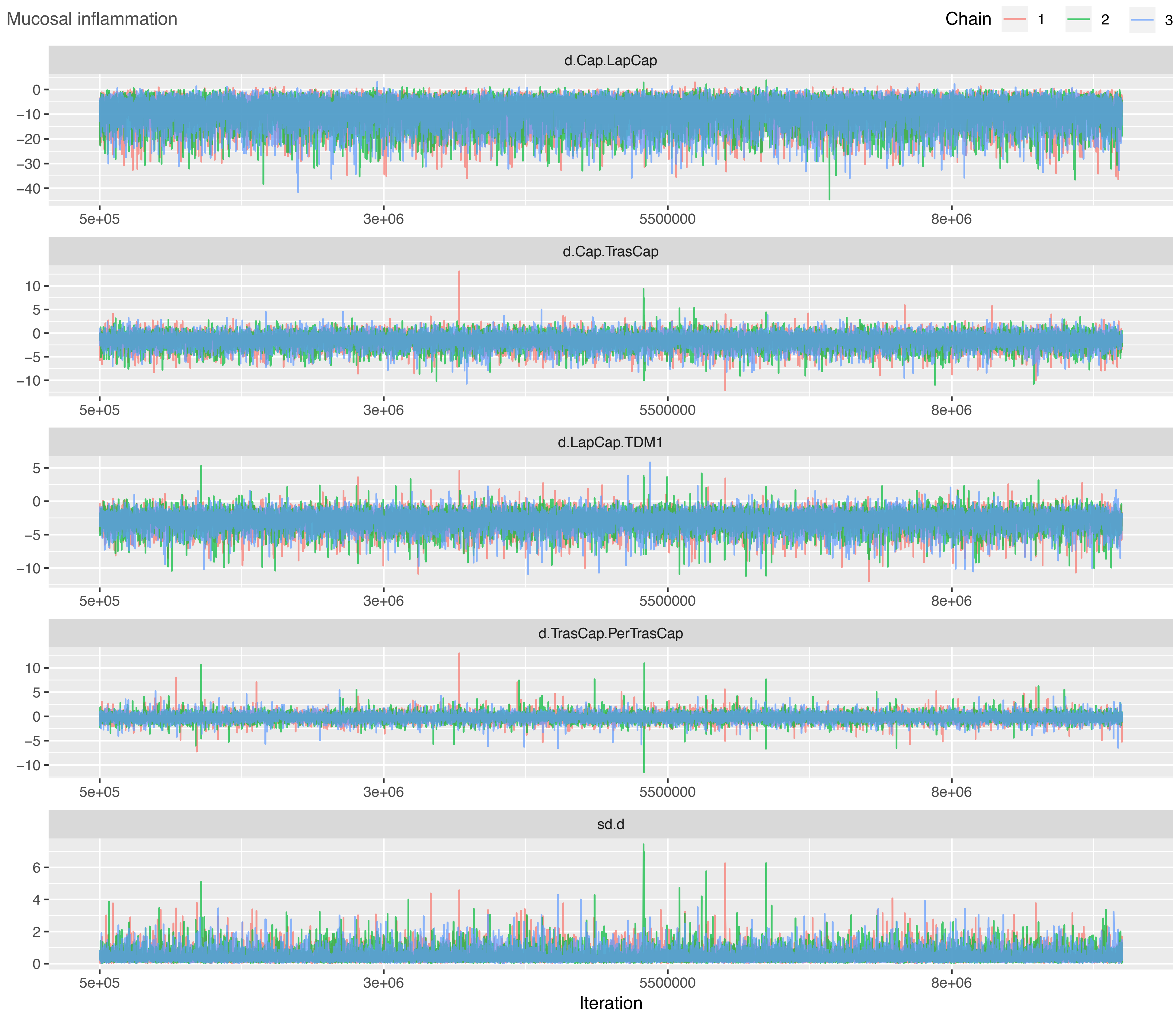

Mucosal inflammation
Chain
1
2
3
d.Cap.LapCap
d.Cap.TrasCap
d.LapCap.TDM1
d.TrasCap.PerTrasCap
sd.d
5e+05
3e+06
5500000
8e+06
Iteration

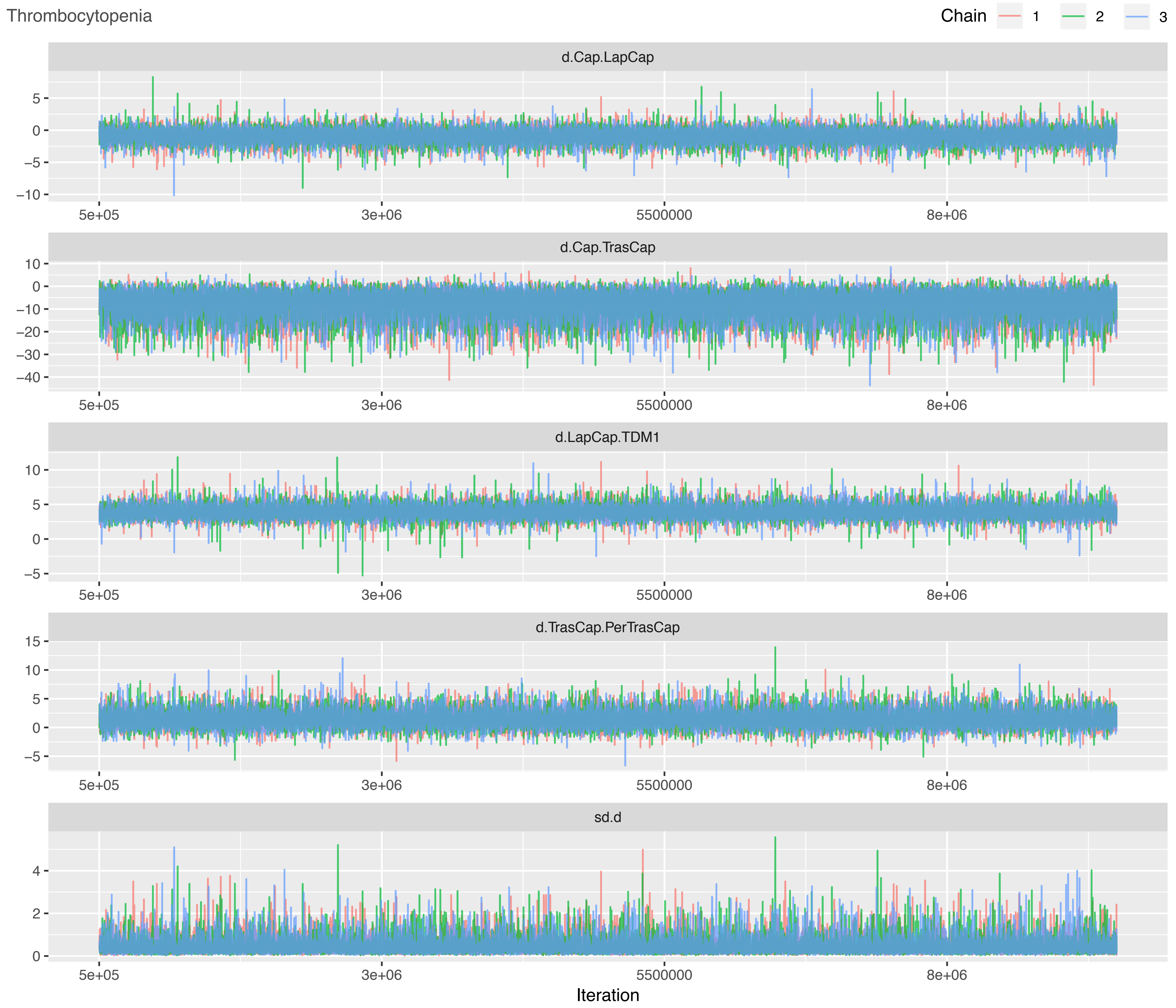

Thrombocytopenia
Chain 1 2 3
d.Cap.LapCap
d.Cap.TrasCap
d.LapCap.TDM1
d.TrasCap.PerTrasCap
sd.d
5e+05
3e+06
5500000
8e+06
Iteration
